# Genetic Determinants of Lipid Traits in Diverse Populations from the Population Architecture using Genomics and Epidemiology (PAGE) Study

**DOI:** 10.1371/journal.pgen.1002138

**Published:** 2011-06-30

**Authors:** Logan Dumitrescu, Cara L. Carty, Kira Taylor, Fredrick R. Schumacher, Lucia A. Hindorff, José L. Ambite, Garnet Anderson, Lyle G. Best, Kristin Brown-Gentry, Petra Bůžková, Christopher S. Carlson, Barbara Cochran, Shelley A. Cole, Richard B. Devereux, Dave Duggan, Charles B. Eaton, Myriam Fornage, Nora Franceschini, Jeff Haessler, Barbara V. Howard, Karen C. Johnson, Sandra Laston, Laurence N. Kolonel, Elisa T. Lee, Jean W. MacCluer, Teri A. Manolio, Sarah A. Pendergrass, Miguel Quibrera, Ralph V. Shohet, Lynne R. Wilkens, Christopher A. Haiman, Loïc Le Marchand, Steven Buyske, Charles Kooperberg, Kari E. North, Dana C. Crawford

**Affiliations:** 1 Center for Human Genetics Research, Vanderbilt University, Nashville, Tennessee, United States of America; 2 Public Health Sciences, Fred Hutchinson Cancer Research Center, Seattle, Washington, United States of America; 3 Department of Epidemiology, University of North Carolina, Chapel Hill, North Carolina, United States of America; 4 Department of Preventive Medicine, Keck School of Medicine, University of Southern California, Los Angeles, California, United States of America; 5 Office of Population Genomics, National Human Genome Research Institute, Bethesda, Maryland, United States of America; 6 Information Sciences Institute, University of Southern California, Los Angeles, California, United States of America; 7 Missouri Breaks Industries Research, Timber Lake, South Dakota, United States of America; 8 Department of Biostatistics, University of Washington, Seattle, Washington, United States of America; 9 Sponsored Programs, Baylor College of Medicine, Houston, Texas, United States of America; 10 Department of Genetics, Southwest Foundation for Biomedical Research, San Antonio, Texas, United States of America; 11 Department of Medicine, Weill Cornell Medical College, New York, New York, United States of America; 12 The Translational Genomics Research Institute, Phoenix, Arizona, United States of America; 13 Department of Family Medicine and Community Health, Alpert Medical School of Brown University School of Medicine, Providence, Rhode Island, United States of America; 14 Institute of Molecular Medicine, University of Texas Health Sciences Center at Houston, Texas, United States of America; 15 Division of Epidemiology, School of Public Health, University of Texas Health Sciences Center, Houston, Texas, United States of America; 16 Medstar Research Institute, Washington, D.C., United States of America; 17 Department of Preventive Medicine, University of Tennessee Health Science Center, Memphis, Tennessee, United States of America; 18 Epidemiology Program, University of Hawaii Cancer Center, Department of Medicine, John A. Burns School of Medicine, University of Hawaii, Honolulu, Hawaii, United States of America; 19 University of Oklahoma Health Sciences Center, Oklahoma City, Oklahoma, United States of America; 20 School of Public Health, University of North Carolina, Chapel Hill, North Carolina, United States of America; 21 Center of Cardiovascular Research, Department of Medicine, John A. Burns School of Medicine, University of Hawaii, Honolulu, Hawaii, United States of America; 22 Department of Statistics and Biostatistics, Rutgers University, Piscataway, New Jersey, United States of America; 23 Carolina Center for Genome Sciences, University of North Carolina, Chapel Hill, North Carolina, United States of America; 24 Department of Molecular Physiology and Biophysics, Vanderbilt University, Nashville, Tennessee, United States of America; Georgia Institute of Technology, United States of America

## Abstract

For the past five years, genome-wide association studies (GWAS) have identified hundreds of common variants associated with human diseases and traits, including high-density lipoprotein cholesterol (HDL-C), low-density lipoprotein cholesterol (LDL-C), and triglyceride (TG) levels. Approximately 95 loci associated with lipid levels have been identified primarily among populations of European ancestry. The Population Architecture using Genomics and Epidemiology (PAGE) study was established in 2008 to characterize GWAS–identified variants in diverse population-based studies. We genotyped 49 GWAS–identified SNPs associated with one or more lipid traits in at least two PAGE studies and across six racial/ethnic groups. We performed a meta-analysis testing for SNP associations with fasting HDL-C, LDL-C, and ln(TG) levels in self-identified European American (∼20,000), African American (∼9,000), American Indian (∼6,000), Mexican American/Hispanic (∼2,500), Japanese/East Asian (∼690), and Pacific Islander/Native Hawaiian (∼175) adults, regardless of lipid-lowering medication use. We replicated 55 of 60 (92%) SNP associations tested in European Americans at p<0.05. Despite sufficient power, we were unable to replicate *ABCA1* rs4149268 and rs1883025, *CETP* rs1864163, and *TTC39B* rs471364 previously associated with HDL-C and *MAFB* rs6102059 previously associated with LDL-C. Based on significance (p<0.05) and consistent direction of effect, a majority of replicated genotype-phentoype associations for HDL-C, LDL-C, and ln(TG) in European Americans generalized to African Americans (48%, 61%, and 57%), American Indians (45%, 64%, and 77%), and Mexican Americans/Hispanics (57%, 56%, and 86%). Overall, 16 associations generalized across all three populations. For the associations that did not generalize, differences in effect sizes, allele frequencies, and linkage disequilibrium offer clues to the next generation of association studies for these traits.

## Author Summary

Low-density lipoprotein cholesterol (LDL-C), high-density lipoprotein cholesterol (HDL-C), and triglyceride (TG) levels are well known independent risk factors for cardiovascular disease. Lipid-associated genetic variants are being discovered in genome-wide association studies (GWAS) in samples of European descent, but an insufficient amount of data exist in other populations. Therefore, there is a strong need to characterize the effect of these GWAS–identified variants in more diverse cohorts. In this study, we selected over forty genetic loci previously associated with lipid levels and tested for replication in a large European American cohort. We also investigated if the effect of these variants generalizes to non-European descent populations, including African Americans, American Indians, and Mexican Americans/Hispanics. A majority of these GWAS–identified associations replicated in our European American cohort. However, the ability of associations to generalize across other racial/ethnic populations varied greatly, indicating that some of these GWAS–identified variants may not be functional and are more likely to be in linkage disequilibrium with the functional variant(s).

## Introduction

Since its introduction in 2005, the genome-wide association study (GWAS) design has become a powerful tool in human genetics to identify single nucleotide polymorphisms (SNPs) associated with common diseases or traits using an experimental design that does not require *a priori* biological knowledge. As of September 2010, greater than 1,000 SNPs across the genome have been reported as genome-wide significant (p≤5×10^−8^) for 165 traits [1]. An early analysis of the GWAS-reported SNPs demonstrated that most identified variants were intergenic or intronic [2], suggesting either novel biology or that the functional variant has yet to be found.

While GWAS have been successful in identifying novel associations, there are several limitations. First, the majority of GWAS have been conducted in populations of European-descent. There are several GWAS in populations of Asian-descent, and GWAS are just emerging for other populations such as African Americans [3]–[20], Mexican Americans/Hispanics [9], [20]–[26], and American Indians [27]. It is possible that novel associations await discovery in these populations given the differing linkage disequilibrium (LD) patterns when compared with populations of European-descent [28]. Second, much work is needed to test SNPs discovered in case-control studies in more population-based, representative cohorts to determine if the associations generalize. Data on generalization will inform future fine-mapping [29] and discovery studies as well as provide clues to whether GWAS-identified SNPs are simply tagSNPs or are more likely to be true functional SNP(s).

A major goal of the Population Architecture using Genomics and Epidemiology (PAGE) study is to determine whether GWAS-identified variants generalize to diverse groups drawn from population-based studies [30]. Generalization is defined here as a significant association (p<0.05, uncorrected for multiple testing) in a non-European population and a direction of genetic effect in the same direction as that of European Americans. In PAGE, variants identified in GWAS and well replicated in multiple studies are chosen for targeted genotyping in hundreds to thousands of European Americans (∼20,000), African Americans (∼9,000), American Indians (∼6,000), Mexican Americans/Hispanics (∼2,500), Japanese/East Asians (∼690), and Native Hawaiians/Pacific Islanders (∼175). All samples are linked to extensive demographic, health, and exposure data, making the PAGE study a rich resource for post-discovery generalization and characterization for common human diseases and traits.

We present here PAGE study data on the replication and generalization for 49 SNPs associated with three common lipid traits: low-density lipoprotein cholesterol (LDL-C), high-density lipoprotein cholesterol (HDL-C), and triglycerides. Each of these three traits has numerous GWAS published in European ancestry individuals [30]–[43] but only a handful published in other populations (such as Asians [44] and Micronesians [45]). Additional data are just now emerging from large sample sizes of diverse populations for generalization [32], [46]–[51] and fine-mapping [52] of these lipid GWAS-identified SNPs. We demonstrate that the majority of the targeted GWAS-identified SNPs replicate in European Americans in PAGE and that many generalize to diverse populations. Both power and LD are explored as explanations of non-generalization, highlighting the complexities involved in properly interpreting results of even robust genetic associations such as these.

## Results

### Study population characteristics

The PAGE study sites are diverse across multiple variables (Table 1 and Table S1). Together, the PAGE study consists of several populations: European Americans, African Americans, Mexican Americans/Hispanics, American Indians, Japanese/East Asians, and Native Hawaiians/Pacific Islanders. All PAGE study sites except WHI ascertained both men and women. Participant age varies widely across PAGE. For example, CHS ascertained on average older adults (median age  = 74 and 72 years for European and African Americans, respectively), CARDIA ascertained younger adults (median age  = 26 and 24.5 years for European and African Americans, respectively), and NHANES ascertained all ages of adults (18 years to 90 years; median age  = 51, 39, and 40 years for European, African, and Mexican Americans, respectively). In addition to demographic differences, lifestyles and health differed across the PAGE study sites by population, including lipid lowering medication use and current smoking status. More Japanese participants ascertained by MEC reported lipid lowering medication use compared with other populations ascertained by other PAGE study sites: 38.3% versus <5–10%. American Indians from the Dakotas reported more smoking (42.2–47.8%) than other American Indians (25–33%) or other PAGE study site populations (6.3% to 35.3%). The differences in demographics, lifestyle, and health characteristics observed across the PAGE study sites and populations are reflected in the three traits studied here (Table S1). Given the diversity observed across the PAGE study sites, we performed all tests of association for HDL-C, LDL-C, and triglycerides unadjusted, minimally adjusted (for age and sex), and adjusted for various demographic, lifestyle, and health variables.

**Table 1:** Characteristics of PAGE study populations.

	EAGLE	MEC	WHI	CALiCo			
				ARIC	CARDIA	CHS	SHS
**Type of Study**	Cross-sectional	Nested Case Control	Cohort and Clinical Trials	Longitudinal	Longitudinal	Longitudinal	Longitudinal
**Focus of Cohort**	N/A	Cancer*	Women's Health	Cardiovascular Disease	Cardiovascular Disease	Cardiovascular Disease	Cardiovascular Disease
**Years Collected**	1991–1994, 1999–2002	1993–1996	1993–1998	1987–2007	1986–2006	1989–1999	1988–present
**Median Age**	43	67	63	54	25	73	47
**Age Range**	18–90	48–86	50–79	44–66	18–35	64–96	14–93
**% Women**	54	36	100	57	56	62	59.3
**Race/Ethnicity(n_max_)**							
European Americans	3,909	317	4,688	11,178	2,134	2,787	–
African Americans	1,896	552	1,840	3,770	2,035	550	–
American Indians	–	–	113	–	–	–	6,021
Mexican Americans	2,361	299	762	–	–	–	–
Japanese/East Asian	–	576	251	–	–	–	–
Native Hawaiian/Pacific Islander	–	87	113	–	–	–	–

*Only controls (cancer-free participants) from the overall nested case-control study were included in this lipids study.

Epidemiologic Architecture for Genes Linked to Environment (EAGLE); Multiethnic Cohort (MEC); Women's Health Initiative (WHI); Causal Variants Across the Life Course (CALiCo); Atherosclerosis Risk in Communities (ARIC); Coronary Artery Risk Development in Young Adults (CARDIA); Cardiovascular Health Study (CHS); Strong Heart Study (SHS).

### Allele frequencies

Coded allele frequencies are presented in Table 2, Table 3, Table 4 and in Figure S1, by population. We calculated the Pearson correlation coefficient (r) and F_ST_ between European American coded allele frequencies and all other groups. The highest correlation was observed in the comparison with Mexican Americans/Hispanics (0.97) followed by American Indians (0.92), Native Hawaiians/Pacific Islanders (0.90), Japanese/East Asians (0.87), and African Americans (0.84). Compared with European Americans, the proportion of SNPs with F_ST_ values greater than 0.15 was smallest in Mexican Americans/Hispanics (0/49 SNPs) and largest in African Americans (6/49 SNPs; 12%) followed by Japanese/East Asians (5/46 SNPs, 11%). F_ST_ values were small for the remaining populations compared to European Americans, with 3% and 7% of SNPs with F_ST_ values greater than 0.15 for American Indians and Native Hawaiians/Pacific Islanders, respectively.

**Table 2:** Meta-analysis of GWAS–identified HDL-C SNPs.

SNP	Nearest Gene	CA	European Americans(n_max_ = 25,167)			African Americans(n_max_ = 10,436)			American Indians(n_max_ = 6,134)			Mexican Americans and Hispanics(n_max_ = 3,371)			G
			CAF	β (SE)	P-value	CAF	β (SE)	P-value	CAF	β (SE)	P-value	CAF	β (SE)	P-value	
rs2144300	*GALNT2*	T	0.60	0.59 (0.14)	3.33E-05	0.15	0.48 (0.31)	0.12	0.55	0.29 (0.25)	0.25	0.56	0.39 (0.34)	0.25	N
rs17145738	*MLXIPL*	T	0.12	0.91 (0.21)	1.64E-05	0.09	−0.28 (0.40)	0.46	0.08	0.46 (0.48)	0.34	0.07	0.25 (0.67)	0.71	N
rs328	*LPL*	C	0.90	−2.29 (0.24)	5.60E-22	0.93	−1.79 (0.52)	5.84E-04	0.97	−1.55 (0.85)	0.07	0.94	−2.31 (0.69)	8.80E-04	N
rs2197089	*LPL*	T	0.55	0.90 (0.13)	9.49E-11	0.78	0.95 (0.27)	4.79E-04	0.40	1.10 (0.26)	2.19E-05	0.47	1.22 (0.33)	2.56E-04	Y
rs6586891	*LPL*	A	0.66	0.96 (0.14)	5.88E-11	0.84	0.60 (0.30)	4.76E-02	0.44	0.76 (0.26)	2.86E-03	0.53	1.27 (0.34)	1.73E-04	Y
rs471364	*TTC39B*	A	0.89	0.35 (0.23)	0.13	0.81	0.24 (0.31)	0.45	0.97	0.43 (0.77)	0.58	0.92	−0.23 (0.69)	0.74	N
rs4149268	*ABCA1*	A	0.37	−0.30 (0.18)	0.12	0.67	−0.03 (0.35)	0.92	–	–	–	0.32	−0.17 (0.40)	0.67	N
rs3890182	*ABCA1*	A	0.12	−1.06 (0.20)	4.53E-07	0.12	−0.83 (0.34)	1.39E-02	0.05	-0.92 (0.72)	0.20	0.09	−0.24 (0.63)	0.70	N
rs1883025	*ABCA1*	A	0.26	−0.44 (0.38)	0.25	0.34	0.02 (0.56)	0.97	–	–	–	0.27	−0.59 (0.44)	0.18	N
rs174547	*FADS1*	T	0.66	0.84 (0.17)	1.14E-06	0.91	0.94 (0.42)	2.73E-02	0.21	0.56 (0.41)	0.17	0.39	1.17 (0.38)	1.98E-03	N
rs28927680	*APOA1/C3/A4/A5*	G	0.93	1.51 (0.26)	8.61E-09	0.84	−0.03 (0.30)	0.93	0.83	1.19 (0.37)	1.13E-03	0.86	1.00 (0.48)	3.98E-02	N
rs964184	*APOA1/C3/A4/A5*	C	0.86	1.57 (0.25)	6.08E-10	0.80	0.48 (0.39)	0.22	0.78	1.32 (2.48)	0.60	0.71	1.98 (0.38)	1.55E-07	N
rs3135506	*APOA1/C3/A4/A5*	C	0.06	−1.86 (0.31)	1.42E-09	0.06	−1.94 (0.60)	1.17E-03	0.17	−1.41 (0.37)	1.40E-04	0.14	−1.22 (0.54)	2.45E-02	Y
rs2338104	*MMAB-MVK*	C	0.46	−0.40 (0.14)	5.64E-03	0.27	−0.35 (0.27)	0.19	0.58	−0.03 (0.26)	0.91	0.52	−0.92 (0.38)	1.46E-02	N
rs4775041	*LIPC*	C	0.29	1.31 (0.16)	1.03E-16	0.14	0.79 (0.35)	2.55E-02	0.21	1.34 (0.47)	2.05E-05	0.18	1.34 (0.47)	4.66E-03	Y
rs261332	*LIPC*	A	0.20	1.76 (0.24)	1.99E-13	0.24	0.31 (0.43)	0.46	–	–	–	0.15	0.66 (0.72)	0.35	N
rs1864163	*CETP*	A	0.23	−2.07 (1.36)	0.13	0.27	−2.79 (1.02)	6.19E-03	–	–	–	0.28	−2.98 (1.26)	1.78E-02	N
rs12596776	*CETP*	C	0.90	−1.36 (0.31)	1.18E-05	0.94	−0.48 (0.70)	0.50	–	–	–	0.94	−0.13 (0.75)	0.86	N
rs9989419	*CETP*	A	0.39	−2.17 (0.14)	1.71E-53	0.59	0.02 (0.24)	0.93	0.26	−1.62 (0.30)	4.42E-08	0.32	−2.29 (0.39)	5.29E-09	N
rs3764261	*CETP*	T	0.32	3.64 (0.15)	8.83E-129	0.32	2.79 (0.25)	5.98E-28	0.31	2.81 (0.27)	5.00E-25	0.33	2.68 (0.40)	2.53E-11	Y
rs1566439	*CETP*	A	0.60	−0.54 (0.16)	1.07E-03	0.78	0.16 (0.37)	0.67	–	–	–	0.53	−0.42 (0.37)	0.25	N
rs2271293	*LCAT*	A	0.12	1.45 (0.22)	8.40E-11	0.68	1.11 (0.43)	1.05E-02	0.26	1.26 (0.29)	1.65E-05	0.14	0.99 (0.52)	5.65E-02	N
rs2156552	*LIPG*	T	0.17	−1.27 (0.19)	5.11E-11	0.04	−0.59 (0.62)	0.34	0.05	−0.96 (0.69)	0.17	0.08	−0.49 (0.68)	0.47	N
rs2967605	*ANGPTL4*	A	0.18	−0.90 (0.18)	1.12E-06	0.21	−0.89 (0.29)	2.24E-03	0.30	−0.26 (0.28)	0.35	0.23	−0.67 (0.43)	0.12	N
rs4420638	*APOE/C1/C4*	A	0.82	1.00 (0.20)	5.69E-07	0.80	−1.01 (0.35)	4.29E-03	0.90	1.38 (0.48)	3.95E-03	0.90	1.45 (0.59)	1.47E-02	N
rs1800961	*HNF4A*	T	0.03	−1.14 (0.41)	5.78E-03	0.01	−1.01 (1.46)	0.49	0.03	−1.43 (0.73)	0.05	0.04	−2.33 (0.95)	1.42E-02	N
rs7679	*PLTP*	T	0.82	0.95 (0.21)	8.42E-06	0.96	0.01 (0.58)	0.99	0.94	0.31 (0.58)	0.60	0.89	0.89 (0.60)	0.14	N

Coded allele (CA); coded allele frequency (CAF); beta coefficient (β); standard error (SE); data not available (–); generalized (G); yes (Y); no (N). Generalization is defined here as a significant association (p<0.05) and a similar direction of effect (β) compared with European Americans for the same test of association, across all racial/ethnic populations.

**Table 3:** Meta-analysis of GWAS–identified LDL-C SNPs.

SNP	Nearest Gene	CA	European Americans(n_max_ = 21,986)			African Americans(n_max_ = 9,328)			American Indians(n_max_ = 6,144)			Mexican Americans and Hispanics(n_max_ = 2,532)			G
			CAF	β (SE)	P-value	CAF	β (SE)	P-value	CAF	β (SE)	P-value	CAF	β (SE)	P-value	
rs11206510	*PCSK9*	T	0.81	1.98 (0.45)	1.44E-05	0.86	0.09 (0.84)	0.91	0.93	−0.07 (1.30)	0.96	0.88	3.36 (1.44)	1.97E-02	N
rs11591147	*PCSK9*	T	0.02	−16.92 (1.42)	1.00E-32	4.10E-03	−22.64 (5.21)	1.41E-05	0.01	−15.66 (4.92)	1.44E-03	0.01	−23.39 (5.34)	1.19E-05	Y
rs646776	*CELSR2/PSCR1/SORT1*	A	0.78	5.74 (0.44)	1.44E-37	0.65	4.46 (0.63)	1.48E-12	–	–	–	0.81	7.70 (1.41)	4.49E-08	Y
rs599839	*CELSR2/PSRC1/SORT1*	A	0.77	5.67 (0.45)	3.61E-36	0.28	1.60 (0.72)	2.67E-02	0.78	6.17 (0.67)	3.94E-20	0.78	8.68 (1.75)	6.99E-07	Y
rs693	*APOB*	T	0.50	3.45 (0.36)	3.38E-21	0.24	1.60 (0.69)	2.04E-02	0.34	4.02 (0.59)	7.08E-12	0.38	1.38 (1.02)	0.18	N
rs562338	*APOB*	T	0.19	−5.52 (0.45)	1.05E-33	0.59	−2.54 (0.59)	1.57E-5	0.09	−5.44 (1.05)	1.93E-07	0.16	−3.90 (1.33)	3.42E-03	Y
rs754523	*APOB*	T	0.68	−3.64 (0.40)	3.44E-19	0.78	−2.12 (0.76)	5.52E-03	0.66	−4.26 (0.61)	2.17E-12	0.72	−1.63 (1.23)	0.19	N
rs6544713	*ABCG8*	T	0.31	2.98 (0.42)	1.17E-12	0.17	1.49 (0.74)	4.45E-02	0.11	4.76 (1.10)	1.51E-05	0.18	0.06 (1.22)	0.96	N
rs12654264	*HMGCR*	A	0.62	−2.66 (0.37)	6.56E-13	0.67	−2.02 (0.61)	9.39E-04	0.58	−1.17 (0.59)	4.55E-02	0.62	−2.06 (1.04)	4.68E-02	Y
rs1501908	*TIMD4*	G	0.64	1.23 (0.44)	4.961E-03	0.37	1.31 (0.64)	4.18E-02	0.85	−2.18 (0.89)	1.46E-02	0.76	2.40 (1.28)	6.19E-02	N
rs2650000	*HNF1A*	T	0.35	1.20 (0.39)	2.338E-03	0.12	0.15 (0.97)	0.88	0.41	0.73 (0.57)	0.20	0.37	2.58 (1.17)	2.84E-02	N
rs6511720	*LDLR*	T	0.12	−7.32 (0.52)	2.99E-44	0.13	−8.10 (0.80)	7.05E-24	0.07	−2.48 (1.41)	0.08	0.09	−6.43 (1.62)	7.34E-05	N
rs2228671	*LDLR*	T	0.12	−5.83 (0.97)	1.96E-09	0.04	−6.62 (2.94)	2.43E-02	–	–	–	0.08	−6.14 (2.03)	2.53E-03	Y
rs16996148	*CILP2/PBX4/NCAN1*	T	0.08	−2.88 (0.66)	1.40E-05	0.15	0.77 (0.80)	0.34	0.04	−0.70 (1.51)	0.64	0.06	−2.12 (2.01)	0.29	N
rs4803750	*BCL3*	A	0.93	5.57 (0.92)	1.37E-09	0.92	1.52 (1.77)	0.39	–	–	–	0.86	5.95 (4.68)	0.20	N
rs10402271	*APOE/C1/C4*	T	0.67	−2.27 (0.49)	3.86E-06	0.84	-1.38 (1.28)	0.28	–	–	–	0.61	2.39 (3.38)	0.48	N
rs4420638	*APOE/C1/C4*	A	0.82	−5.34 (0.51)	2.16E-25	0.79	0.16 (0.92)	0.87	0.90	−3.57 (1.07)	8.00E-04	0.90	−5.35 (1.72)	1.82E-03	N
rs2075650	*TOMM40*	A	0.88	−4.77 (1.23)	1.14E-04	0.87	−2.26 (2.40)	0.35	–	–	–	0.90	0.02 (5.27)	1.00	N
rs6102059	*MAFB*	T	0.30	−0.41 (0.52)	0.42	0.43	−0.77 (0.88)	0.38	–	–	–	0.29	−0.30 (1.22)	0.80	N

Coded allele (CA); coded allele frequency (CAF); beta coefficient (β); standard error (SE); data not available (–); generalized (G); yes (Y); no (N). Generalization is defined here as a significant association (p<0.05) and a similar direction of effect (β) compared with European Americans for the same test of association, across all racial/ethnic populations.

**Table 4:** Meta-analysis of GWAS–identified Triglyceride SNPs.

SNP	Nearest Gene	CA	European Americans(n_max_ = 24,258)			African Americans(n_max_ = 9,844)			American Indians(n_max_ = 6,157)			Mexican Americans and Hispanics(n_max_ = 2,973)			G
			CAF	β (SE)	P-value	CAF	β (SE)	P-value	CAF	β (SE)	P-value	CAF	β (SE)	P-value	
rs1748195	*ANGPTL3*	C	0.66	0.03 (0.01)	1.93E-07	0.35	0.01 (0.01)	0.19	0.61	0.16 (0.07)	2.44E-02	0.60	0.04 (0.01)	1.17E-02	N
rs1260326	*GCKR*	T	0.42	0.05 (0.01)	6.44E-13	0.16	0.05 (0.02)	9.98E-04	0.28	0.15 (0.09)	8.52E-02	0.33	0.06 (0.02)	1.97E-04	N
rs780094	*GCKR*	A	0.40	0.06 (0.01)	1.69E-32	0.18	0.02 (0.01)	2.91E-02	0.25	0.04 (0.01)	3.23E-03	0.33	0.06 (0.02)	1.13E-03	Y
rs17145738	*MLXIPL*	T	0.12	-0.07 (0.01)	5.71E-24	0.09	-0.03 (0.01)	2.53E-02	0.08	−0.07 (0.02)	2.30E-04	0.07	−0.09 (0.03)	7.40E-04	Y
rs328	*LPL*	C	0.90	0.09 (0.01)	4.16E-30	0.93	0.08 (0.02)	2.62E-08	0.97	0.09 (0.03)	4.83E-03	0.93	0.09 (0.03)	6.31E-04	Y
rs2197089	*LPL*	T	0.55	-0.03 (0.01)	4.97E-15	0.78	−0.01 (0.01)	7.45E-02	0.41	−0.05 (0.01)	2.57E-06	0.48	−0.05 (0.01)	4.01E-04	N
rs2954029	*TRIB1*	A	0.54	0.05 (0.01)	1.13E-04	0.68	-0.01 (0.02)	0.46	–	–	–	0.62	0.06 (0.02)	9.28E-04	N
rs174547	*FADS1*	T	0.66	−0.03 (0.01)	3.82E-10	0.91	−0.05 (0.01)	3.73E-04	0.21	−0.06 (0.02)	1.10E-04	0.39	−0.05 (0.02)	1.51E-03	Y
rs28927680	*APOA1/C3/A4/A5gene cluster*	C	0.93	−0.12 (0.01)	2.88E-38	0.84	<0.001 (0.01)	0.95	0.83	−0.13 (0.01)	6.33E-19	0.86	−0.08 (0.02)	2.15E-05	N
rs964184	*APOA1/C3/A4/A5gene cluster*	G	0.86	−0.14 (0.01)	1.91E-59	0.80	−0.02 (0.01)	4.87E-02	0.78	−0.17 (0.07)	1.43E-02	0.72	−0.14 (0.02)	1.04E-19	Y
rs3135506	*APOA1/C3/A4/A5gene cluster*	C	0.06	0.13 (0.01)	2.59E-33	0.06	0.11 (0.02)	2.06E-10	0.17	0.13 (0.01)	4.28E-20	0.14	0.13 (0.02)	3.08E-08	Y
rs4775041	*LIPC*	C	0.29	0.01 (0.01)	3.15E-02	0.14	0.03 (0.01)	4.29E-03	0.21	0.02 (0.01)	5.15E-02	0.18	0.01 (0.02)	0.58	N
rs16996148	*CILP2/PBX4/NCAN*	T	0.08	−0.04 (0.01)	3.91E-05	0.15	<0.001 (0.01)	0.77	0.04	−0.07 (0.03)	8.86E-03	0.06	−0.06 (0.03)	2.69E-02	N
rs7679	*PLTP*	T	0.82	−0.02 (0.01)	2.84E-02	0.96	−0.01 (0.02)	0.61	0.94	−2.0E-03 (0.02)	0.93	0.89	−0.03 (0.03)	0.31	N

Coded allele (CA); coded allele frequency (CAF); beta coefficient (β); standard error (SE); data not available (–); generalized (G); yes (Y); no (N). Generalization is defined here as a significant association (p<0.05) and a similar direction of effect (β) compared with European Americans for the same test of association, across all racial/ethnic populations.

A striking example of population differences in allele frequencies is *FADS1* rs174547. The T allele of *FADS1* rs174547 is the major allele in three populations (allele frequency  = 0.66, 0.91, and 0.59 in European Americans, African Americans, and Japanese/East Asians, respectively), but is the minor allele in the other three populations (allele frequency  = 0.39, 0.21, and 0.42 in Mexican Americans/Hispanics, American Indians, and Native Hawaiians/Pacific Islanders, respectively). Compared to European Americans, F_ST_ for this SNP was largest in American Indians (0.34) followed by African Americans (0.15).

We also compared allele frequencies between the various PAGE study sites, within each racial/ethnic group. As demonstrated in Figure S2, the allele frequencies of European Americans, African Americans, and Mexican Americans/Hispanics do not differ substantially across PAGE studies (allele frequencies differ by less than ±0.10). In contrast, over half of the SNPs genotyped in American Indians had allele frequency differences greater than ±0.10, with three SNPs with allele frequencies that differed by more than ±0.25. Comparisons are more difficult in Japanese/East Asians and Native Hawaiians/Pacific Islanders, as many SNPs were genotyped by only one PAGE study in these two racial/ethnic groups.

### Replication in European-descent populations

**Figure 1:**
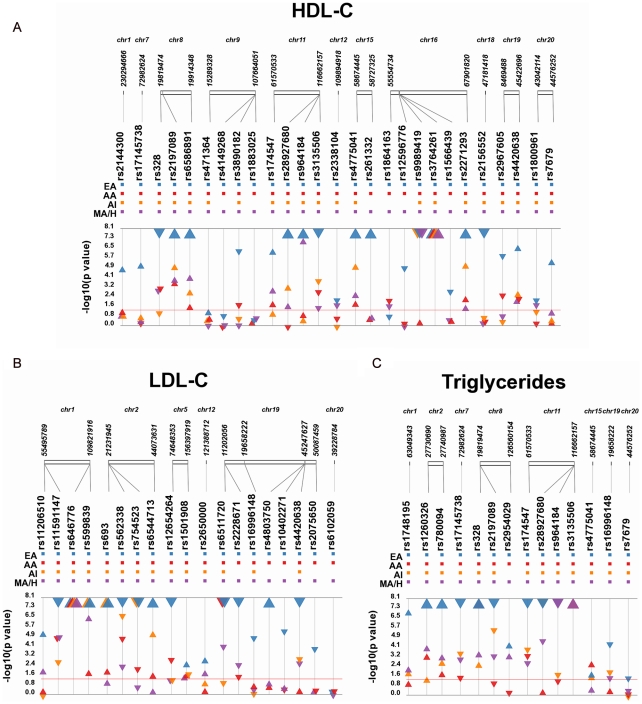
Meta-analysis results for GWAS–identified SNPs by population. Each SNP was tested for an association with the indicated trait assuming an additive genetic model adjusted for age and sex. Meta-analysis was performed, and p-values (−log_10_ transformed) of the meta-analysis are plotted along the y-axis using Synthesis-View [73], [74]. SNP location is given on the x-axis. Each triangle represents a meta-analysis p-value for each population. Populations are color-coded as follows: European Americans (blue; EA), African Americans (red; AA), Mexican Americans/Hispanics (orange; MA/H), and American Indians (purple; AI). Large triangles represent p-values at or smaller than genome-wide significance (p<10^−8^). The direction of the arrows corresponds to the direction of the beta coefficient. The significance threshold is indicated by the red bar at p = 0.05.

We meta-analyzed tests of association for 27, 19, and 14 SNPs previously associated with HDL-C, LDL-C, and/or triglycerides, respectively, across European American populations collected by individual PAGE study sites (Table S2). For HDL-C, 23 of the 27 (85%) SNPs tested were associated at p<0.05 assuming an additive genetic model and adjusting for age and sex (Figure 1 and Table 2). The four SNPs that did not replicate at this liberal significance threshold were rs471364 (*TTC39B*), rs1883025 (*ABCA1*), rs4149268 (*ABCA1*), and rs1864163 (*CETP*), all of which are intronic (Table S2). For LDL-C, only one (intergenic *MAFB* rs6102059) of the 19 SNPs tested was not significantly associated at p<0.05 (Figure 1 and Table 3). Finally, for ln(TG), all 14 SNPs tested were associated at p<0.05 (Figure 1 and Table 4).

Of the associations that did not replicate in the European-descent populations from PAGE, four out of five had sufficient power (>80%) to detect the previously reported effect size: *TTC39B* rs471364 (>99% power; HDL-C), *CETP* rs1864163 (80% power; HDL-C); *MAFB* rs6102059 (>90% power; LDL-C), and *ABCA1* rs4149268 (99% power; HDL-C). *ABCA1* rs1883025, which did not replicate the expected association with HDL-C, did not have sufficient power to detect the reported effect size (68% power; n = 3,865).

We then compared the genetic effect sizes reported in the literature to the genetic effect sizes estimated from the meta-analysis of these population-based studies. We observed that the majority of the point estimates of effect size (β) were smaller than previously reported estimates. Using the HDL-C association results as an example, 15 out of the 23 (65%) significant associations had effect estimates smaller than published effect estimates. We caution, however, that we did not formally test for significant differences between estimates and that these smaller effect estimates may or may not be significantly different than the published reports. However, it is interesting to note that 11 of our effect estimates differed from previous reports by more than 25%, including two HDL-C associations whose effect sizes differed by 50% or more from those in the literature (*ANGPTL4* rs2967605 and *MLXIPL* rs17145738; Table 2 and Table S2).

### Associations in non-European–descent populations

We meta-analyzed tests of association performed in African Americans for the same 27, 19, and 14 SNPs previously associated with HDL-C, LDL-C, and/or triglycerides in populations of European-descent. For all three traits studied, assuming an additive genetic model and adjusting for age and sex, approximately half of the tested GWAS-identified SNPs were associated at p<0.05: 12/27 (44%) for HDL-C, 11/19 (58%) for LDL-C, and 8/14 (57%) for ln(TG) (Figure 1, Figure S3, Table 2, Table 3, Table 4, Table 5). The majority of SNPs that failed to replicate in the meta-analysis for European Americans also failed to associate in the meta-analysis for African Americans. Interestingly, one SNP (*CETP* rs1864163) was significantly associated with HDL-C in African Americans (n = 451; CAF = 0.27; β = −2.79; p = 6.19×10^−3^) but not in European Americans (n = 291; CAF = 0.23; β = −2.07; p = 0.13).

**Table 5:** Observed versus expected number of significant associations, by trait and population.

Trait	Race/Ethnicity	# of Total Tests of Association*	# of Observed Significant Associations	# of Expected Significant Associations†	P-value‡
**HDL-C**	AA	23	11	17.3	0.01
	AI	20	9	14.4	0.01
	MA/H	23	13	13.8	0.83
**LDL-C**	AA	18	11	14.7	0.03
	AI	14	10	11.9	0.15
	MA/H	18	10	10.6	0.81
**ln(TG)**	AA	14	8	11.9	0.01
	AI	13	10	8.4	0.56
	MA/H	14	12	10.4	0.54

The expected number of significant associations was based on power calculations assuming an additive genetic model and liberal significance threshold (0.05) in each racial/ethnic group for each test of association. We further assumed the observed genetic effect size (beta) from PAGE European Americans and the observed allele frequency, sample sizes, and trait mean/standard deviations from each non-European American population.

*Only includes associations that replicated in EA.

†Based on the additive power of all loci.

‡One-sample binomial test.

African Americans (AA); American Indians (AI); Mexican Americans/Hispanics (MA/H); number (#).

Other populations that were examined for select SNPs included American Indians, Mexican Americans/Hispanics, Japanese/East Asians, and Native Hawaiians/Pacific Islanders. Among American Indians, 9/21 (43%), 10/14 (71%), and 10/13 (77%) of the SNPs tested for association with HDL-C, LDL-C, and ln(TG), respectively, were associated at the liberal significance threshold of p<0.05. For Mexican Americans/Hispanics, 14/27 (52%), 10/19 (53%), and 12/14 (86%) SNPs were significantly associated at p<0.05 with HDL-C, LDL-C, and ln(TG), respectively. Despite a small sample size, intronic *CETP* rs1864163 was significantly associated with HDL-C in Mexican Americans/Hispanics (n = 265; CAF = 0.28; β = −2.98; p = 1.78×10^−2^) but not in European Americans (n = 291; CAF = 0.27; β = −2.07; p = 0.13), although the size and the direction of effect were similar. Venn diagrams representing the overlap of significant associations across the four major PAGE populations are presented in Figure S3.

The sample sizes for Japanese/East Asians and Native Hawaiians/Pacific Islanders are considerably smaller compared with the other populations examined. Despite the lower power to detect associations, significant associations were observed for both groups at a liberal significance threshold of p<0.05. Among the 26, 18, and 13 SNPs tested for associations with HDL-C, LDL-C, and ln(TG), respectively, there were nine (35%), three (17%), and three (23%) SNPs significantly associated in the combined Japanese/East Asian group.

For Native Hawaiians/Pacific Islanders, the group with the smallest sample size considered here, one SNP each was associated with HDL-C (*APOA1/C3/A4/A5* gene cluster rs28927680) and LDL-C (*APOB* rs754523) out of the 24 and 18 SNPs tested for association, respectively. Three out of 12 SNPs tested for an association with ln(TG) were associated at p<0.05 (*PLTP* rs7679, *MLXIPL* rs17145738, and *APOA1/C3/A4/A5* gene cluster rs28927680), with the latter at a significance of p<10^−19^.

### Generalization across non-European–descent populations

For the 55 SNP-trait associations that replicated in European Americans, we determined which associations generalized across all four of our largest populations (European Americans, African Americans, American Indians, and Mexican Americans/Hispanics). Generalization was based on two criteria: 1) level of significance (i.e. p-value) and 2) direction of effect (i.e. positive or negative beta). SNPs that were significantly associated at p<0.05 and had the same direction of effect as European Americans in all populations studied were considered to have generalized. For HDL-C, five SNPs (*CETP* rs3764261, *LPL* rs6586891, *LIPC* rs4775041, *LPL* rs2197089, and *APOA1/C3/A4/A5* gene cluster rs3135506) met these criteria, and two SNPs (*LCAT* rs2271293 and *LPL* rs328) were associated in three groups and trended towards significance in a fourth group (p = 0.06 and p = 0.07 in Mexican Americans/Hispanics and American Indians, respectively; Table 2).

For LDL-C, six SNPs generalized across all four groups, if genotyped: *APOB* rs562338, *CELSR2/PSRC1/SORT1* rs599839 and rs646776, *PCSK9* rs11591147, *HMGCR* rs12654264, and *LDLR* rs2228671 (Table 3). Similarly for ln(TG), six SNPs were significantly associated across the four largest populations: *APOA1/C3/A4/A5* gene cluster rs964184 and rs3135506, *GCKR* rs780094, *LPL* rs328, *MLXIPL* rs1714573, and *FADS1* rs174547. In addition, for ln(TG), two SNPs (*LPL* rs2197089 and *GCKR* rs1260326) were associated in three groups and trended towards significance in a fourth group (p = 0.07 in African Americans and p = 0.09 in American Indians, respectively). Among the 17 SNPs that generalized across the largest groups among the three lipid traits, only four (24%) were either nonsense (rs328) or missense SNPs (rs3135506, rs11591147, and rs1260326; Table S2).

### Power

Based on our definition of generalization, several SNPs discovered and replicated in European-descent populations failed to generalize to other populations. There are several possible explanations for non-generalization, including power. To further investigate potential lack of power, we first performed post-hoc power calculations assuming an additive genetic model and liberal significance threshold (0.05) in each racial/ethnic group for each test of association. In these power calculations, we further assumed the observed genetic effect size (beta) from PAGE European Americans and the observed allele frequency, sample sizes, and trait mean/standard deviations from each non-European American population. By adding the power of all tested loci, we estimated the number of expected significant associations and compared this to the number of observed significant associations (Table 5).

In general, the number of expected significant associations was greater than the number observed. African Americans consistently had fewer significant associations (11, 11, and 8 for HDL-C, LDL-C, and ln(TG), respectively) than expected (17.3, 14.7, and 11.9 for HDL-C, LDL-C, and ln(TG), respectively) based on power, regardless of the lipid trait being tested. More specifically, we were powered to detect in African Americans 17 of the 25 associations that replicated in European Americans but failed to generalize to African Americans.

Compared to African Americans, differences between the observed and the expected number of associations for American Indians and Mexican Americans/Hispanics were less extreme. In fact, for ln(TG), *more* significant associations were detected in these two populations than the PAGE study was powered to detect (8.4 and 10.4 expected; 10 and 12 observed for American Indians and Mexican Americans/Hispanics, respectively; Table 5). We were powered to detect in American Indians nine of the 18 associations that replicated in European Americans but did not generalize to American Indians. Similarly, we were powered to detect in Mexican Americans/Hispanics eight of the 20 associations that replicated in European Americans but failed to generalize to Mexican Americans/Hispanics.

### Linkage disequilibrium

To examine whether LD can account for the lack of generalization of the properly powered tests of association in African Americans, we examined LD patterns in HapMap Europeans (CEU) and West Africans (YRI) as well as those published in the literature for the genotyped SNPs and surrounding variation. For *APOA1/C3/A4/A*5 rs28927680, previous studies in European-descent populations have noted that this SNP is in strong LD (r^2^ = 0.98) with missense *APOA5* rs3135506 [42]. *APOA1/C3/A4/A5* rs964184 is also in moderate LD with missense rs3135506 (r^2^ = 0.510 in CEU). However, neither rs28927680 nor rs964184 are in LD with missense rs3135506 (r^2^ = 0.039 and r^2^ = 0.048) in YRI. Furthermore, *APOA5* rs3135506 is associated with HDL-C in European Americans, African Americans, Mexican Americans/Hispanics, and American Indians (Table 1 and Table 2). Generalization of rs3135506 coupled with non-generalization and differences in YRI LD patterns for rs28927680 and rs964184 suggest that *APOA5* rs3135506 is either the putative functional SNP for the association with HDL-C or in LD with the functional SNP. Although the exact mechanism is not yet known, molecular modeling [53] as well as *in vitro* [53] and *in vivo* [54], [55] studies support the epidemiologic evidence that rs3135506 is functional.

Other interpretations of LD patterns are more difficult. For example, *CETP* rs9989419, which failed to generalize in African Americans for HDL-C despite sufficient power, is not in strong LD with obvious functional SNPs in CEU within 50 kb flanking the genotyped SNP. The strongest pair-wise LD (r^2^ = 0.251) consists of intergenic and intronic SNPs, and these same SNPs have weak LD (r^2^<0.03) or are not found in YRI. Similarly, *LIPC* rs261332 associated with HDL-C levels in European Americans but failed to generalize in African Americans. *LIPC* rs261332 is in strong LD (r^2^>0.80 in CEU) with SNPs in the 5′ flanking region of *LIPC*, but not in LD with these same SNPs in YRI (r^2^<0.15).

### Adjustments for exposures and co-morbidities

Genetic variations in isolation are not the sole determinants of lipid trait distributions. Many environmental exposures and demographic variables are associated with lipid traits. To account for these variables, we meta-analyzed all tests of association for HDL-C, LDL-C, and ln(TG) adjusted for age, sex, body mass index, current smoking, type 2 diabetes, post-menopausal status, and current hormone use. Adjustment for these additional covariates did not appreciably alter the results compared with the models minimally adjusted for age and sex (Figures S4, S5, S6). Inclusion of previous myocardial infarction as a variable to the fully adjusted model also did not appreciably alter the results compared with the minimally adjusted models (Figures S4, S5, S6).

### Effect of including versus excluding by medication use

All analyses presented thus far include fasting adult participants regardless of lipid lowering medication use. Many GWAS conducted for the lipid traits excluded participants on lipid lowering medication [40], [42], [43] given that these medications substantially lower LDL-C levels. We have included these participants for analysis as participants on lipid lowering medication could represent the upper extreme of the normal LDL-C distribution associated with a genetic profile found in a general population. Exclusion of these participants would preclude these meta-analyses from fully describing the extent and strength of associations relevant to these traits in a population-based setting. However, if genetic variation is associated with lipid concentrations and medication use lowers lipid concentrations, inclusion of participants on lipid lowering medications could bias associations towards the null. As a sensitivity analysis, WHI used detailed medication data available on a subset of participants, and performed the tests of association for HDL-C, LDL-C, and ln(TG) excluding and including participants on lipid lowering medication with the latter adjusted for medication usage using average effects estimated in Wu et al [56] for specific drug classes. Figure S7 suggests that both the point estimates and the confidence intervals of the genetic effects are similar for this female-only study whether participants are excluded or included and adjusted for medication use.

We also performed a second sensitivity analysis: tests of association excluding participants on lipid lowering medication for all models. As detailed in Figures S8, S9, S10, excluding participants on lipid lowering medication usage does not appreciably alter the results, with the possible exception of LDL-C associations in Japanese/East Asians. More specifically, two SNPs (rs11206510 and rs1501908) became significantly associated with LDL-C after excluding participants on medications while two other SNPs (rs562338 and rs6544713) were no longer significantly associated (Figure S9). The difference in significance for these four tests of association may be related to lipid lowering medication use; however, it is more likely due to statistical fluctuations from small samples sizes (n_Include_ = 690; n_Exclude_ = 467). Also of note, use of lipid-lowering medications was low (<10%) in the ARIC, CHS, NHANES, and WHI studies since the majority of study recruitment occurred before the introduction or widespread use of the recent generation of lipid-lowering medications. Medication use was higher in the MEC study (20–38% depending on the population), which contributed the majority of Japanese/East Asian samples.

## Discussion

We have performed an extensive replication and generalization effort for HDL-C, LDL-C, and TG GWAS-identified SNPs. The PAGE study consists of six racial/ethnic groups: European American, African American, Mexican American/Hispanic, American Indian, Japanese/East Asian, and Native Hawaiian/Pacific Islander, with population-specific sample sizes ranging from ∼100 to >20,000 for any one test of association. Although power to detect associations varied across the lipid traits and populations, we observed general patterns worth noting for future genetic epidemiological studies.

### Replication in European-descent populations

Perhaps not unexpectedly, we were able to replicate most reported associations in European Americans. Regardless of significance, all but one of the tested SNPs had effect estimates in the same direction as the previously reported association from the literature. *FADS1* rs174547, which was significantly associated with decreased ln(TG) in this meta-analysis for European Americans, was associated with increased TG in European Americans from the Framingham Heart Study (n = 7,423) [43]. HDL-C had proportionally (15%) the greatest number of SNPs that failed to replicate in European Americans compared with LDL-C (5%) and TG (0%) despite the fact that we had sufficient power to detect the reported genetic effect size for many of these tests. *TTC39B* rs471364 was not associated with HDL-C levels despite a sample size of 18,089 and >99% power to detect the reported effect size. Neither *ABCA1* rs4149268 nor rs1883025 was associated with HDL-C, although the latter test of association was underpowered (68%; n = 3,865). Finally, as previously discussed, *CETP* rs1864163 was not associated with HDL-C in this European American dataset although we had 80% power to detect the reported genetic effect size. For LDL-C, only *MAFB* rs6102059 was not associated despite >90% power to detect the reported effect size.

The reasons for non-replication in this European American dataset for properly powered tests of association are unclear. It is possible that we have overestimated our power to detect reported associations. The “winner's curse” and inflated genetic effect estimates from initial discovery are well known [57], [58]. Indeed, for the five SNPs that did not replicate in this meta-analysis for European Americans, the association was described in only one GWAS each despite the fact that numerous GWAS [31], [33]–[43] and a large meta-analysis [32] for these three traits have been conducted in populations of European-descent. The meta-analysis recently reported by Teslovich et al [32] did report significant associations between *TTC39B* rs581080 for HDL-C and *MAFB* rs2902940 for LDL-C. *TTC39B* rs581080 is in moderate linkage disequilibrium (LD) with rs471364 (r^2^ = 0.49 in CEU HapMap), but *MAFB* rs2902940 is not in LD with rs6102059 (r^2^ = 0.03 in HapMap CEU).

A second possibility for our observed non-replication is heterogeneity among the PAGE studies. Because it is important to understand the degree to which associations are consistent across individual studies, we compared directions of effect (betas) across PAGE study sites for each test of association (Figures S11, S12, S13) and performed tests of heterogeneity. Association results for *TTC39B* rs471364, which meta-analysis result for HDL-C in European Americans was insignificant, had significant evidence for heterogeneity across studies (p_heterogeneity_ = 0.048; I^2^ = 58.25%). In four of the five PAGE study sites, the association between this SNP and HDL-C had consistent directions of effect; however, only one test of association was significant in European Americans (p = 0.005 in EAGLE; Figure S11). Only two other association results had evidence for heterogeneity among European Americans: *FADS1* rs174547 for HDL-C (p_heterogeneity_ = 0.006; I^2^ = 75.73%) and *PCSK9* rs11206510 for LDL-C (p_heterogeneity_ = 0.048; I^2^ = 55.34%). However, for both of these loci, the tests of association were significant in European Americans and had similar directions of effect in all but one of the PAGE study sites (Figures S11 and S12).

### Generalization to non-European populations

When taking into account power, significance, and direction of effect, most SNPs discovered in European Americans generalized to African Americans, Mexican Americans, and American Indians. Of note are the eleven tests of association significant in European Americans that did not generalize to African Americans despite having adequate power. Given that GWAS products are a mixture of tagSNPs and functional SNPs, it is likely that discovery in European Americans represents tagSNPs rather than the true functional SNP. Because linkage disequilibrium patterns differ across populations, tagSNPs genotyped directly in populations of non-European descent may not recapitulate the association observed in European-descent populations depending on the pattern of LD. The association of HDL-C and nonsynonymous rs3135506 versus tagSNPs rs28927680 in the *APOA1/C3/A4/A5*gene cluster in this analysis is an example of the effects of LD and the ability to generalize across populations.

Evoking LD as an explanation for lack of generalization is appealing, but it does have limitations given that the functional SNP is not often obvious. All tests of association that did not generalize to African Americans had evidence of LD differences between CEU and YRI using the HapMap data. However, most of these SNPs are located in the intergenic and intronic regions. Further fine-mapping in both the discovery population as well as other diverse populations will be needed along with a better understanding of genetic variation and its relationship to biological function to identify the true functional SNPs for these traits.

Among the five putative functional SNPs genotyped (nonsynonymous rs11591147, rs1260326, rs3135506, and rs1800961 and nonsense rs328), all five replicated in populations of European-descent, and three of the five generalized to populations of non-European descent. One putative functional SNP that did not replicate across populations was *HNF4A* rs1800961, likely due to low power because of the very low minor allele frequency in all subpopulations (0.0065 to 0.0398). Both the direction and magnitude of effect, however, were consistent across groups. *GCKR* rs1260326 did not generalize to all populations of non-European descent but did generalize in three of the four populations tested and trended towards significance in American Indians (p = 0.085; Table 4).

### Limitations and strengths

The major strengths and limitations of the PAGE study for lipids are sample size and diversity. The largest sample size is for samples of European-descent (∼20,000), followed by African Americans and American Indians. The sample sizes for Mexican Americans, Japanese/East Asians, and Pacific Islanders/Native Hawaiians are smaller and consequently underpowered for tests of association as estimated from genetic effect sizes in the published European-descent discovery studies. Also, not all SNPs were genotyped in all PAGE studies, further affecting the power of the meta-analyses.

An additional limitation is the lack of data related to lipid lowering medication. Ideally, all analyses would be adjusted for use of lipid lowering medication based on the type and dose of medication. In most PAGE studies, these data were not available and in many, use was low at baseline when blood samples were obtained. As we demonstrate in Supplementary material, inclusion of participants using lipid-lowering medication did not appreciably alter the results of the meta-analysis when compared with excluding these participants. While this finding may be useful for future studies, we caution that the majority of participants in this study were not on lipid lowering medications.

In general, the cohorts and surveys included in PAGE are diverse with regard to demographics, genetic ancestry, lifestyle, health, and environmental exposure. Despite this diversity, very few tests of association from the meta-analysis exhibited evidence of heterogeneity.

### Conclusions

Overall, the majority of GWAS-identified SNPs for HDL-C, LDL-C, and TG replicated in European Americans and generalized to non-European-descent populations. These results suggest that the genotyped SNP either tags the functional SNP(s) common across these populations or that the genotyped SNP represents the risk SNP directly. SNPs that replicated in European Americans but did not generalize in the largest non-European-descent populations, despite adequate power, could represent priority associations that require fine-mapping and re-sequencing to identify the functional variant(s).

## Materials and Methods

### Study populations and phenotypes

All studies were approved by Institutional Review Boards at their respective sites (details are given in Text S1). PAGE study samples were drawn from four large population-based studies or consortia: EAGLE (Epidemiologic Architecture for Genes Linked to Environment), based on three National Health and Nutrition Examination Surveys (NHANES) [59]–[61], the Multiethnic Cohort (MEC) [62], the Women's Health Initiative (WHI) [63], [64], and Causal Variants Across the Life Course (CALiCo), a consortium of several cohort studies: Atherosclerosis Risk in Communities Study (ARIC) [65], Coronary Artery Risk in Young Adults (CARDIA) [66], Cardiovascular Health Study (CHS) [67], Strong Heart Family Study (SHFS) [68], and Strong Heart Cohort Study (SHS) [69] (Table 1). The PAGE study design is detailed in Matise et al [30].

Serum HDL-C, triglycerides, and total cholesterol were measured using standard enzymatic methods. LDL-C was calculated using the Friedewald equation [30], [70], with missing values assigned for samples with triglyceride levels greater than 400 mg/dl. For PAGE study sites with longitudinal data, the baseline measurement was used for analysis. A full description of each study, along with population-specific study characteristics, is presented in Text S1 and Table S1.

### SNP selection and genotyping

All SNPs considered for genotyping were previously associated with HDL-C, LDL-C, and/or triglycerides in published (as of 2008) candidate gene and genome-wide association studies. A total of 52 SNPs were targeted for genotyping by two or more PAGE study sites. There is no overlap between samples used in this study and samples used in GWAS from which the SNPs were selected. The 52 targeted variants are located in or nearby 32 different genes/gene regions, with 12 of the gene/gene regions represented by two or more SNPs. Five SNPs are nonsynonymous, one SNP is a nonsense variant, and two SNPs are synonymous; the remainder are located in introns, flanking, or intergenic regions. The full list of targeted SNPs, their locations, and their previously associated lipid trait can be found in Table S2.

Cohorts and surveys were genotyped using either commercially available genotyping arrays (Affymetrix 6.0, Illumina 370CNV BeadChip), custom mid- and low-throughput assays (TaqMan, Sequenom, Illumina GoldenGate or BeadXpress), or a combination thereof. Quality control was implemented at each study site independently. In addition to site-specific quality control, all PAGE study sites genotyped 360 DNA samples from the International HapMap Project and submitted these data to the PAGE Coordinating Center for concordance statistics [71]. Study specific genotyping details are described in Text S1. Of the 52 targeted SNPs, three (*CETP* rs1800775, *APOE* rs429358, and *APOE* rs7412) failed at all PAGE study sites that attempted genotyping; therefore, a total of 49 SNPs were tested in this analysis.

### Statistical methods

All tests of association were performed by each PAGE study site using the same analysis protocol prior to meta-analysis. The study protocol excluded participants <18 years of age as well as non-fasting samples (defined here as <8 hours). When triglyceride level was the dependent variable, participants with >1,000 mg/dl were excluded from analyses. Triglyceride (TG) levels were natural-log transformed (ln) prior to analysis.

Linear regression was performed for fasting adults regardless of lipid lowering medication use with HDL-C, LDL-C, or ln(TG) as the dependent variable and a SNP as the independent variable, assuming an additive genetic model, stratified by race/ethnicity. The coded allele is reported in Table 2, Table 3, Table 4. The beta estimate is per additional copy of the coded allele. For each SNP, four models were considered: 1) unadjusted, 2) adjusted for age (continuous in years) and sex, 3) adjusted for age, body mass index (continuous in kg/m^2^), current smoking (yes/no; binary), type 2 diabetes (yes/no; binary), post-menopausal status (yes/no for females only; binary), and current hormone use (yes/no for females only; binary), and 4) adjusted for age, body mass index, current smoking, type 2 diabetes, post-menopausal status, current hormone use, and previous myocardial infarction (yes/no; binary). All PAGE study sites (except for WHI, which is female only) stratified models 3 and 4 by sex given the sex-specific variables (post-menopausal status and hormone use) prior to meta-analysis. Select PAGE study sites also included study site or site of ascertainment as a covariate in all models. Results from Model 2 (adjusted for age and sex) are reported in the main text while results from Models 1, 3, and 4 are presented in Figures S4, S5, S6. Model 2 excluding participants on lipid-lowering medications are presented in Figures S8, S9, S10.

Meta-analyses, using a fixed-effects inverse-variance weighted approach and tests for effect size heterogeneity across studies, were performed using METAL [72]. P-values were not adjusted for multiple testing, and association results were plotted using Synthesis-View [73], [74], where indicated. Power calculations were performed using Quanto [75], [76] assuming unrelated participants, an additive genetic model, the published effect size from European-descent populations listed in Table S1, and the population-specific allele frequencies listed in Table 2, Table 3, Table 4. Linkage disequilibrium was calculated using HapMap European (CEU) and West African (YRI) data accessed through the Genome Variation Server. F_ST_ was calculated using the Weir and Cockerham algorithm [77]. Aggregate data from the meta-analysis as well as individual tests of association from each PAGE study site will be made available via dbGaP [30], [78].

### Web resources

NHGRI GWAS Catalog (www.genome.gov/GWAStudies).

Genome Variation Server (pga.gs.washington.edu).

Synthesis-View (http://chgr.mc.vanderbilt.edu/ritchielab/method.php?method=synthesisview).

## Supporting Information

Figure S1 Coded allele frequency, by population. The coded allele frequency (CAF) is plotted for each of the 49 SNPs by population using Synthesis-View [73], [74]. The populations include European Americans (EA), African Americans (AA), Mexican Americans/Hispanics (MA/H), American Indians (AI), Japanese/East Asians (J/EA), and Native Hawaiians/Pacific Islanders (NH/PI).
(DOCX)

Figure S2 Coded allele frequency across PAGE study sites, by population. The coded allele frequency (CAF) is plotted for each of the 49 SNPs by population using Synthesis-View [73], [74]. The studies include: Atherosclerosis Risk in Communities (ARIC), Coronary Artery Risk in Young Adults (CARDIA), Cardiovascular Heart Study (CHS), Epidemiologic Architecture for Genes Linked to Environment (EAGLE), Multiethnic Cohort (MEC), Women's Health Initiative (WHI), Strong Heart Community Study (SHCS), and Strong Heart Family Study (SHFS) in Arizona (AZ), Oklahoma (OK) and South Dakota (SD).
(DOCX)

Figure S3 Venn diagrams representing the overlap of significant associations (p<0.05) across the four major PAGE populations (European Americans, African Americans, Native Americans, and Mexican Americans/Hispanics, for the three lipid traits (HDL-C, LDL-C, and TG).
(DOCX)

Figure S4 Comparison of unadjusted, minimally adjusted, adjusted models for HDL-C, by population. Results of tests of association for four regression models are plotted: model 1 (unadjusted), model 2 (adjusted for age and sex; and site of ascertainment for select PAGE studies), model 3 (adjusted for age, sex, body mass index, current smoking, type 2 diabetes, post-menopausal status, and current hormone use), and model 4 (model 3 with the addition of previous myocardial infarction). Each SNP was tested for an association with HDL-C. Meta-analysis was performed, and p-values (−log_10_ transformed) of the meta-analysis are plotted along the y-axis. SNP location is given on the x-axis. Each triangle represents a meta-analysis p-value for each population. Models are color coded. Large triangles represent p-values at or smaller than genome-wide significance (p<10^−8^). The direction of the arrows corresponds to the direction of the beta coefficient. The exact beta coefficients are reported on the bottom panel. The significance threshold is indicated by the red bar at p = 0.05.
(DOCX)

Figure S5 Comparison of unadjusted, minimally adjusted, adjusted models for LDL-C, by population. Results of tests of association for four regression models are plotted: model 1 (unadjusted), model 2 (adjusted for age and sex; and site of ascertainment for select PAGE studies), model 3 (adjusted for age, sex, body mass index, current smoking, type 2 diabetes, post-menopausal status, and current hormone use), and model 4 (model 3 with the addition of previous myocardial infarction). Each SNP was tested for an association with LDL-C. Meta-analysis was performed, and p-values (−log_10_ transformed) of the meta-analysis are plotted along the y-axis. SNP location is given on the x-axis. Each triangle represents a meta-analysis p-value for each population. Models are color coded. Large triangles represent p-values at or smaller than genome-wide significance (p<10^−8^). The direction of the arrows corresponds to the direction of the beta coefficient. The exact beta coefficients are reported on the bottom panel. The significance threshold is indicated by the red bar at p = 0.05.
(DOCX)

Figure S6 Comparison of unadjusted, minimally adjusted, adjusted models for triglyceride concentrations, by population. Results of tests of association for four regression models are plotted: model 1 (unadjusted), model 2 (adjusted for age and sex; and site of ascertainment for select PAGE studies), model 3 (adjusted for age, sex, body mass index, current smoking, type 2 diabetes, post-menopausal status, and current hormone use), and model 4 (model 3 with the addition of previous myocardial infarction). Each SNP was tested for an association with triglycerides. Meta-analysis was performed, and p-values (–log_10_ transformed) of the meta-analysis are plotted along the y-axis. SNP location is given on the x-axis. Each triangle represents a meta-analysis p-value for each population. Models are color coded. Large triangles represent p-values at or smaller than genome-wide significance (p<10^−8^). The direction of the arrows corresponds to the direction of the beta coefficient. The exact beta coefficients are reported on the bottom panel. The significance threshold is indicated by the red bar at p = 0.05.
(DOCX)

Figure S7 Comparison of genetic effect estimates when participants are excluded or included based on medication use with adjustments in WHI. Genetic effect estimates (β) and 95% confidence interval are plotted for each SNP tested for an association. The tests of association were performed on fasting European Americans adjusted for age and sex and excluding participants on lipid lowering medication (blue), including all participants regardless of medication use (green), and all participants on lipid lowering medication, adjusted for the average HDL-C, LDL-C, and ln(TG) effects estimated by Wu et al [87].
(DOCX)

Figure S8 HDL-C and the effects of lipid lowering medication use on genetic associations, by population. Comparison of genetic effects and significance when tests of association are performed within fasting adults regardless of lipid lowering medication (Include) versus fasting adults not on lipid lowering medication (Exclude). All tests of association results shown here are minimally adjusted for age and sex.
(DOCX)

Figure S9 LDL-C and the effects of lipid lowering medication use on genetic associations, by population. Comparison of genetic effects and significance when tests of association are performed within fasting adults regardless of lipid lowering medication versus fasting adults not on lipid lowering medication. All tests of association results shown here are minimally adjusted for age and sex.
(DOCX)

Figure S10 Transformed triglycerides and the effects of lipid lowering medication use on genetic associations, by population. Comparison of genetic effects and significance when tests of association are performed within fasting adults regardless of lipid lowering medication versus fasting adults not on lipid lowering medication. All tests of association results shown here are minimally adjusted for age and sex.
(DOCX)

Figure S11 Comparison of HDL-C associations across PAGE study sites, by population. Results of tests of association for the various PAGE study sites are plotted (where available) along with meta-analysis results (META): Atherosclerosis Risk in Communities (ARIC), Coronary Artery Risk in Young Adults (CARDIA), Cardiovascular Heart Study (CHS), Epidemiologic Architecture for Genes Linked to Environment (EAGLE), Multiethnic Cohort (MEC), Women's Health Initiative (WHI), Strong Heart Community Study (SHCS), and Strong Heart Family Study (SHFS) in Arizona(AZ), Oklahoma (OK) and South Dakota (SD). Each SNP was tested for an association with HDL-C, adjusted for age and sex (Model 2), including fasting adults on lipid lowering medications. SNP location is given on the x-axis and p-values (−log_10_ transformed) are plotted along the y-axis. Each triangle represents a p-value for each PAGE study. PAGE study sites are color coded. Large triangles represent p-values at or smaller than genome-wide significance (p<10^−8^). The direction of the arrows corresponds to the direction of the beta coefficient. The exact beta coefficients are reported on the bottom panel. The significance threshold is indicated by the red bar at p = 0.05.
(DOCX)

Figure S12 Comparison of LDL-C associations across PAGE study sites, by population. Results of tests of association for the various PAGE study sites are plotted (where available) along with meta-analysis results (META): Atherosclerosis Risk in Communities (ARIC), Coronary Artery Risk in Young Adults (CARDIA), Cardiovascular Heart Study (CHS), Epidemiologic Architecture for Genes Linked to Environment (EAGLE), Multiethnic Cohort (MEC), Women's Health Initiative (WHI), Strong Heart Community Study (SHCS), and Strong Heart Family Study (SHFS) in Arizona(AZ), Oklahoma (OK) and South Dakota (SD). Each SNP was tested for an association with LDL-C levels, adjusted for age and sex (Model 2), including fasting adults on lipid lowering medications. SNP location is given on the x-axis and p-values (−log_10_ transformed) are plotted along the y-axis. Each triangle represents a p-value for each PAGE study. PAGE study sites are color coded. Large triangles represent p-values at or smaller than genome-wide significance (p<10^−8^). The direction of the arrows corresponds to the direction of the beta coefficient. The exact beta coefficients are reported on the bottom panel. The significance threshold is indicated by the red bar at p = 0.05.
(DOCX)

Figure S13 Comparison transformed triglyceride associations across PAGE study sites, by population. Results of tests of association for the various PAGE study sites are plotted (where available) along with meta-analysis results (META): Atherosclerosis Risk in Communities (ARIC), Coronary Artery Risk in Young Adults (CARDIA), Cardiovascular Heart Study (CHS), Epidemiologic Architecture for Genes Linked to Environment (EAGLE), Multiethnic Cohort (MEC), Women's Health Initiative (WHI), Strong Heart Community Study (SHCS), and Strong Heart Family Study (SHFS) in Arizona(AZ), Oklahoma (OK) and South Dakota (SD). Each SNP was tested for an association with natural-log transformed triglyceride levels, adjusted for age and sex (Model 2), including fasting adults on lipid lowering medications. SNP location is given on the x-axis and p-values (-log_10_ transformed) are plotted along the y-axis. Each triangle represents a p-value for each PAGE study. PAGE study sites are color coded. Large triangles represent p-values at or smaller than genome-wide significance (p<10^−8^). The direction of the arrows corresponds to the direction of the beta coefficient. The exact beta coefficients are reported on the bottom panel. The significance threshold is indicated by the red bar at p = 0.05.
(DOCX)

Table S1 Study characteristics by PAGE study and population. Descriptive statistics for fasting (≥8 hours) adults (≥18 years of age) are expressed as percentage, median, and standard deviation (SD) for each variable.
(DOCX)

Table S2 List of candidate gene and GWAS-identified SNPs targeted for genotyping in PAGE. For each SNP (denoted by rs number), we list the chromosomal and genomic location, the putative function of the SNP (based on SNP location) and the nearest gene, the number of PAGE studies that genotyped the SNP, the trait associated with the SNP based on the literature, the effect allele and effect size based on the literature, and the reference for these data.
(DOC)

Text S1 Study descriptions.
(DOCX)
