# Supplementary figures and images for: Genetic Determinants of Lipid Traits in Diverse Populations from the Population Architecture using Genomics and Epidemiology (PAGE) Study
Source: PLoS Genet. 2011 Jun 30;7(6):e1002138. doi: 10.1371/journal.pgen.1002138 (PMC3128106; doi:10.1371/journal.pgen.1002138)

**Figure S1. Coded allele frequency, by population.**


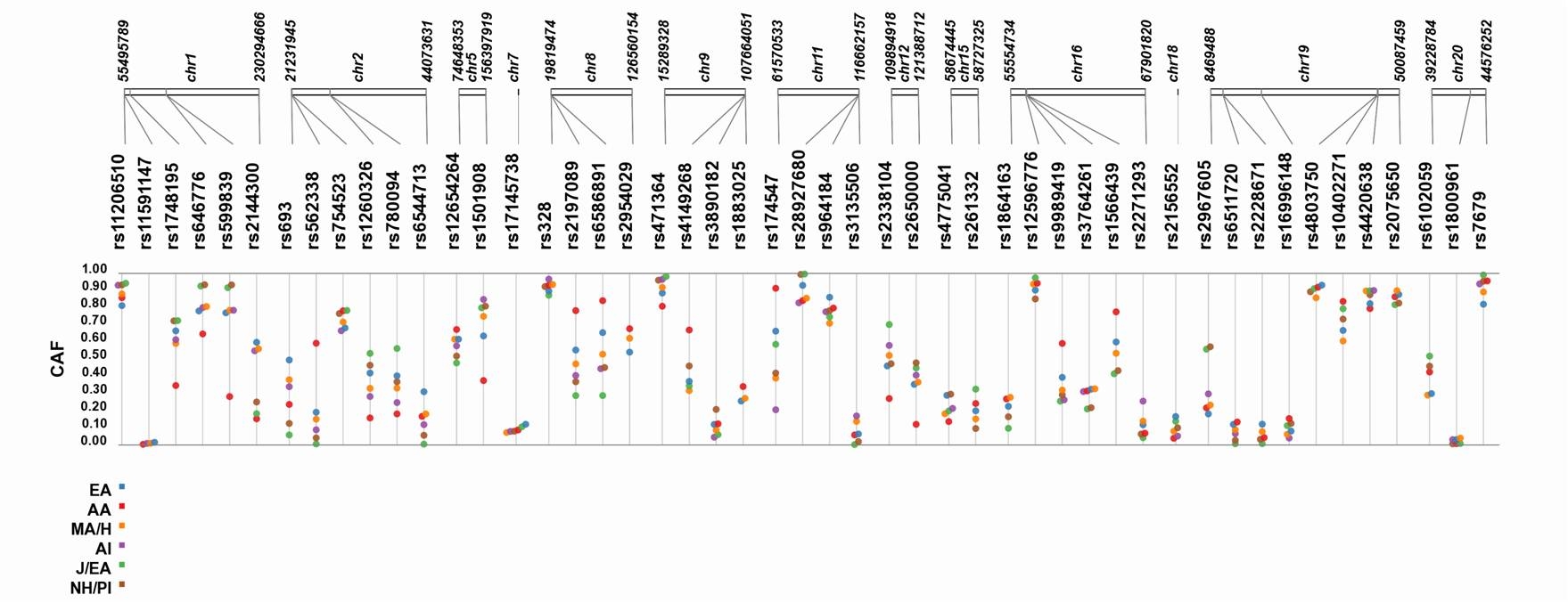

Supplement: Figure S1 — Coded allele frequency, by population. The coded allele frequency (CAF) is plotted for each of the 49 SNPs by population using Synthesis-View [73], [74]. The populations include European Americans (EA), African Americans (AA), Mexican Americans/Hispanics (MA/H), American Indians (AI), Japanese/East Asians (J/EA), and Native Hawaiians/Pacific Islanders (NH/PI). (DOCX) [file pgen.1002138.s001.docx]
