# Supplementary material for: Genetic Determinants of Lipid Traits in Diverse Populations from the Population Architecture using Genomics and Epidemiology (PAGE) Study
Source: PLoS Genet. 2011 Jun 30;7(6):e1002138. doi: 10.1371/journal.pgen.1002138 (PMC3128106; doi:10.1371/journal.pgen.1002138)

**Figure S2. Coded allele frequency across PAGE study sites, by population. European Americans**

**
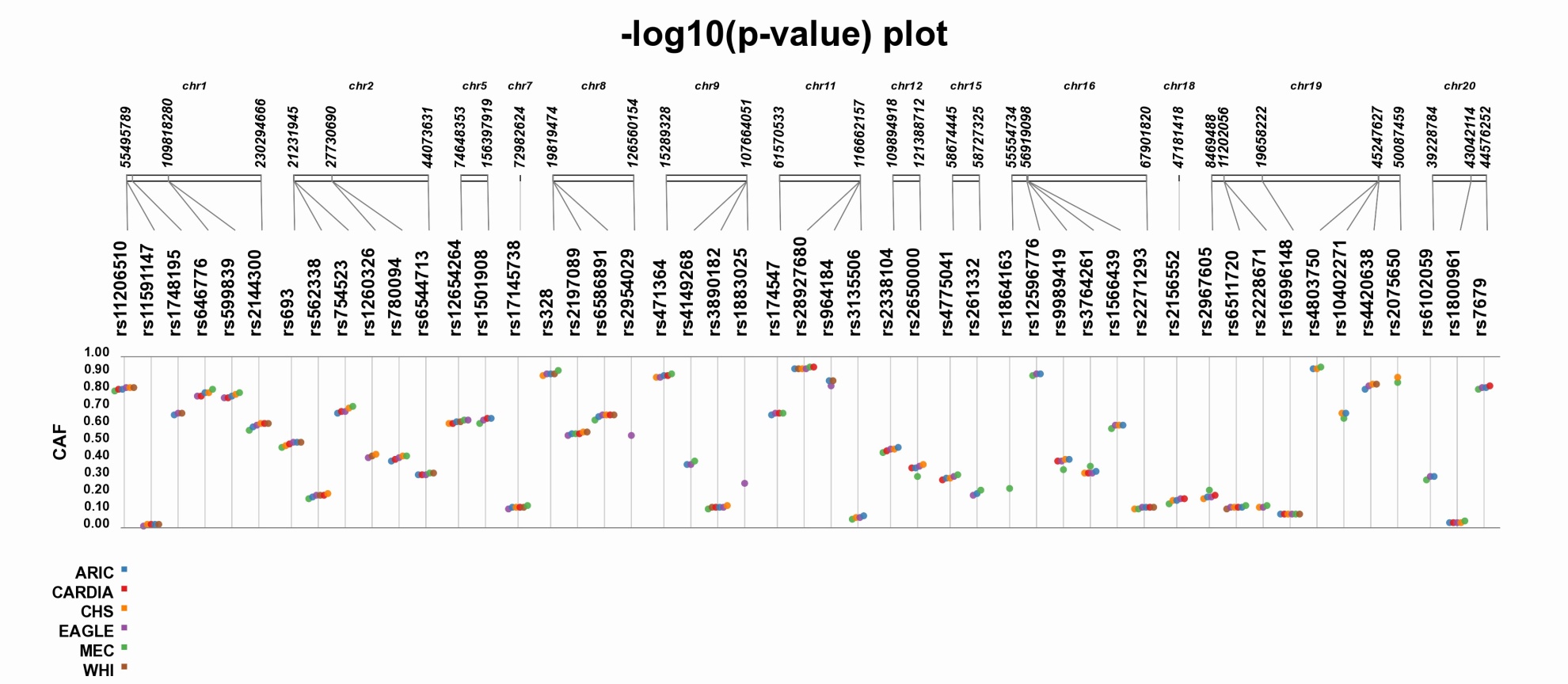
**

1. **African Americans**

**
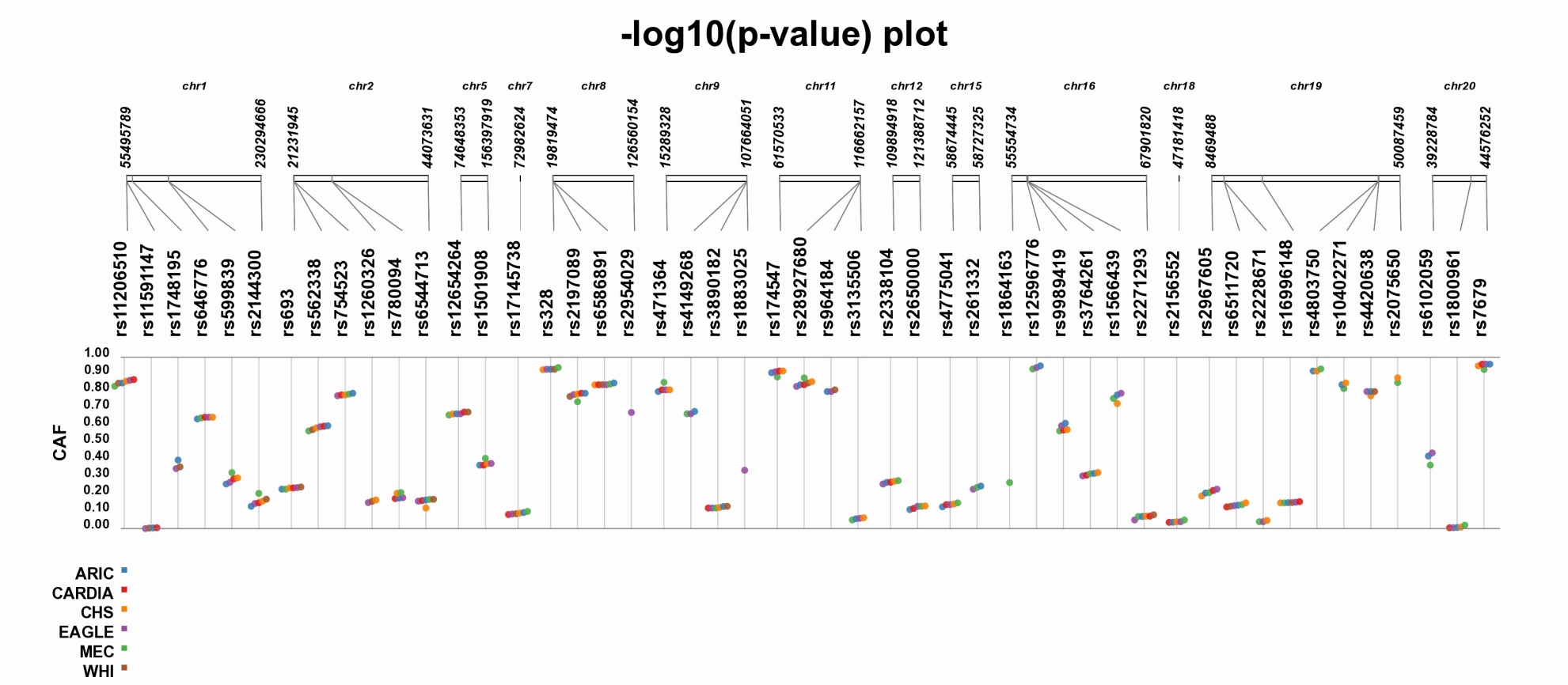
**

1. **American Indians**

**
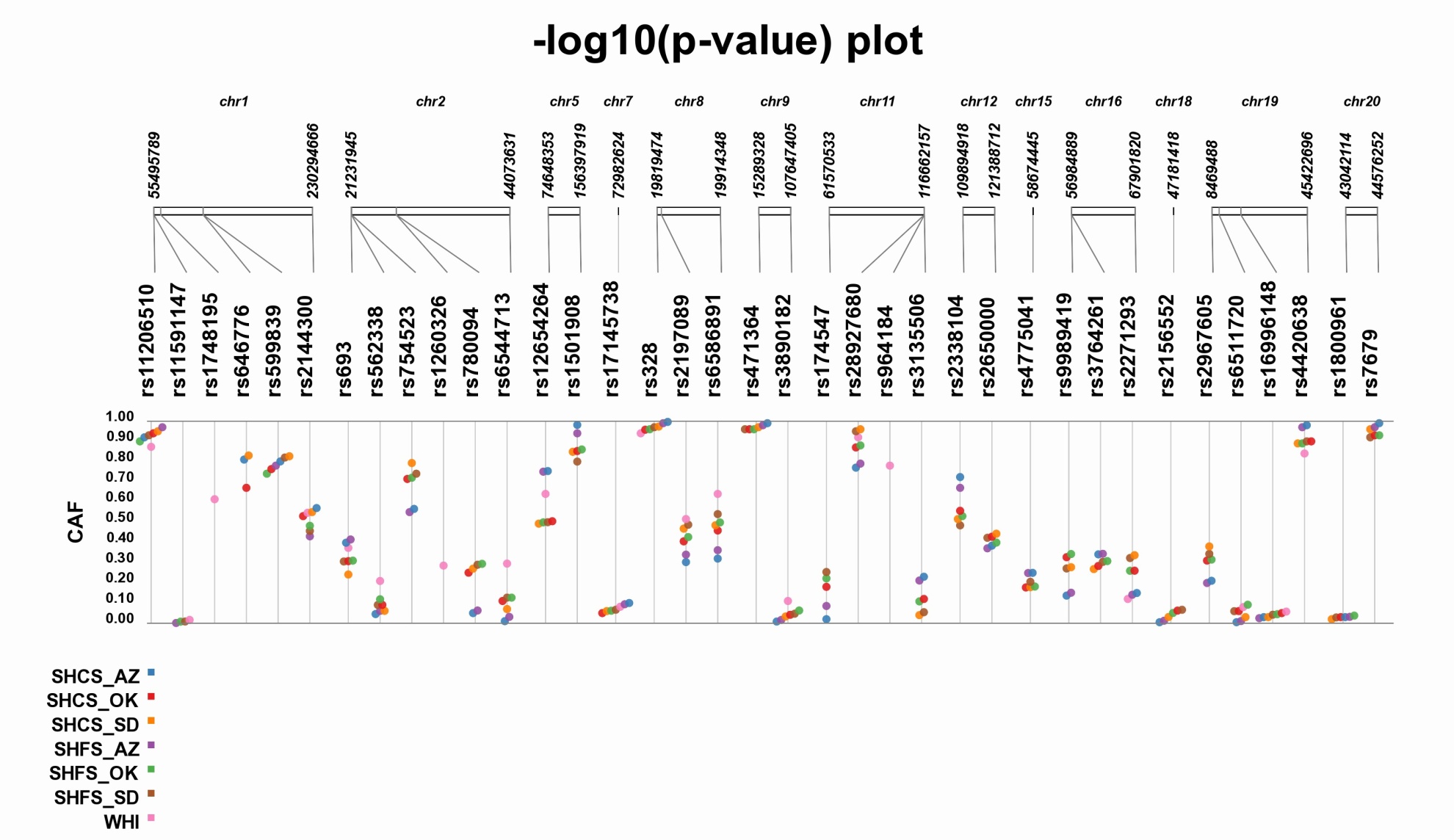
**

1. **Mexican Americans/Hispanics**

**
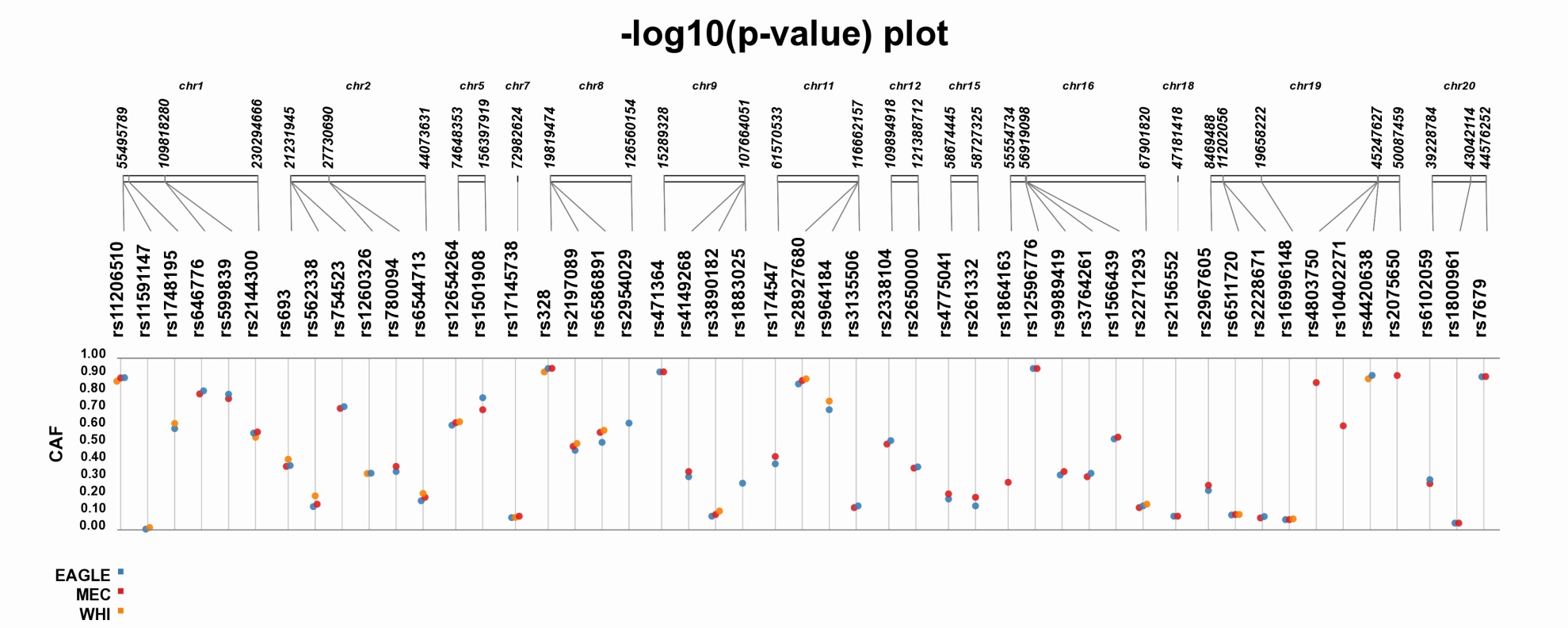
**

1. **Japanese/East Asians**

**
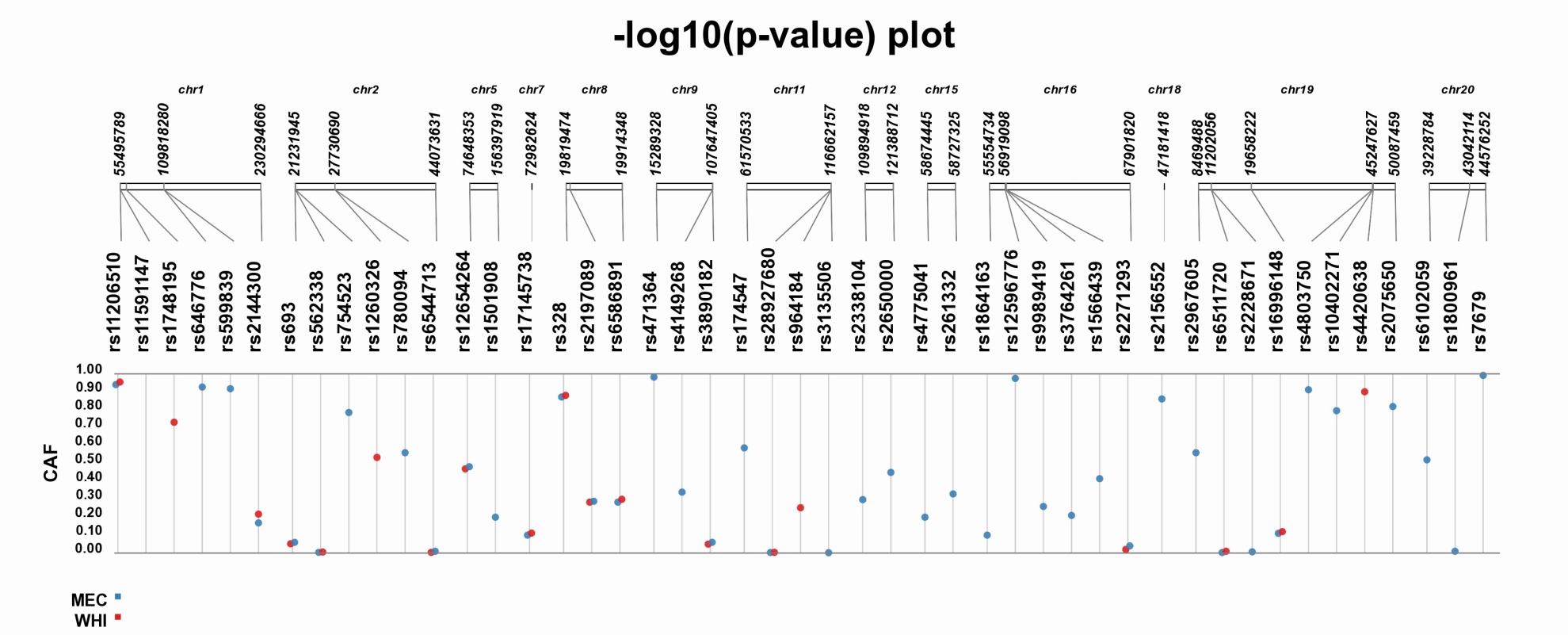
**

1. **Native Hawaiians/Pacific Islanders**


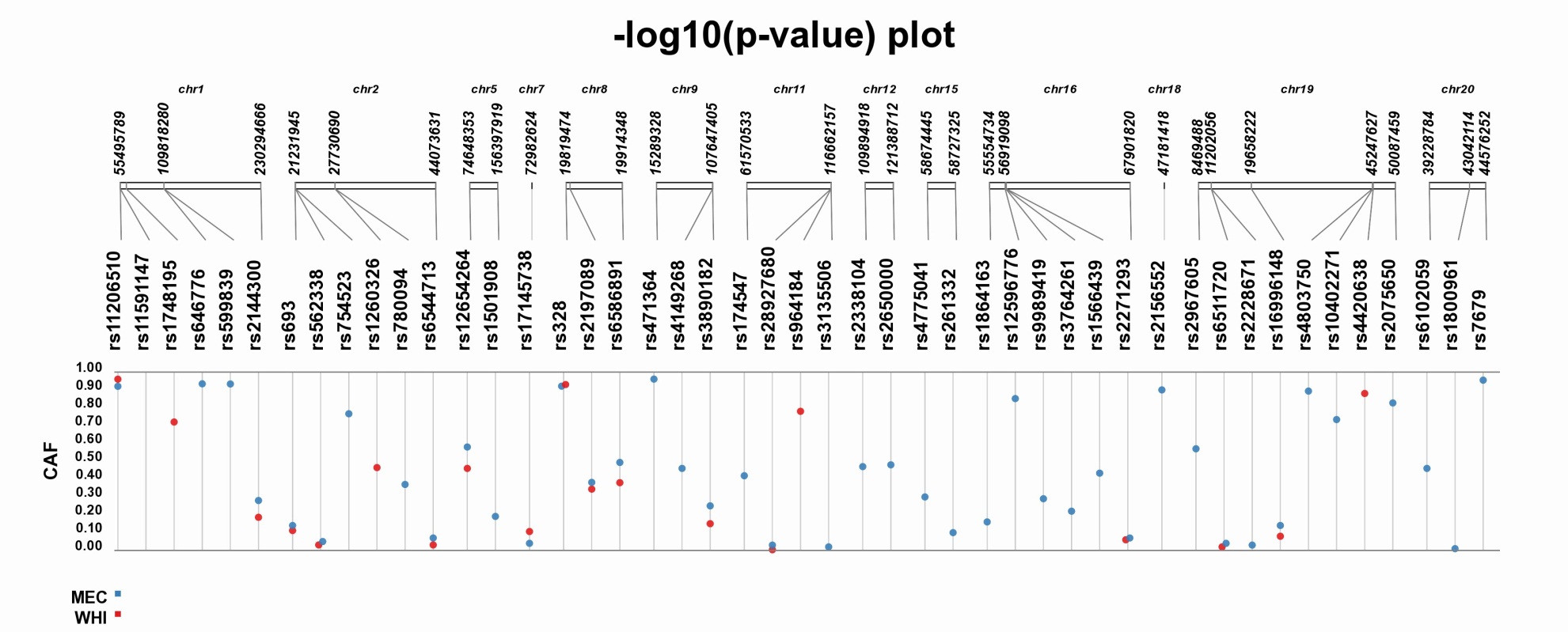

Supplement: Figure S2 — Coded allele frequency across PAGE study sites, by population. The coded allele frequency (CAF) is plotted for each of the 49 SNPs by population using Synthesis-View [73], [74]. The studies include: Atherosclerosis Risk in Communities (ARIC), Coronary Artery Risk in Young Adults (CARDIA), Cardiovascular Heart Study (CHS), Epidemiologic Architecture for Genes Linked to Environment (EAGLE), Multiethnic Cohort (MEC), Women's Health Initiative (WHI), Strong Heart Community Study (SHCS), and Strong Heart Family Study (SHFS) in Arizona (AZ), Oklahoma (OK) and South Dakota (SD). (DOCX) [file pgen.1002138.s002.docx]
