# Supplementary material for: Genetic Determinants of Lipid Traits in Diverse Populations from the Population Architecture using Genomics and Epidemiology (PAGE) Study
Source: PLoS Genet. 2011 Jun 30;7(6):e1002138. doi: 10.1371/journal.pgen.1002138 (PMC3128106; doi:10.1371/journal.pgen.1002138)
Supplement: Figure S4 — Comparison of unadjusted, minimally adjusted, adjusted models for HDL-C, by population. Results of tests of association for four regression models are plotted: model 1 (unadjusted), model 2 (adjusted for age and sex; and site of ascertainment for select PAGE studies), model 3 (adjusted for age, sex, body mass index, current smoking, type 2 diabetes, post-menopausal status, and current hormone use), and model 4 (model 3 with the addition of previous myocardial infarction). Each SNP was tested for an association with HDL-C. Meta-analysis was performed, and p-values (−log10 transformed) of the meta-analysis are plotted along the y-axis. SNP location is given on the x-axis. Each triangle represents a meta-analysis p-value for each population. Models are color coded. Large triangles represent p-values at or smaller than genome-wide significance (p<10−8). The direction of the arrows corresponds to the direction of the beta coefficient. The exact beta coefficients are reported on the bottom panel. The significance threshold is indicated by the red bar at p = 0.05. (DOCX) [file pgen.1002138.s004.docx]

**Figure S4.** **Comparison of unadjusted, minimally adjusted, adjusted models for HDL-C, by population.**

1. **European Americans**

**
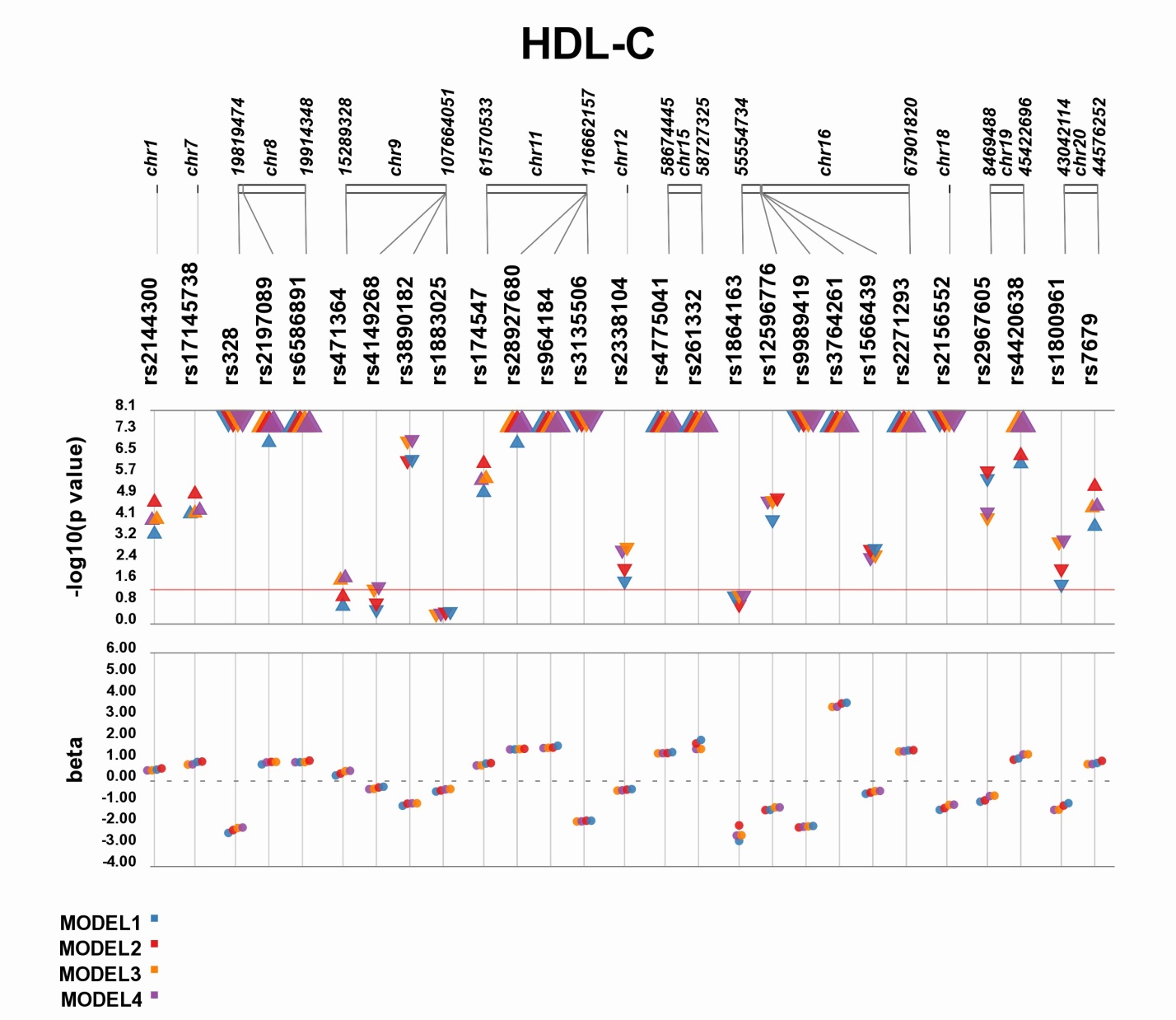
**

1. **African Americans**

**
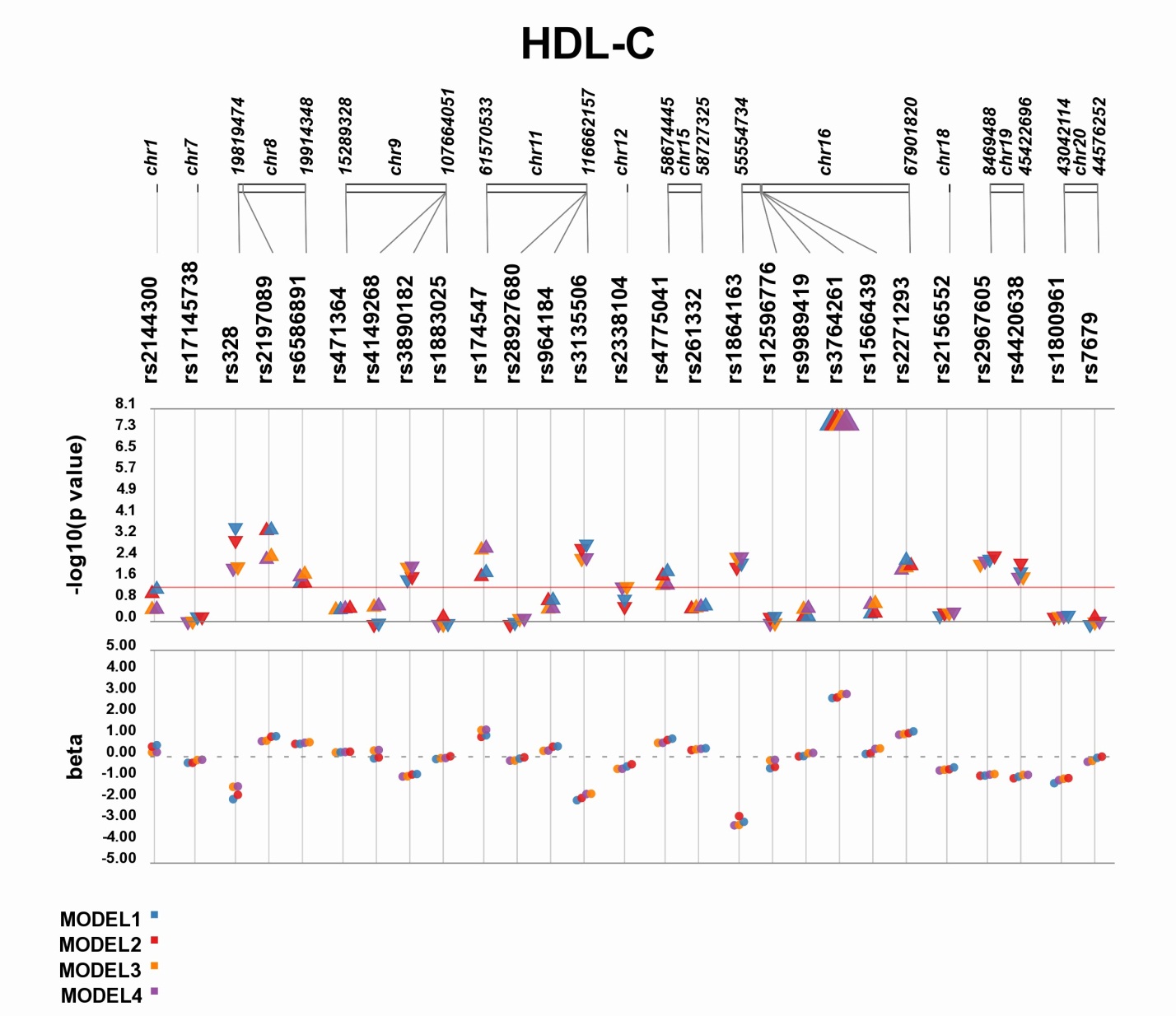
**

1. **American Indians**

**
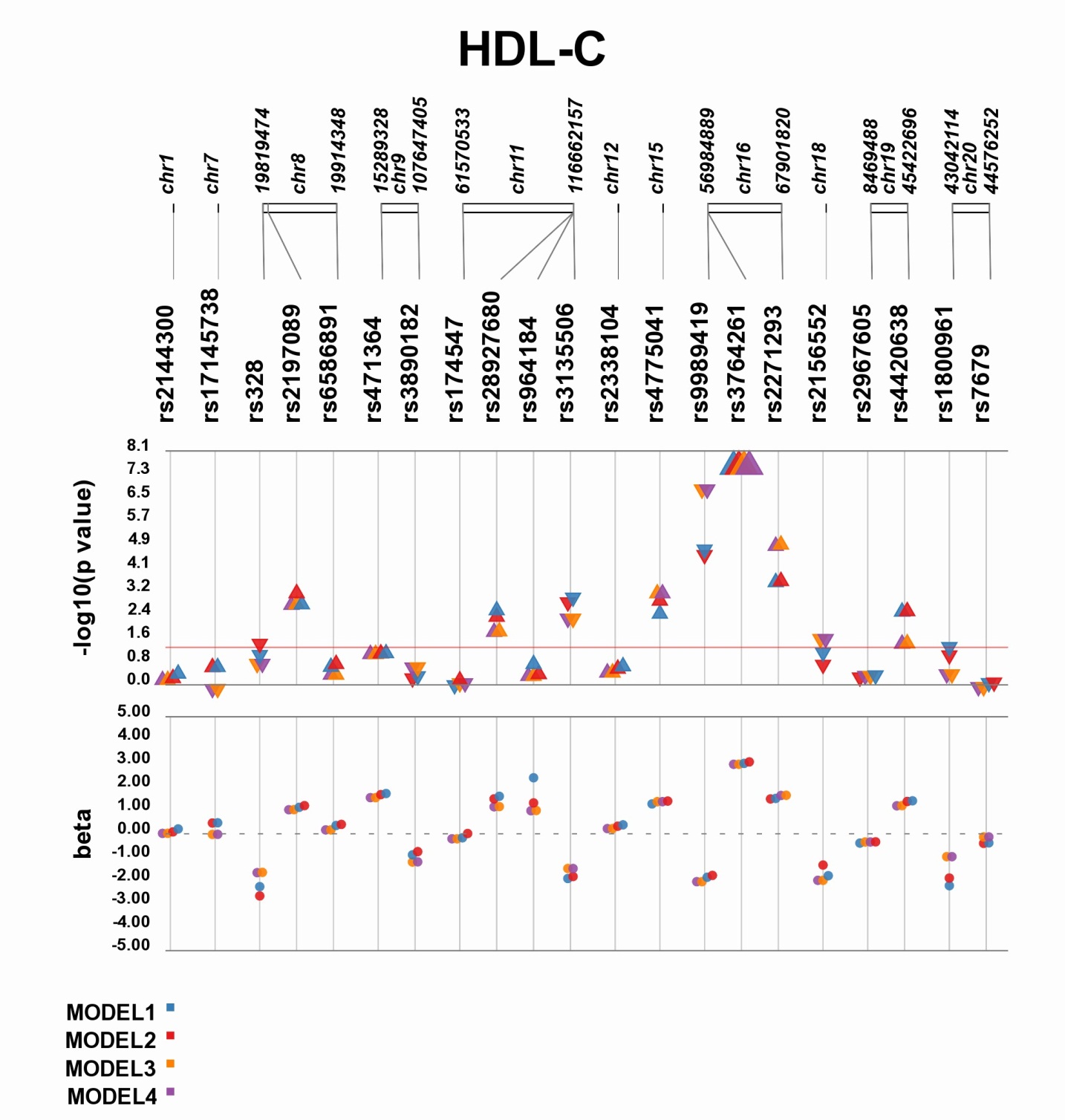
**

1. **Mexican Americans/Hispanics**

**
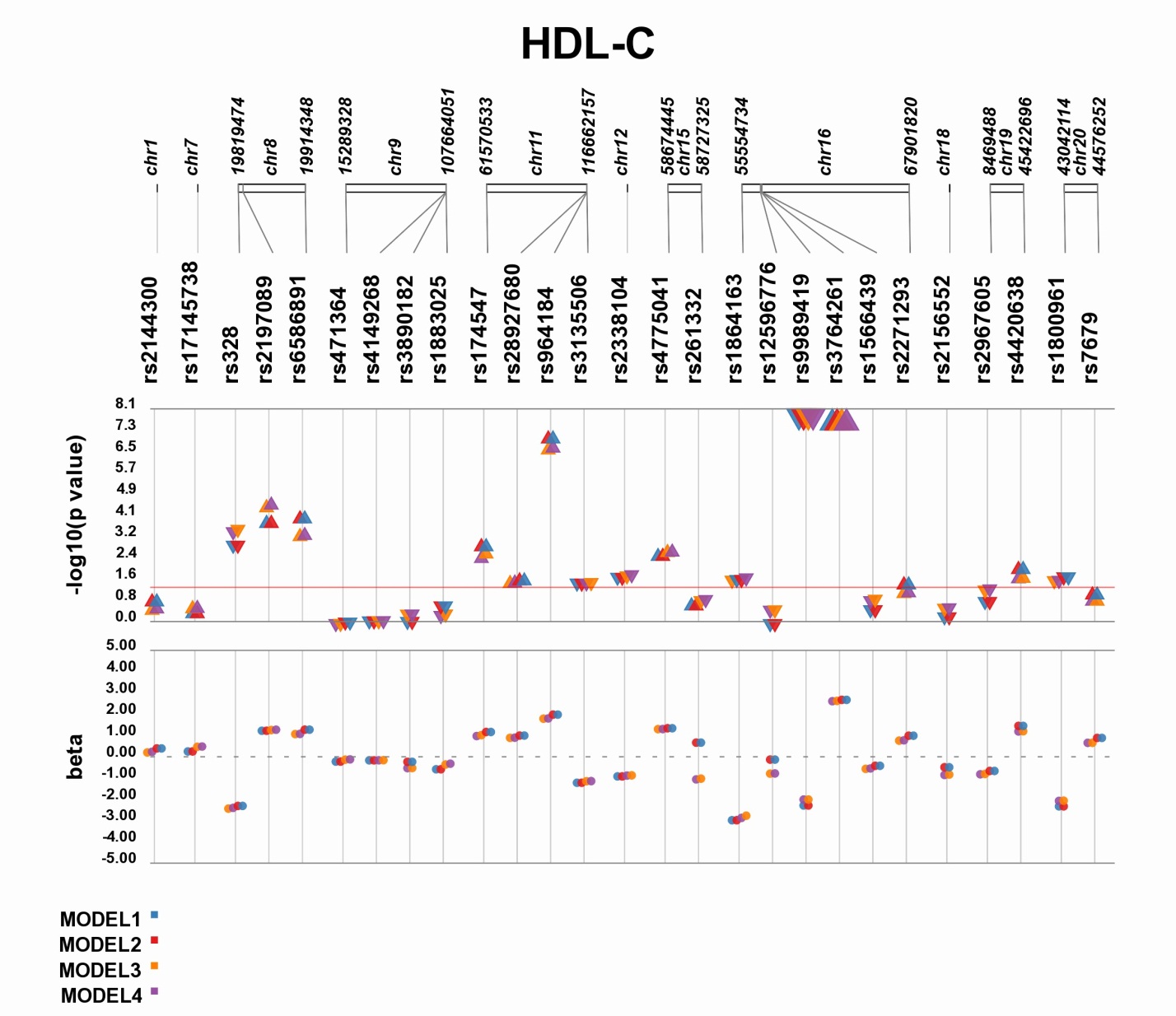
**

1. **Japanese/East Asians**

**
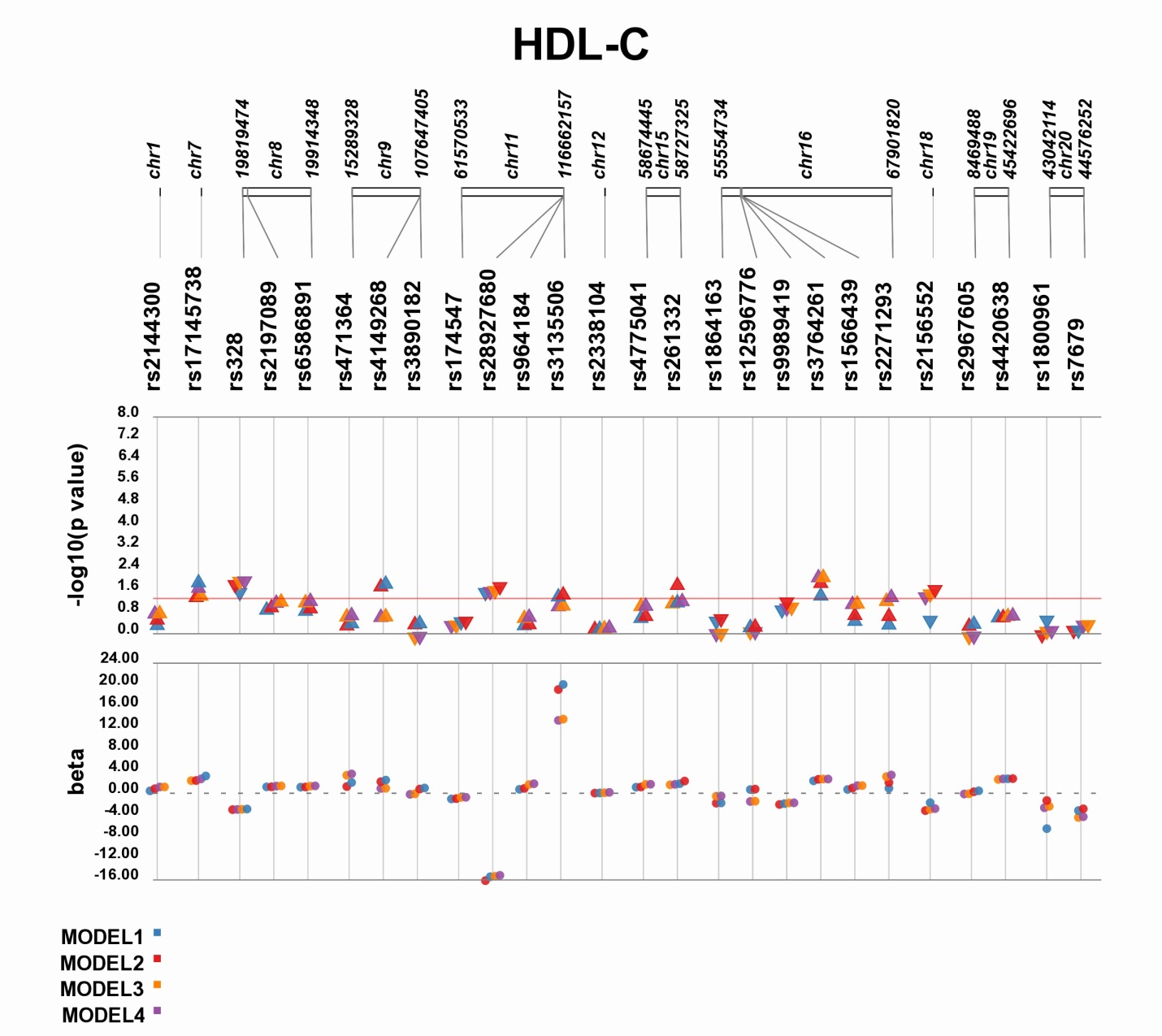
**

**f) Native Hawaiians/Pacific Islanders**

**
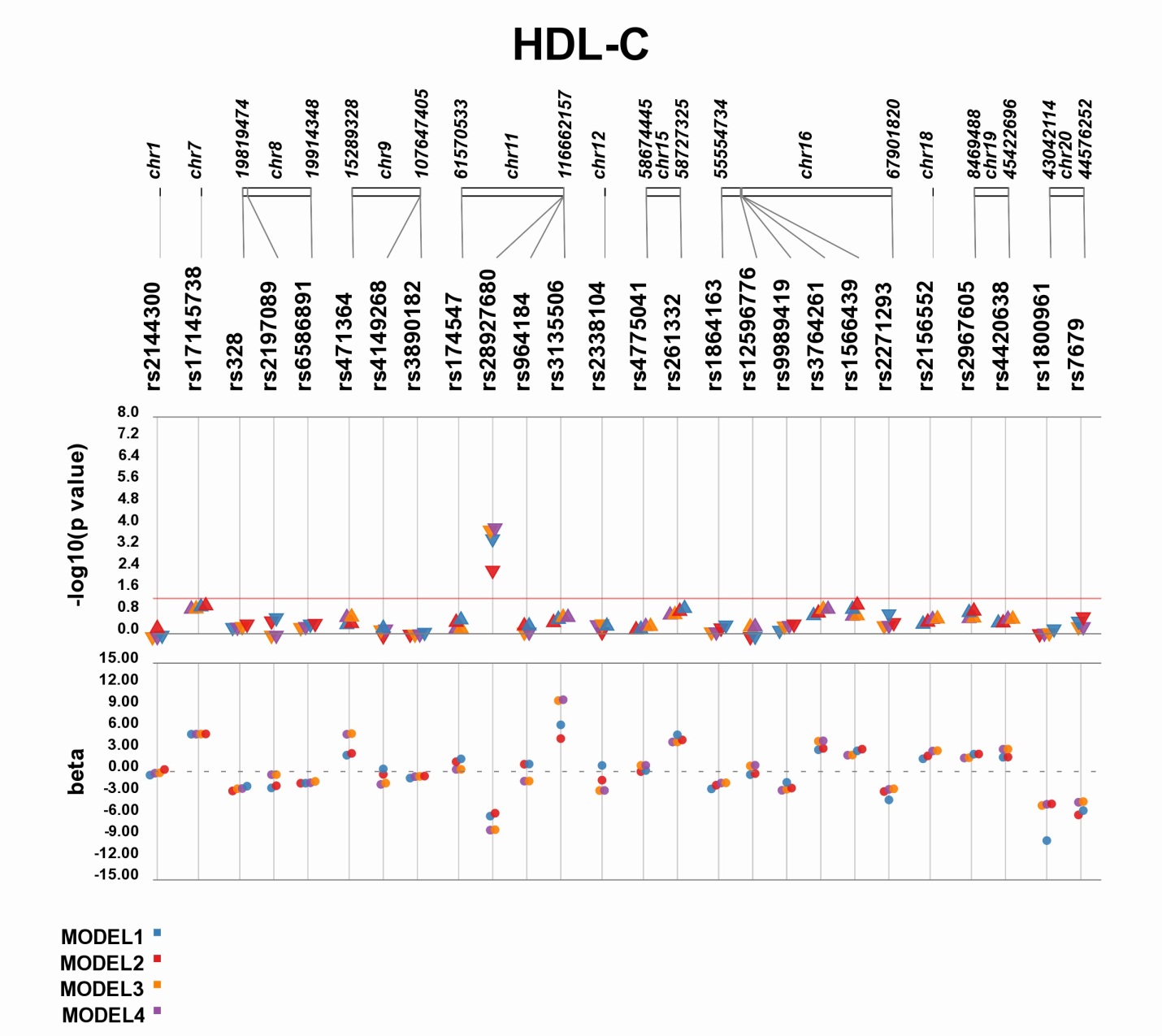
**
