# Supplementary material for: Genetic Determinants of Lipid Traits in Diverse Populations from the Population Architecture using Genomics and Epidemiology (PAGE) Study
Source: PLoS Genet. 2011 Jun 30;7(6):e1002138. doi: 10.1371/journal.pgen.1002138 (PMC3128106; doi:10.1371/journal.pgen.1002138)

**Figure S7.**  **Comparison of genetic effect estimates when participants are excluded or included based on medication use with adjustments in WHI.**

1. **HDL-C**

**
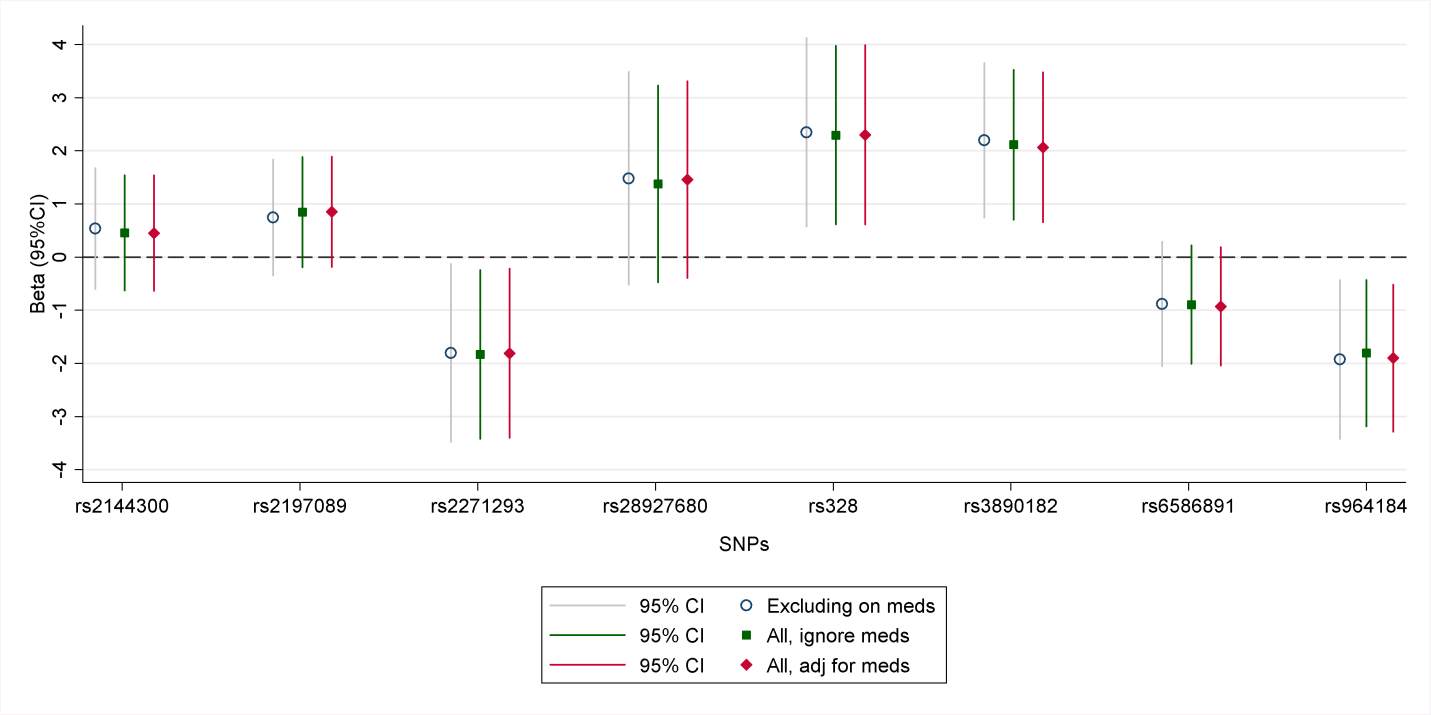
**

1. **LDL-C**


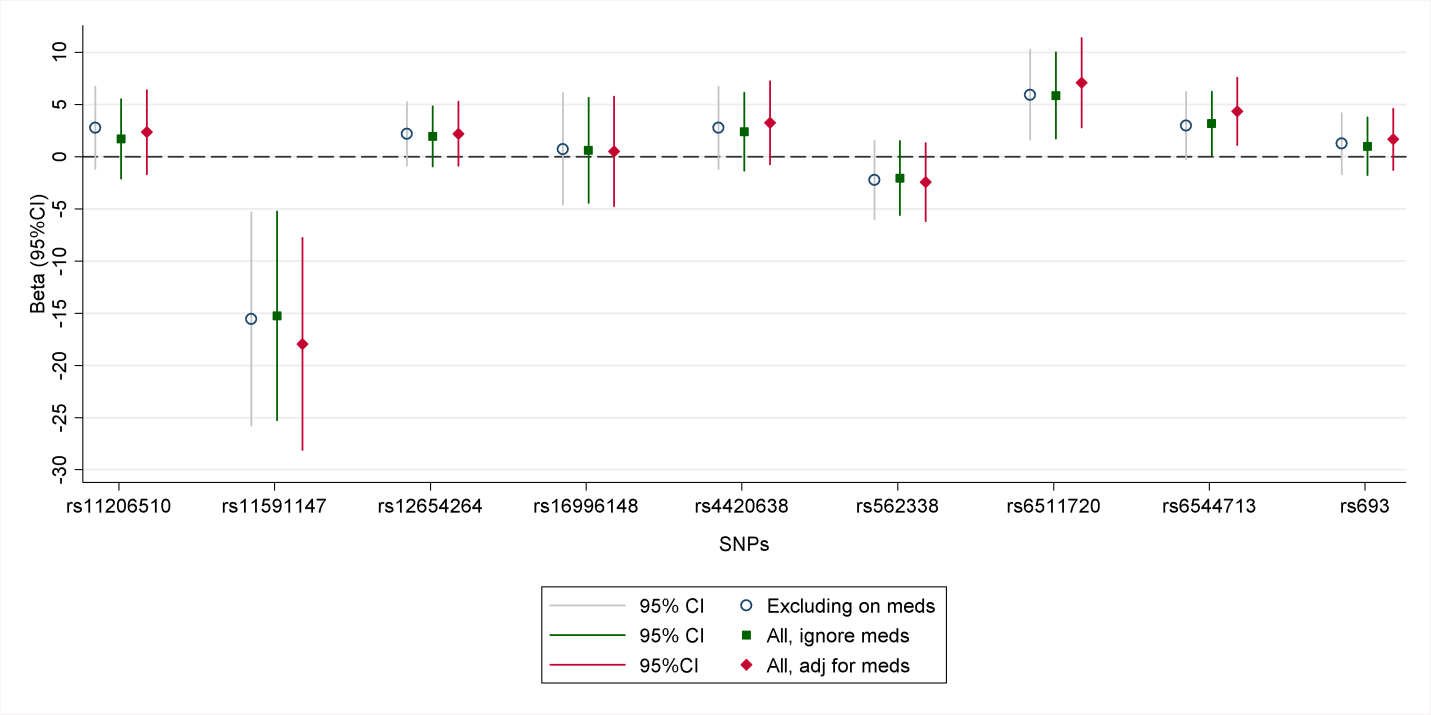


1. **ln(TG)**


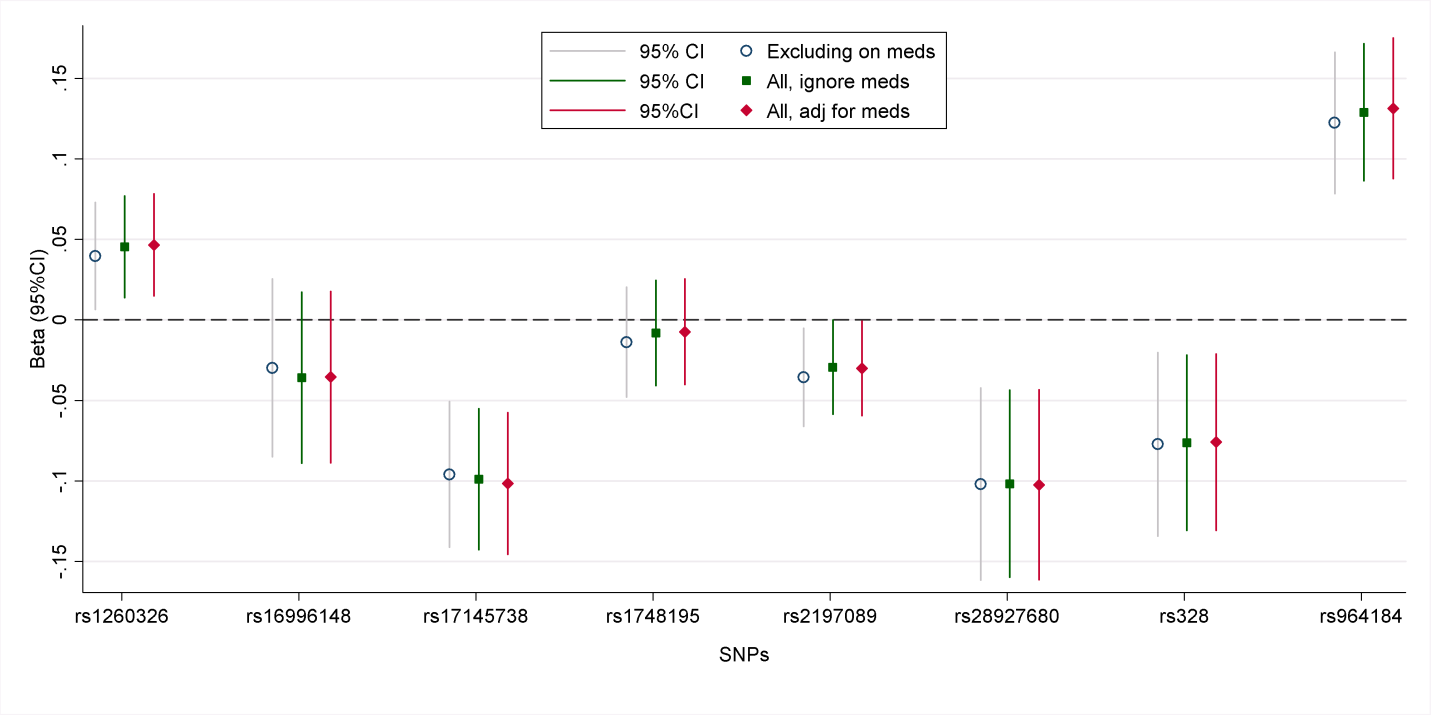

Supplement: Figure S7 — Comparison of genetic effect estimates when participants are excluded or included based on medication use with adjustments in WHI. Genetic effect estimates (β) and 95% confidence interval are plotted for each SNP tested for an association. The tests of association were performed on fasting European Americans adjusted for age and sex and excluding participants on lipid lowering medication (blue), including all participants regardless of medication use (green), and all participants on lipid lowering medication, adjusted for the average HDL-C, LDL-C, and ln(TG) effects estimated by Wu et al [87]. (DOCX) [file pgen.1002138.s007.docx]
