# Supplementary material for: Genetic Determinants of Lipid Traits in Diverse Populations from the Population Architecture using Genomics and Epidemiology (PAGE) Study
Source: PLoS Genet. 2011 Jun 30;7(6):e1002138. doi: 10.1371/journal.pgen.1002138 (PMC3128106; doi:10.1371/journal.pgen.1002138)
Supplement: Figure S10 — Transformed triglycerides and the effects of lipid lowering medication use on genetic associations, by population. Comparison of genetic effects and significance when tests of association are performed within fasting adults regardless of lipid lowering medication versus fasting adults not on lipid lowering medication. All tests of association results shown here are minimally adjusted for age and sex. (DOCX) [file pgen.1002138.s010.docx]

**Figure S10. Transformed triglycerides and the effects of lipid lowering medication use on genetic associations, by population.**

1. **European Americans**

**
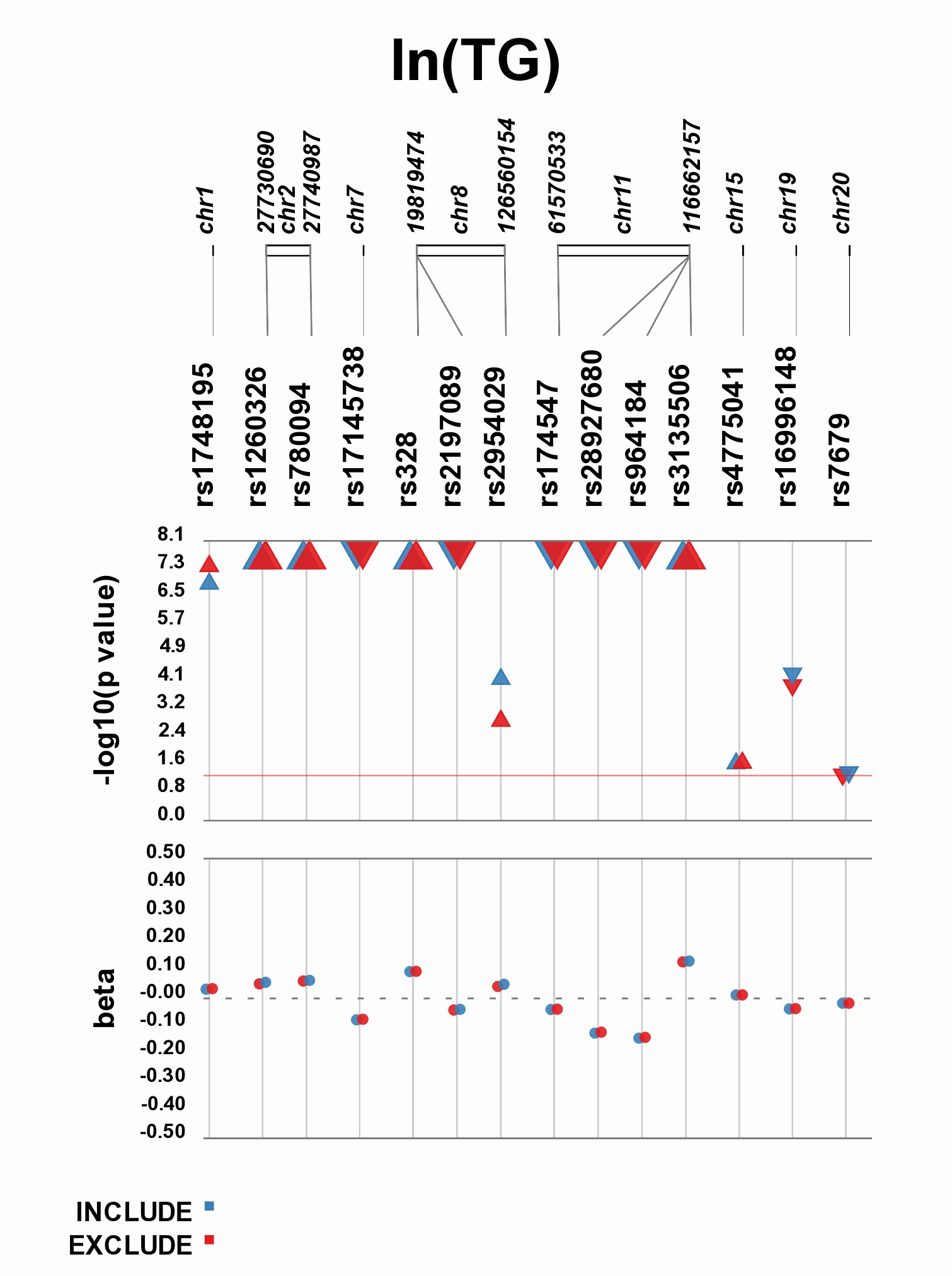
**

1. **African Americans**

**
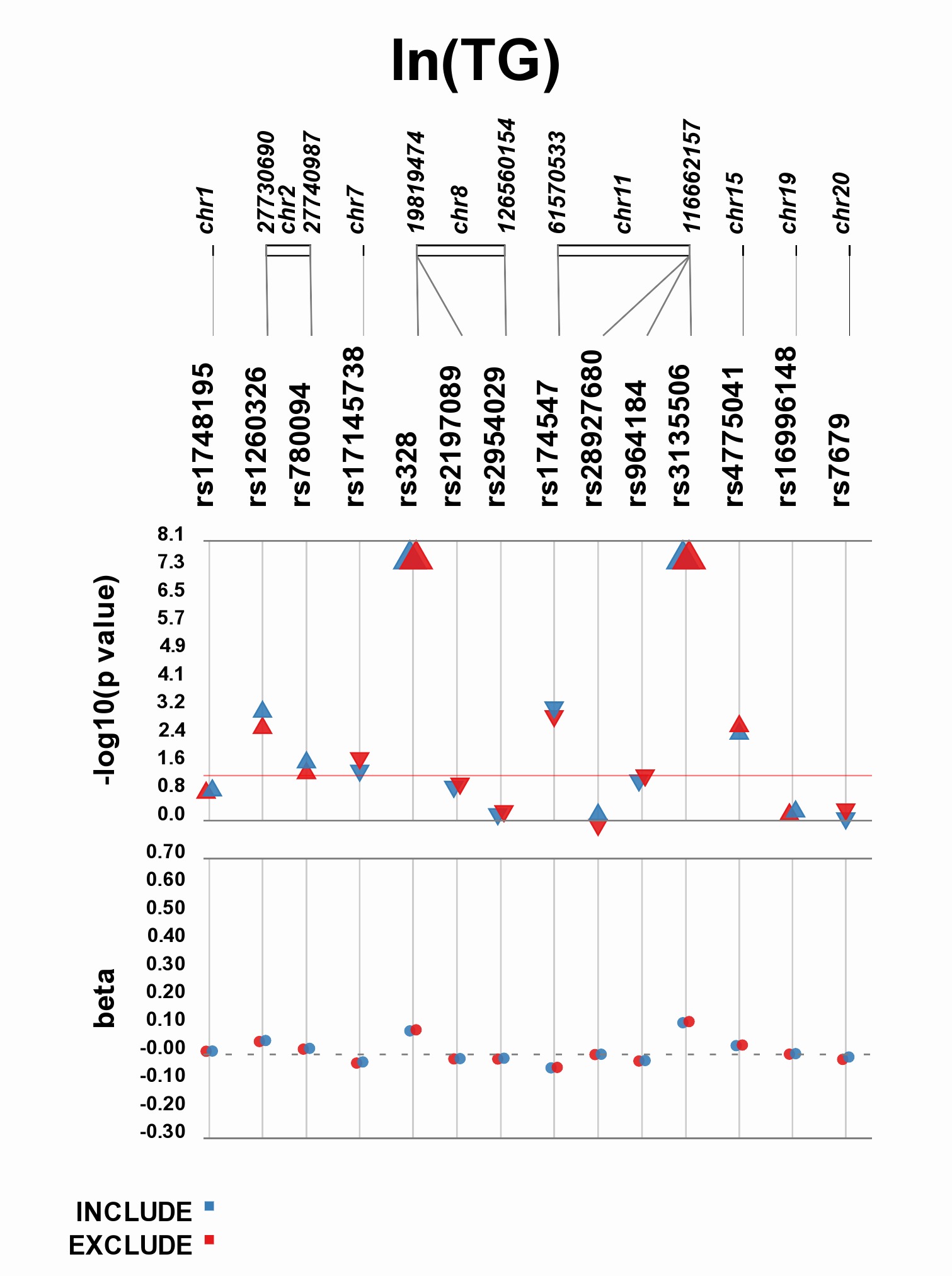
**

1. **American Indians**

**
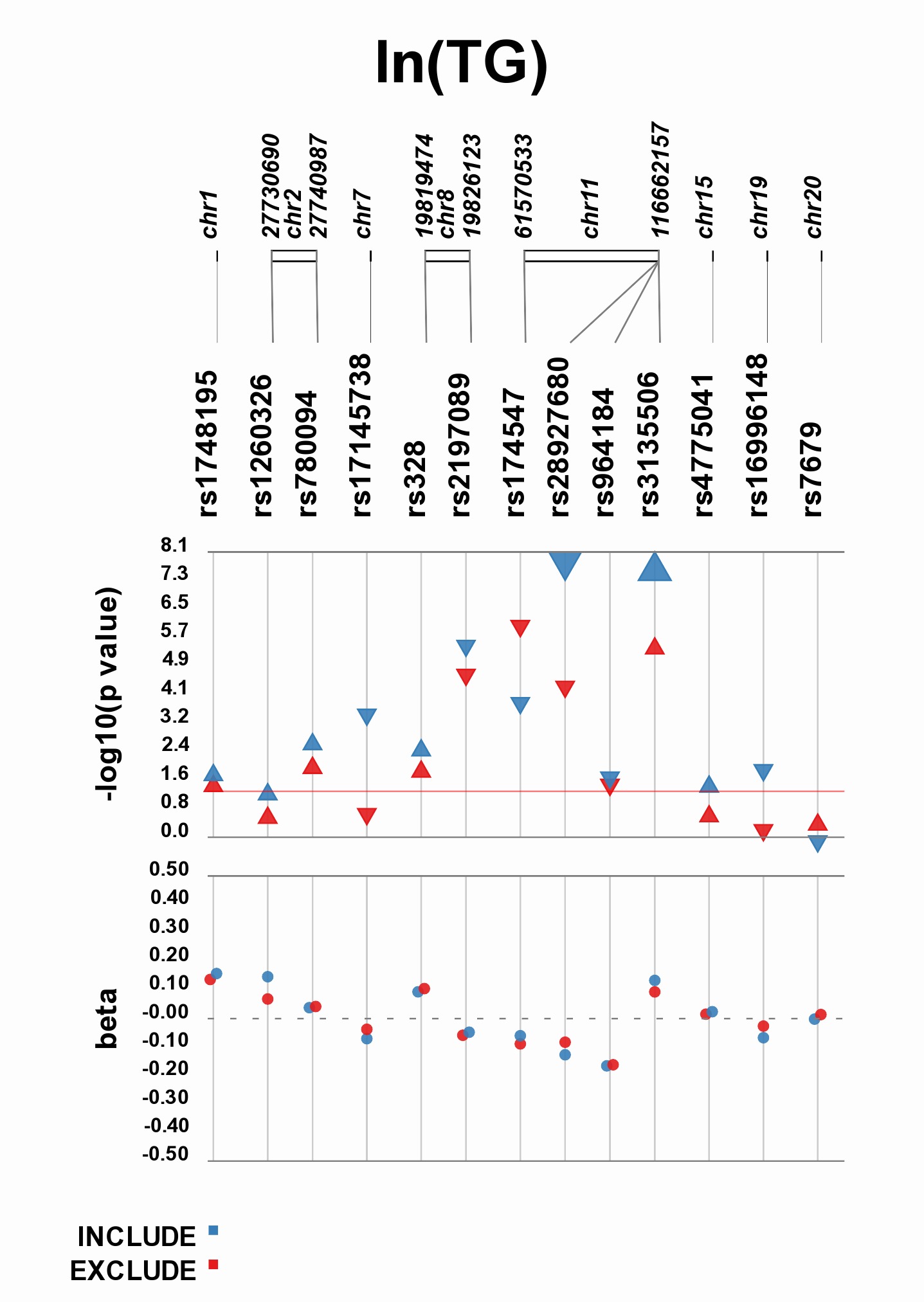
**

1. **Mexican Americans/Hispanics**

**
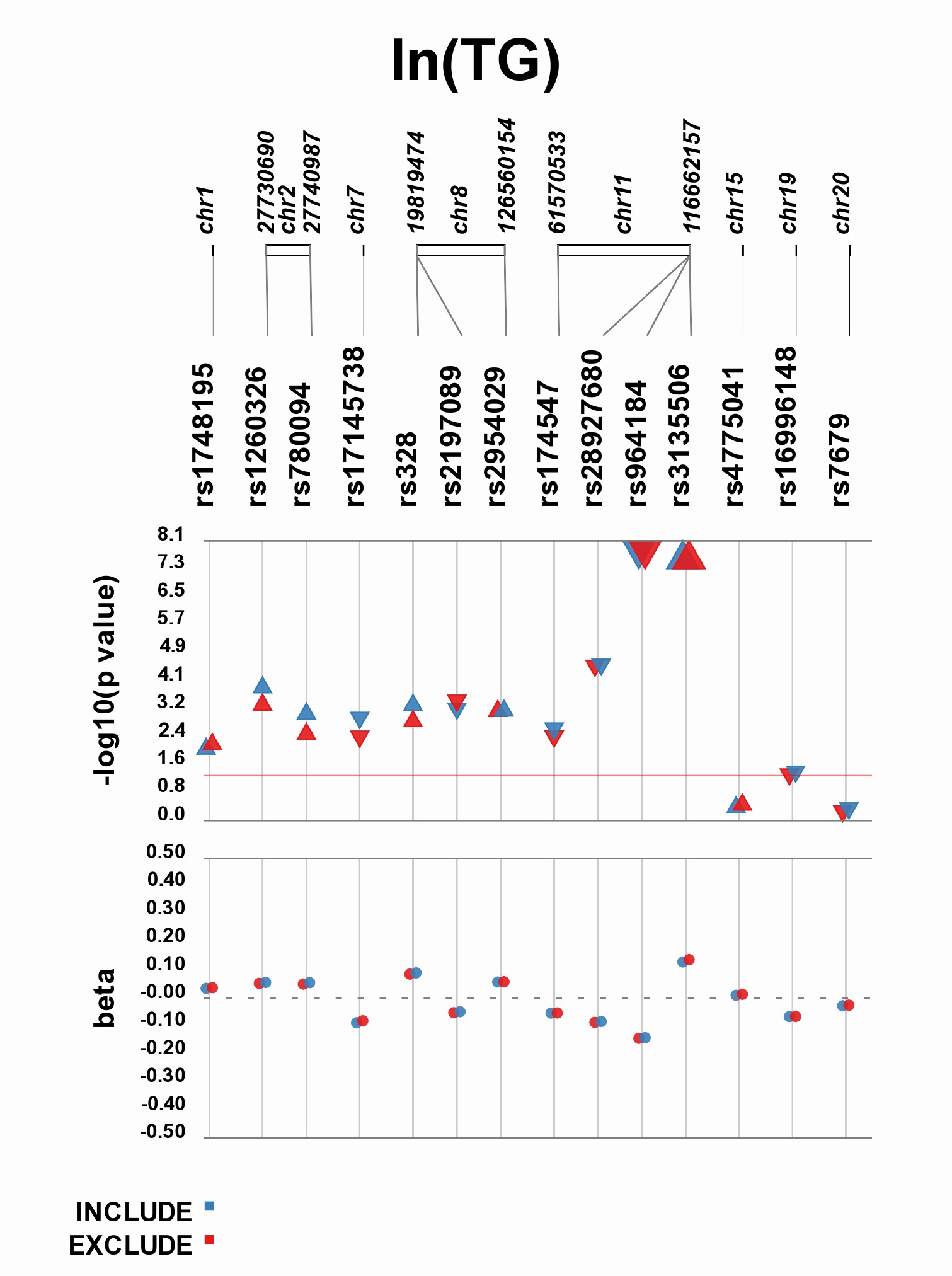
**

1. **Japanese/East Asians**

**
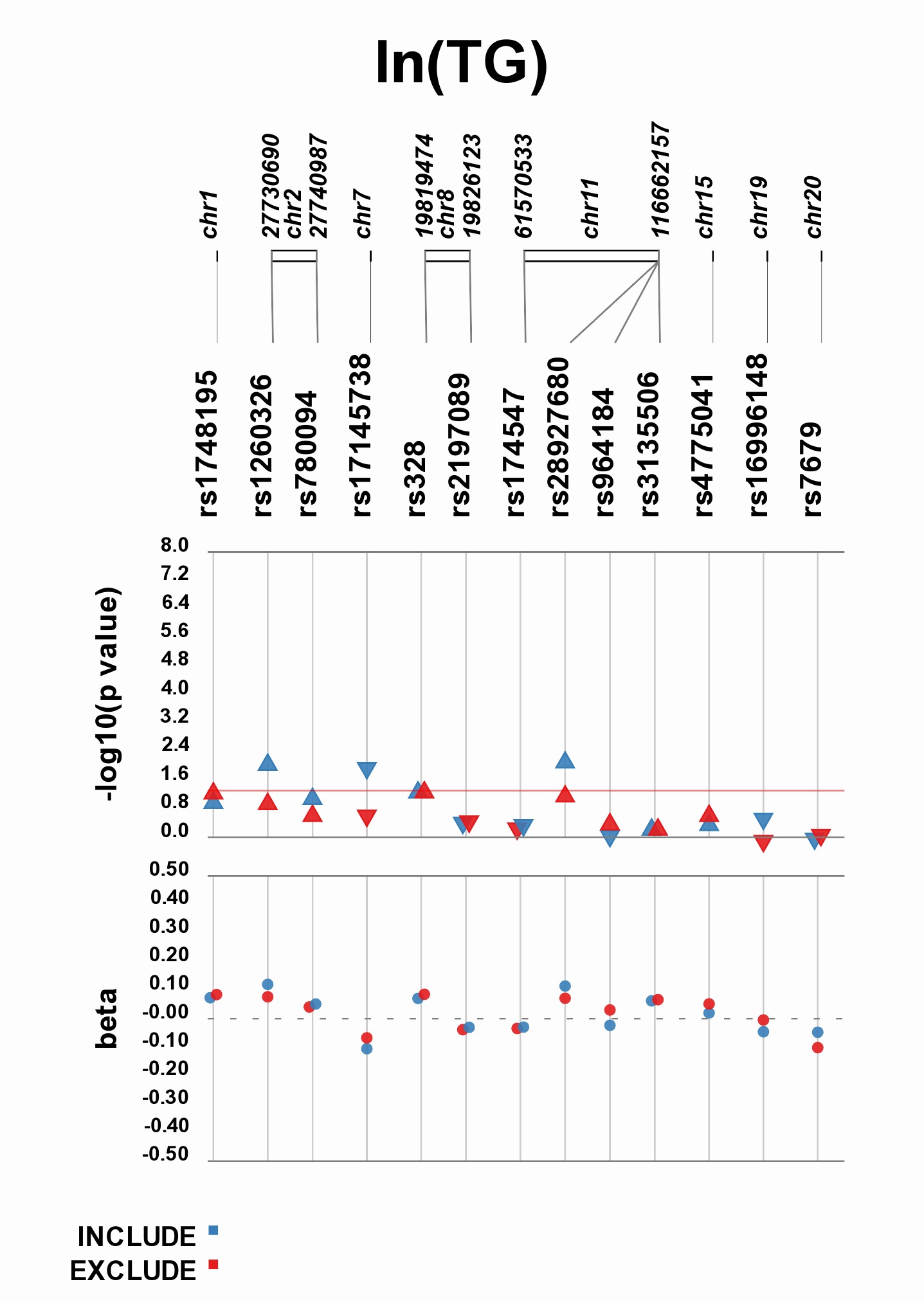
**

1. **Native Hawaiians/Pacific Islanders**

**
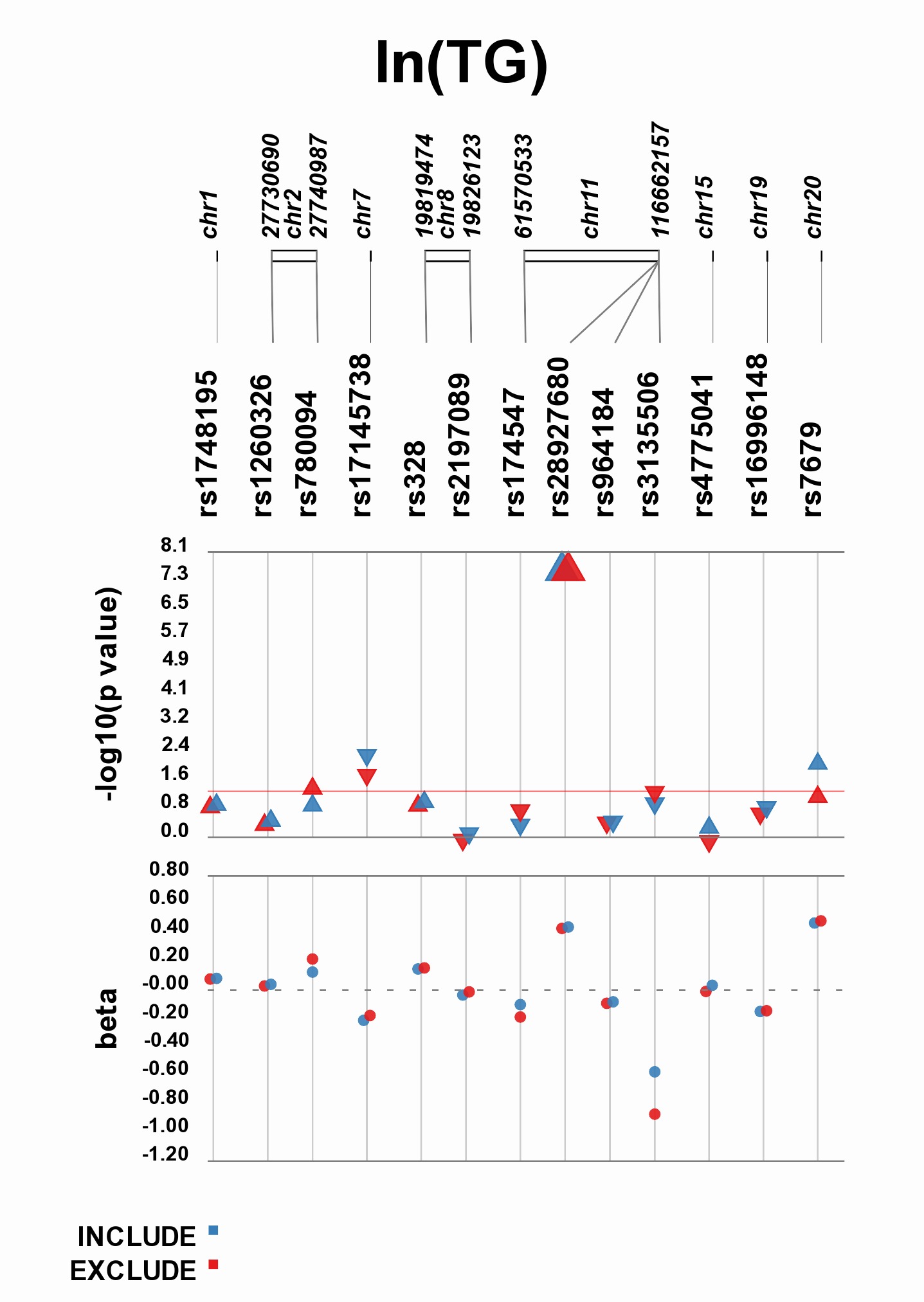
**
