# Supplementary material for: Genetic Determinants of Lipid Traits in Diverse Populations from the Population Architecture using Genomics and Epidemiology (PAGE) Study
Source: PLoS Genet. 2011 Jun 30;7(6):e1002138. doi: 10.1371/journal.pgen.1002138 (PMC3128106; doi:10.1371/journal.pgen.1002138)

**Figure S11.** **Comparison of HDL-C associations across PAGE study sites, by population.**

1. **European Americans**

**
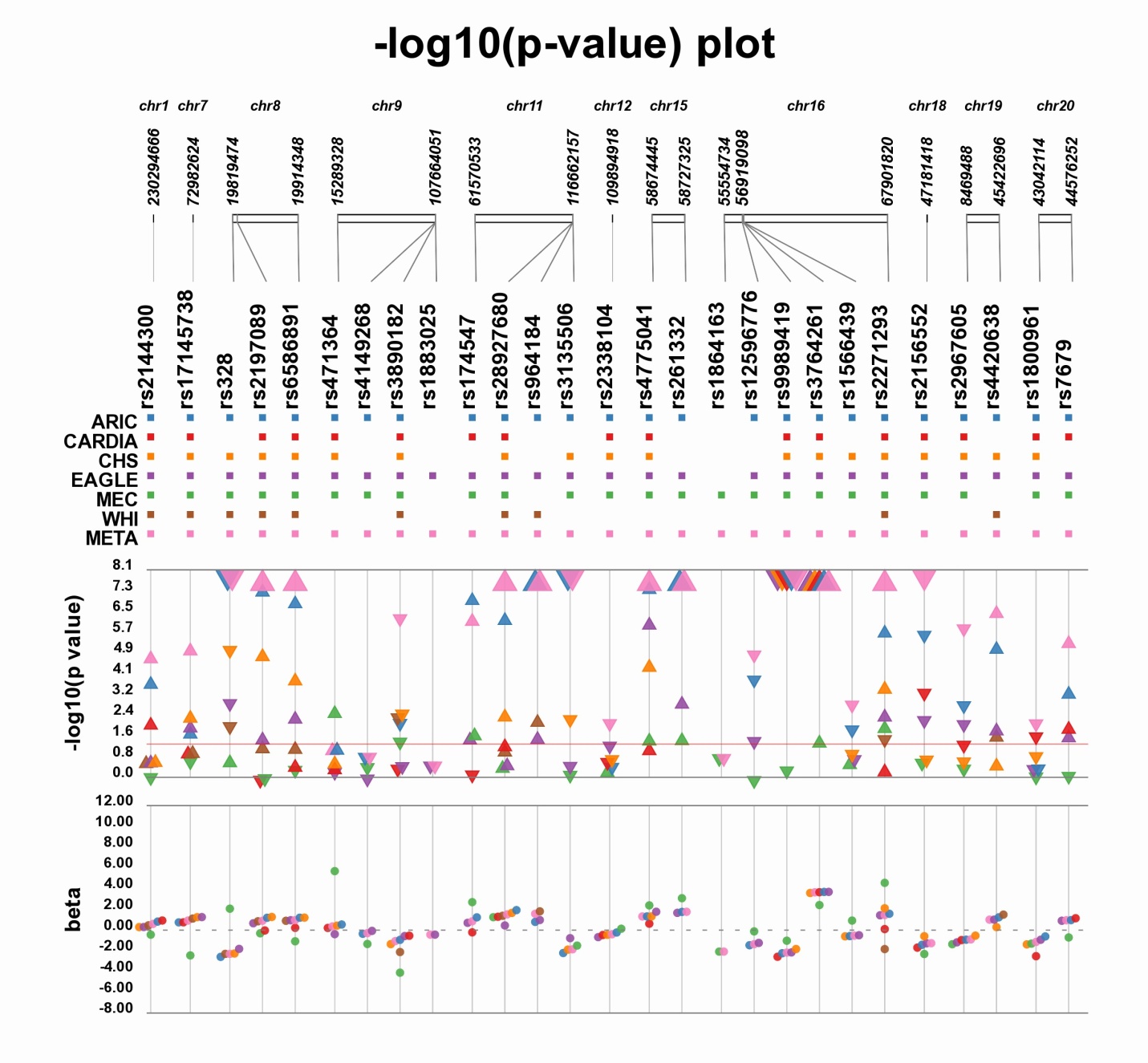
**

1. **African Americans**

**
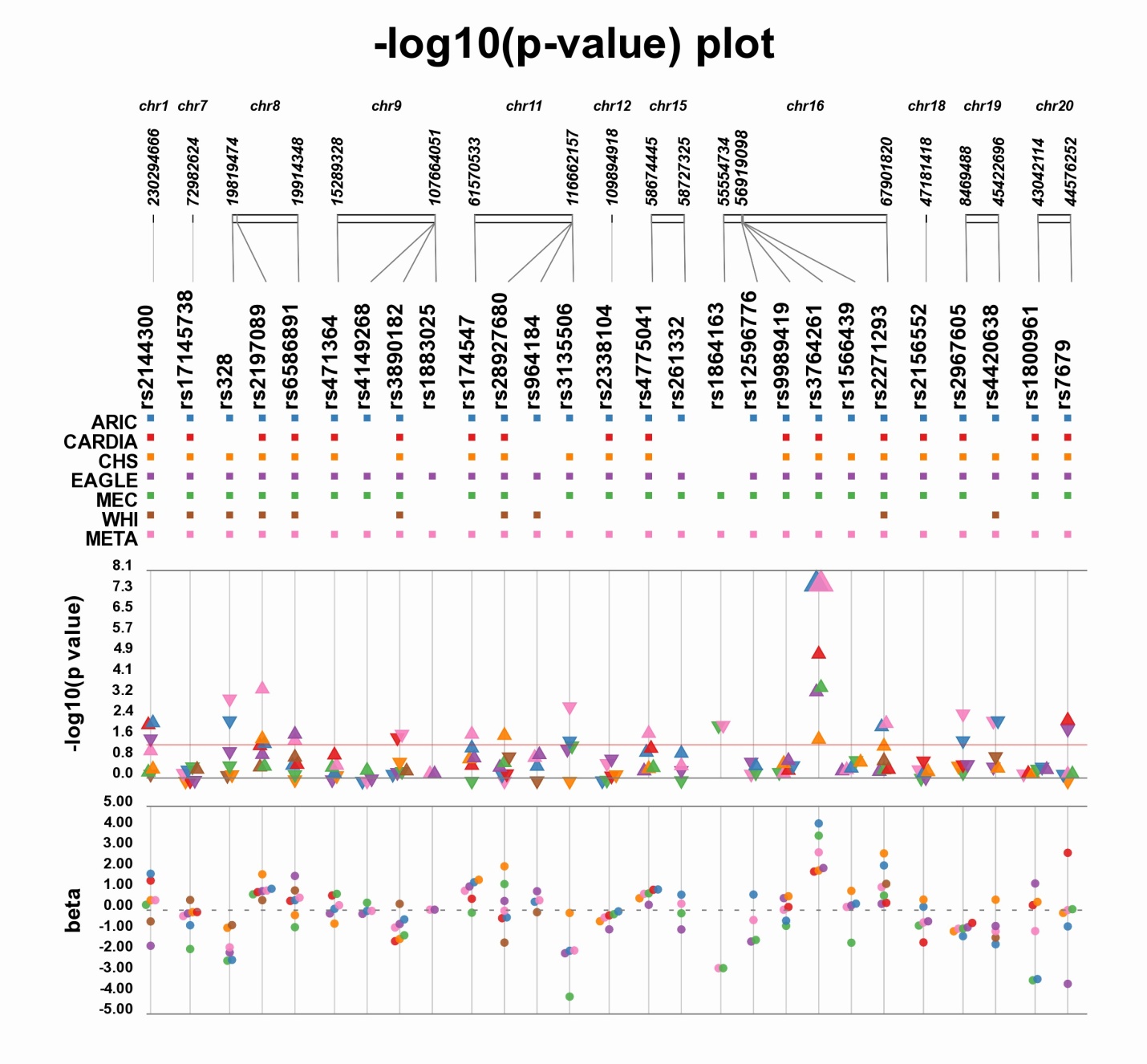
**

1. **American Indians**

**
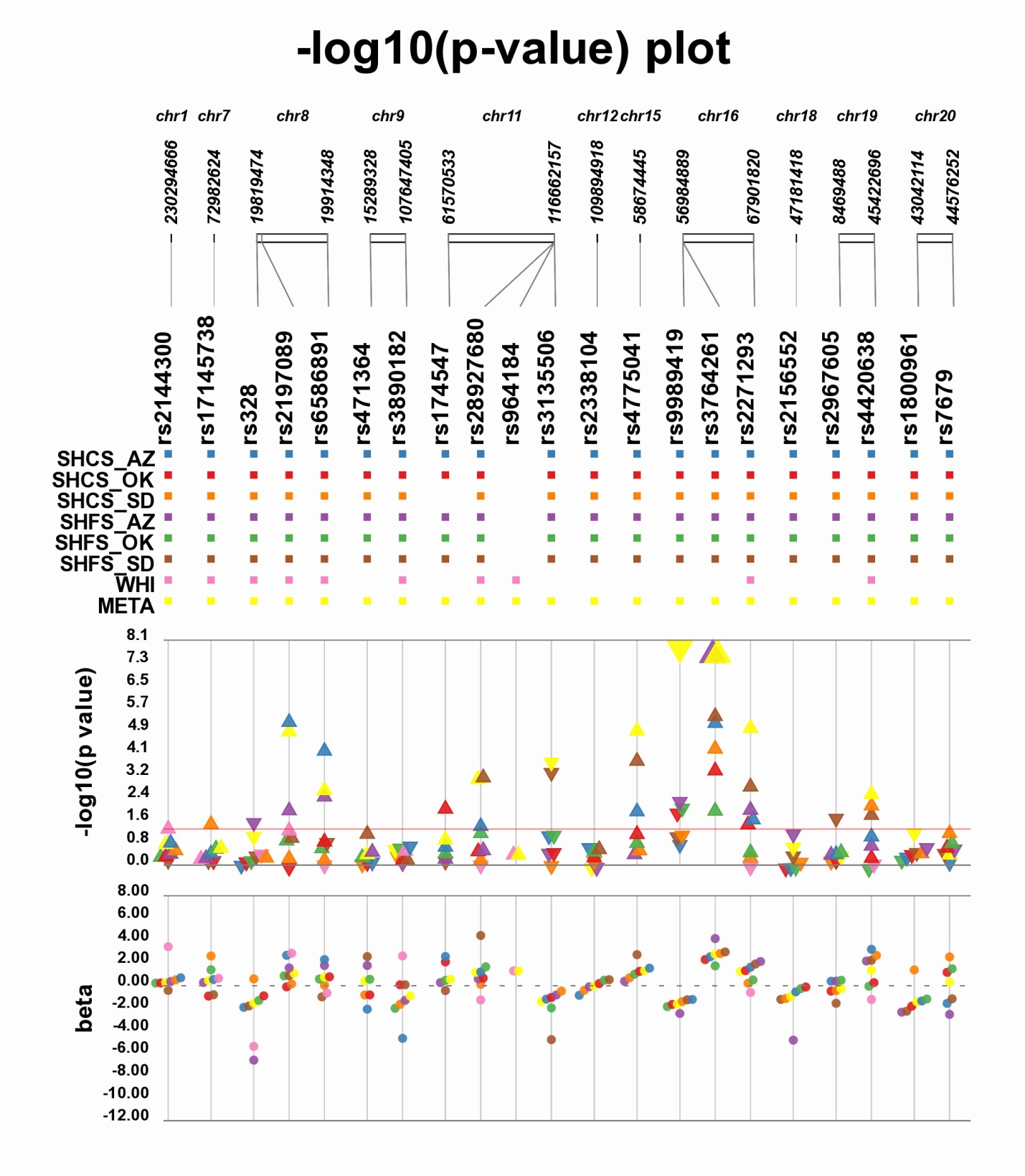
**

1. **Mexican Americans/Hispanics**


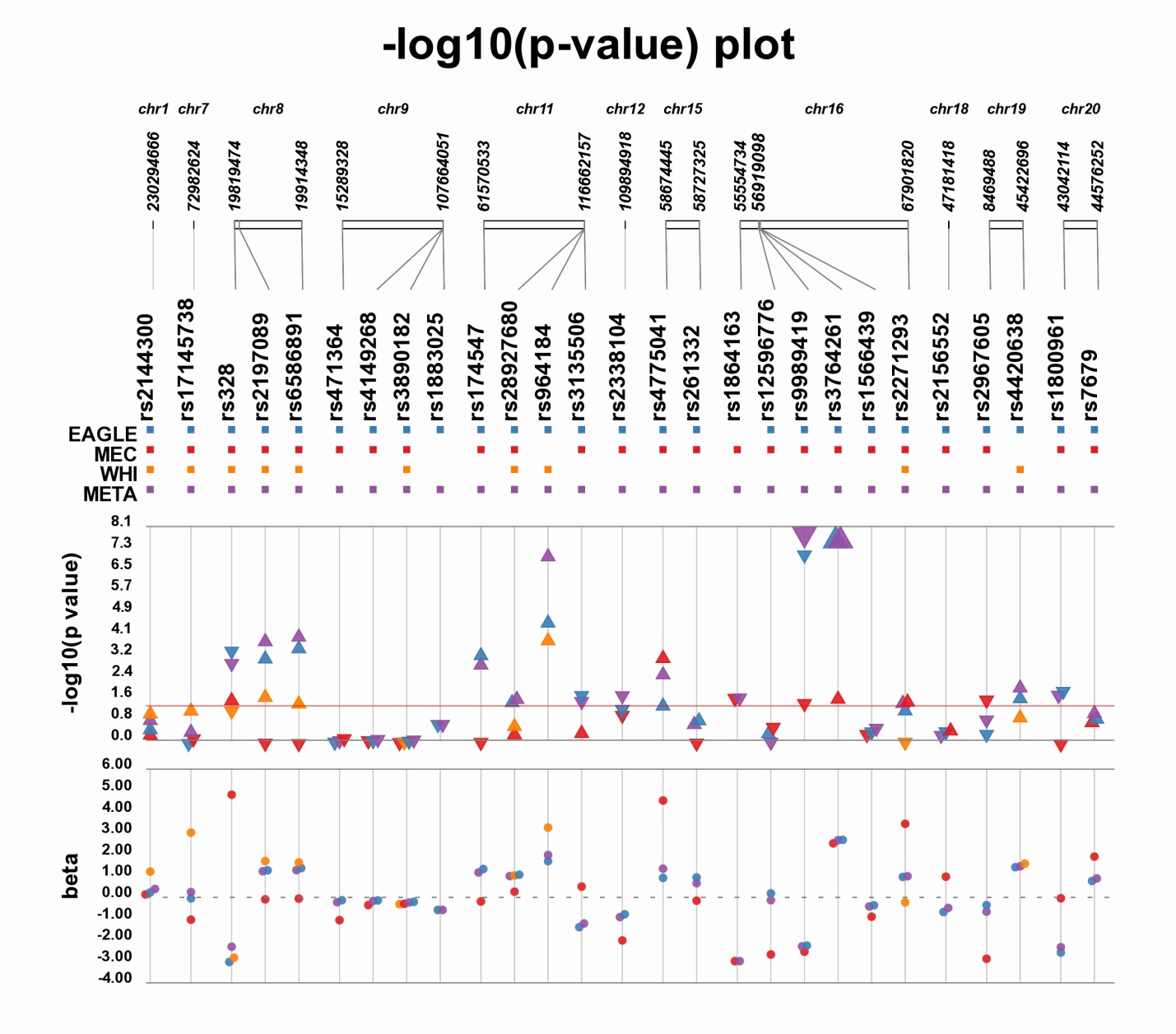

Supplement: Figure S11 — Comparison of HDL-C associations across PAGE study sites, by population. Results of tests of association for the various PAGE study sites are plotted (where available) along with meta-analysis results (META): Atherosclerosis Risk in Communities (ARIC), Coronary Artery Risk in Young Adults (CARDIA), Cardiovascular Heart Study (CHS), Epidemiologic Architecture for Genes Linked to Environment (EAGLE), Multiethnic Cohort (MEC), Women's Health Initiative (WHI), Strong Heart Community Study (SHCS), and Strong Heart Family Study (SHFS) in Arizona(AZ), Oklahoma (OK) and South Dakota (SD). Each SNP was tested for an association with HDL-C, adjusted for age and sex (Model 2), including fasting adults on lipid lowering medications. SNP location is given on the x-axis and p-values (−log10 transformed) are plotted along the y-axis. Each triangle represents a p-value for each PAGE study. PAGE study sites are color coded. Large triangles represent p-values at or smaller than genome-wide significance (p<10−8). The direction of the arrows corresponds to the direction of the beta coefficient. The exact beta coefficients are reported on the bottom panel. The significance threshold is indicated by the red bar at p = 0.05. (DOCX) [file pgen.1002138.s011.docx]
