# Supplementary material for: Genetic Determinants of Lipid Traits in Diverse Populations from the Population Architecture using Genomics and Epidemiology (PAGE) Study
Source: PLoS Genet. 2011 Jun 30;7(6):e1002138. doi: 10.1371/journal.pgen.1002138 (PMC3128106; doi:10.1371/journal.pgen.1002138)
Supplement: Table S1 — Study characteristics by PAGE study and population. Descriptive statistics for fasting (≥8 hours) adults (≥18 years of age) are expressed as percentage, median, and standard deviation (SD) for each variable. (DOCX) [file pgen.1002138.s014.docx]

**Table S1. Study characteristics by PAGE study and population**.

1. **CALiCo (ARIC)**

|  | **European Americans** | **African Americans** |
| --- | --- | --- |
| **N** | 11,178 | 3,770 |
| **% Female** | 52.93 | 62.02 |
| **Median Age**  **(SD)** | 54 years  (5.71) | 53 years  (5.84) |
| **Median BMI**  **(SD** | 26 kg/m^2^  (4.86) | 29 kg/m^2^  (6.13) |
| **Median HDL-C**  **(SD)** | 47 mg/dl  (16.74) | 52 mg/dl  (17.56) |
| **Median LDL-C**  **(SD)** | 135 mg/dl  (37.74) | 135 mg/dl  (43.18) |
| **Median TG**  **(SD)** | 115 mg/dl  (91.16) | 95 mg/dl  (76.60) |
| **% Lipid lowering medication use** | 3.41 | 1.41 |
| **% Current smokers** | 24.40 | 29.54 |
| **% Post menopausal** | 59.52 | 58.35 |
| **% Hormone use** | 20.49 | 13.73 |
| **% Previous MI** | 4.01 | 3.23 |

1. **CALiCo (CARDIA)**

|  | **European Americans** | **African Americans** |
| --- | --- | --- |
| **N** | 2,134 | 2,035 |
| **% Female** | 53.30 | 58.40 |
| **Median Age**  **(SD)** | 26 years  (3.4) | 24.5 years  (3.8) |
| **Median BMI**  **(SD)** | 23 kg/m^2^  (4.1) | 24 kg/m^2^  (5.75) |
| **Median HDL-C**  **(SD)** | 51 mg/dl  (12.97) | 53 mg/dl  (13.01) |
| **Median LDL-C**  **(SD)** | 105 mg/dl  (29.8) | 109 mg/dl  (31.85) |
| **Median TG**  **(SD)** | 66 mg/dl  (55.7) | 57 mg/dl  (36) |
| **% Lipid lowering medication use** | 0 | 0 |
| **% Current smokers** | 25.4 | 32.3 |
| **% Post menopausal** | 0 | 0 |
| **% Hormone use** | 0 | 0 |
| **% Previous MI** | 0 | 0 |

1. **CALiCo (CHS)**

|  | **European Americans** | **African Americans** |
| --- | --- | --- |
| **N** | 2,787 | 550 |
| **% Female** | 61 | 63 |
| **Median Age**  **(SD)** | 74 years  (5.15) | 72 years  (5.63) |
| **Median BMI**  **(SD)** | 26 kg/m^2^  (4.48) | 28 kg/m^2^  (5.58) |
| **Median HDL-C**  **(SD)** | 51 mg/dl  (14.18) | 55 mg/dl  (15.11) |
| **Median LDL-C**  **(SD)** | 126 mg/dl  (33.44) | 128 mg/dl  (36.13) |
| **Median TG**  **(SD)** | 128 mg/dl  (88.18) | 102 mg/dl  (58.35) |
| **% Lipid lowering medication use** | 8 | 7 |
| **% Current smokers** | 9 | 14 |
| **% Post menopausal** | 100 | 100 |
| **% Hormone use** | 9 | 5 |
| **% Previous MI** | 11 | 8 |

1. **EAGLE**

|  | **European Americans** | **African Americans** | **Mexican Americans** |
| --- | --- | --- | --- |
| **N** | 3,909 | 1,896 | 2,361 |
| **% Female** | 53.83 | 55.89 | 50.76 |
| **Median Age**  **(SD)** | 51 years  (19.69) | 39 years  (16.65) | 40 years  (17.52) |
| **Median BMI**  **(SD)** | 27 kg/m^2^  (5.84) | 28 kg/m^2^  (6.96) | 28 kg/m^2^  (5.51) |
| **Median HDL-C**  **(SD)** | 48 mg/dl  (15.76) | 51 mg/dl  (16.82) | 46 mg/dl  (13.35) |
| **Median LDL-C**  **(SD)** | 125 mg/dl  (36.08) | 118 mg/dl  (38.75) | 119 mg/dl  (33.54) |
| **Median TG**  **(SD)** | 123  (124.53) | 85 mg/dl  (75.85) | 128 mg/dl  (123.47) |
| **% Lipid lowering medication use** | 4.61 | 2.15 | 2.37 |
| **% Current smokers** | 261.0 | 35.30 | 20.90 |
| **% Post menopausal** | 18.77 | 9.36 | 10.01 |
| **% Hormone use** | 4.99 | 3.56 | 2.07 |
| **% Previous MI** | 5.17 | 3.29 | 2.28 |

1. **MEC**

|  | **European Americans** | **African Americans** | **Mexican Americans** | **Native Hawaiians** | **Japanese** |
| --- | --- | --- | --- | --- | --- |
| **N** | 317 | 552 | 299 | 87 | 576 |
| **% Female** | 38.85 | 18.08 | 32.78 | 48.86 | 38.99 |
| **Median Age (SD)** | 67 years  (8.01) | 69 years  (7.13) | 68 years  (6.92) | 62 years  (7.02) | 70 years  (8.28) |
| **Median BMI (SD)** | 26 kg/m^2^  (5.11) | 27 kg/m^2^  (4.71) | 27 kg/m^2^  (4.54) | 28 kg/m^2^  (5.32) | 24 kg/m^2^  (3.61) |
| **Median HDL-C (SD)** | 53 mg/dl  (15.57) | 50 mg/dl  (14.69) | 48 mg/dl  (12.97) | 48 mg/dl  (13.66) | 54 mg/dl  (14.92) |
| **Median LDL-C (SD)** | 116 mg/dl  (32.71) | 123 mg/dl  (41.93) | 118 mg/dl  (36.65) | 108 mg/dl  (27.16) | 111 mg/dl  (34.71) |
| **Median TG (SD)** | 109 mg/dl  (61.27) | 103 mg/dl  (54.47) | 136 mg/dl  (70.37) | 130 mg/dl  (69.85) | 126 mg/dl  (69.31) |
| **% Lipid lowering medication use** | 28.39 | 20.8 | 24.08 | 27.27 | 38.3 |
| **% Current smokers** | 6.31 | 21.05 | 15.36 | 9.09 | 8.7 |
| **% Post menopausal** | 76.32 | 62.22 | 71.43 | 67.5 | 69.16 |
| **% Hormone use** | 67.8 | 50 | 51.04 | 57.14 | 61.26 |
| **% Previous MI** | 5.67 | 9.95 | 8.36 | 6.82 | 5.72 |

1. **CALiCo (SHFS)**

|  | **American Indians (Arizona)** | **American Indians**  **(Oklahoma)** | **American Indians**  **(the Dakotas)** |
| --- | --- | --- | --- |
| **N** | 1,191 | 1,196 | 1,178 |
| **% Female** | 62 | 59 | 59 |
| **Median Age**  **(SD)** | 36 years  (15.96) | 42 years  (17.29) | 38 years  (17.08) |
| **Median BMI**  **(SD)** | 35 kg/m^2^  (8.80) | 30 kg/m^2^  (6.89) | 29 kg/m^2^  (6.83) |
| **Median HDL-C**  **(SD)** | 45 mg/dl  (14.11) | 50 mg/dl  (15.38) | 49 mg/dl  (13.74) |
| **Median LDL-C**  **(SD)** | 93 mg/dl  (25.91) | 97 mg/dl  (30.41) | 98 mg/dl  (30.92) |
| **Median TG**  **(SD)** | 139 mg/dl  (134.80) | 144 mg/dl  (170.62) | 126 mg/dl  (201.74) |
| **% Lipid lowering medication use** | 3.91 | 5.14 | 6.07 |
| **% Current smokers** | 25.06 | 33.22 | 42.22 |
| **% Post menopausal** | 15.82 | 21.96 | 15.94 |
| **% Hormone use** | 2.41 | 9.44 | 4.97 |
| **% Previous MI** | -- | -- | -- |

1. **CALiCo (SHS-C)**

|  | **American Indians (Arizona)** | **American Indians**  **(Oklahoma)** | **American Indians**  **(the Dakotas)** |
| --- | --- | --- | --- |
| **N** | 950 | 943 | 939 |
| **% Female** | 68 | 60 | 59 |
| **Median Age**  **(SD)** | 54 years  (7.88) | 56 years  (8.19) | 55 years  (7.91) |
| **Median BMI**  **(SD)** | 32 kg/m^2^  (7.07) | 30 kg/m^2^  (6.04) | 29 kg/m^2^  (5.46) |
| **Median HDL-C**  **(SD)** | 43 mg/dl  (12.31) | 44 mg/dl  (13.61) | 44 mg/dl  (14.41) |
| **Median LDL-C**  **(SD)** | 97 mg/dl  (29.43) | 109 mg/dl  (31.20) | 113 mg/dl  (32.29) |
| **Median TG**  **(SD)** | 121 mg/dl  (138.42) | 122 mg/dl  (120.59) | 113 mg/dl  (198.42) |
| **% Lipid lowering medication use** | -- | -- | -- |
| **% Current smokers** | 18.34 | 32.63 | 47.79 |
| **% Post menopausal** | -- | -- | -- |
| **% Hormone use** | 2.41 | 9.44 | 4.97 |
| **% Previous MI** | 0.83 | 2.94 | 3.37 |

**h) WHI**

|  | **European Americans** | **African Americans** | **Hispanics** | **Asian/Pacific Islander** | **American Indians** |
| --- | --- | --- | --- | --- | --- |
| **N** | 4,688 | 1,840 | 762 | 359 | 113 |
| **% Female** | 100 | 100 | 100 | 100 | 100 |
| **Median Age (SD)** | 67 years  (6.86) | 60 years  (7.13) | 60 years  (6.67) | 66 years  (7.19) | 59 years  (6.72) |
| **Median BMI (SD)** | 27 kg/m^2^  (6.53) | 30 kg/m^2^  (8.12) | 28 kg/m^2^  (5.79) | 24 mg/k^2^  (4.10) | 29 kg/m^2^  (5.89) |
| **Median HDL-C (SD)** | 55 mg/dl  (15.26) | 55 mg/dl  (14.36) | 51 mg/dl  (14.34) | 59 mg/dl  (16.28) | 52 mg/dl  (13.53) |
| **Median LDL-C (SD)** | 140 mg/dl  (34.31) | 137 mg/dl  (38.46) | 124 mg/dl  (38.22) | 124 mg/dl  (29.57) | 124 mg/dl  (31.70) |
| **Median TG (mg/dl)** | 140 mg/dl  (114.12) | 110 mg/dl  (55.73) | 151 mg/dl  (90.38) | 140 mg/dl  (87.98) | 140 mg/dl  (64.34) |
| **% Lipid lowering medication use** | 9.3 | 7.3 | 7.4 | 16.2 | 4.4 |
| **% Current smokers** | 8.1 | 10.3 | 6.4 | 2.3 | 8.9 |
| **% Post menopausal** | 100 | 100 | 100 | 100 | 100 |
| **% Hormone use** | 28.2 | 22.3 | 31.8 | 40.7 | 41.6 |
| **% Previous MI** | 1.6 | 1.5 | 0 | 1.1 | 0.9 |
