# Supplementary material for: Genetic Determinants of Lipid Traits in Diverse Populations from the Population Architecture using Genomics and Epidemiology (PAGE) Study
Source: PLoS Genet. 2011 Jun 30;7(6):e1002138. doi: 10.1371/journal.pgen.1002138 (PMC3128106; doi:10.1371/journal.pgen.1002138)
Supplement: Table S2 — List of candidate gene and GWAS-identified SNPs targeted for genotyping in PAGE. For each SNP (denoted by rs number), we list the chromosomal and genomic location, the putative function of the SNP (based on SNP location) and the nearest gene, the number of PAGE studies that genotyped the SNP, the trait associated with the SNP based on the literature, the effect allele and effect size based on the literature, and the reference for these data. (DOC) [file pgen.1002138.s015.doc]

**Table S2. List of candidate gene and GWAS-identified SNPs targeted for genotyping in PAGE.**  For each SNP (denoted by rs number), we list the chromosomal and genomic location, the putative function of the SNP (based on SNP location) and the nearest gene, the number of PAGE studies that genotyped the SNP, the trait associated with the SNP based on the literature, the effect allele and effect size based on the literature, and the reference for these data.

| **SNP** | **Chr.** | **Build 37 location (bp)** | **Location (Function)** | **Nearest Gene of Interest** | **# PAGE Studies*** | **Previously Associated Trait** | **Effect Allele** | **Effect Size^‡^ (mg/dl)** | **Reference** |
| --- | --- | --- | --- | --- | --- | --- | --- | --- | --- |
| rs11206510 | 1 | 55495789 | US | *PCSK9* | 4 | LDL-C | T | 3.04 | Willer et al 2008 [79] |
| rs11591147 | 1 | 55505397 | Exon (Missense) | *PCSK9* | 3 | LDL-C | T | -17.1 | Kathiresan et al 2009 [80] |
| rs1748195 | 1 | 63049343 | US | *ANGPTL3* | 2 | TG | C | 7.12 | Willer et al 2008 [79] |
| rs646776 | 1 | 109818280 | DS/DS/DS | *CELSR2*/*PSRC*/  *SORT1* | 4 | LDL-C | A | 6.18 | Kathiresan et al 2008 [81] |
| rs599839 | 1 | 109821916 | DS/DS/DS | *CELSR2*/*PSRC*/  *SORT1* | 3 | LDL-C | A | 5.48 | Willer et al 2008 [79] |
| rs2144300 | 1 | 230294666 | Intron | *GALNT2* | 4 | HDL-C | T | 1.11 | Willer et al 2008 [79] |
| rs693 | 2 | 21231945 | Exon (Synon) | *APOB* | 4 | LDL-C | T | 2.44 | Willer et al 2008 |
| rs562338 | 2 | 21288071 | US | *APOB* | 4 | LDL-C | T | -4.89 | Willer et al 2008 |
| rs754523 | 2 | 21311441 | US | *APOB* | 4 | LDL-C | T | -2.78 | Willer et al 2008 |
| rs1260326 | 2 | 27730690 | Exon (Missense) | *GCKR* | 2 | TG | T | 8.76 | Teslovich et al 2010 [82] |
| rs780094 | 2 | 27740987 | Intron | *GCKR* | 3 | TG | A | 8.59 | Willer et al 2008 |

| rs6544713 | 2 | 44073631 | Intron | *ABCG8* | 4 | LDL-C | T | 5.1 | Kathiresan et al 2009 |
| --- | --- | --- | --- | --- | --- | --- | --- | --- | --- |
| rs12654264 | 5 | 74648353 | Intron | *HMGCR* | 4 | LDL-C | A | -3.86 | Kathiresan et al 2008 |
| rs1501908 | 5 | 156397919 | US | *TIMD4* | 3 | LDL-C | G | 2.38 | Kathiresan et al 2009 |
| rs17145738 | 7 | 72982624 | DS | *MLXIPL* | 4 | HDL-C | T | 0.57 | Teslovich et al 2010 |
|  |  |  |  |  |  | TG | T | -9.32 | Teslovich et al 2010 |
| rs328 | 8 | 19819474 | Exon (Nonsense) | *LPL* | 4 | HDL-C | C | -2.62 | Kathiresan et al 2008 |
|  |  |  |  |  |  | TG | C | 19.47 | Kathiresan et al 2008 |
| rs2197089 | 8 | 19826123 | DS | *LPL* | 4 | HDL-C | T | 1.38 | Willer et al 2008 |
|  |  |  |  |  |  | TG | T | -3.38 | Willer et al 2008 |
| rs6586891 | 8 | 19914348 | DS | *LPL* | 4 | HDL-C | A | 1 | Willer et al 2008 |
| rs2954029 | 8 | 126560154 | DS | *TRIB1* | 2 | TG | A | 5.64 | Teslovich et al 2010 |
| rs471364 | 9 | 15289328 | Intron | *TTC39B* | 3 | HDL-C | A | 1.2 | Kathiresan et al 2009 |
| rs4149268 | 9 | 107647220 | Intron | *ABCA1* | 4 | HDL-C | A | -0.82 | Willer et al 2008 |
| rs3890182 | 9 | 107647405 | Intron | *ABCA1* | 4 | HDL-C | A | -1.54 | Kathiresan et al 2008 |
| rs1883025 | 9 | 107664051 | Intron | *ABCA1* | 2^§^ | HDL-C | A | -0.94 | Teslovich et al 2010 |
| rs174547 | 11 | 61570533 | Intron | *FADS1* | 4 | HDL-C | T | 1.35 | Kathiresan et al 2009 |
|  |  |  |  |  |  | TG | T | 5.46 | Kathiresan et al 2009 |

| rs28927680 | 11 | 116618823 | DS/US/DS/DS | *APOA1/C3/A4/A5* gene cluster | 4 | HDL-C | G | 2.01 | Kathiresan et al 2008 | |
| --- | --- | --- | --- | --- | --- | --- | --- | --- | --- | --- |
|  |  |  |  |  |  | TG | G | -16.95 | Teslovich et al 2010 | |
| rs964184 | 11 | 116648667 | DS/US/DS/DS | *APOA1/C3/A4/A5* gene cluster | 2 | HDL-C | C | 1.5 | Teslovich et al 2010 | |
|  |  |  |  |  |  | TG | C | -27.3 | Kathiresan et al 2009 | |
| rs3135506 | 11 | 116662157 | Exon  (Missense) | *APOA1/C3/A4/A5* gene cluster | 3 | HDL-C | C | -2.65 | Lu et al  2008 [83] | |
|  |  |  |  |  |  | TG | C | 1.13# | Ariza et al  2010 [84] | |
| rs2338104 | 12 | 109894918 | DS | *MMAB*-MVK | 3 | HDL-C | C | -0.48 | Willer et al 2008 | |
| rs2650000 | 12 | 121388712 | US | *HNF1A* | 3 | LDL-C | T | 2.38 | Kathiresan et al 2009 | |
| rs4775041 | 15 | 58674445 | US | *LIPC* | 4 | HDL-C | C | 1.38 | Willer et al 2008 | |
|  |  |  |  |  |  | TG | C | 3.62 | Willer et al 2008 | |
| rs261332 | 15 | 58727325 | Intron | *LIPC* | 3 | HDL-C | A | 1.41 | Willer et al 2008 | |
| rs1800775 | 16 | 555552737 | US | *CETP* | 3 | HDL-C | SNP failed genotyping at all PAGE sites that attempted genotyping | | | |
| rs1864163 | 16 | 55554734 | Intron | *CETP* | 2 | HDL-C | A | -4.12 | | Willer et al 2008 |
| rs12596776 | 16 | 56919098 | US | *CETP* | 2 | HDL-C | C | -1.26 | | Willer et al 2008 |
| rs9989419 | 16 | 56984889 | US | *CETP* | 4 | HDL-C | A | -1.72 | | Willer et al 2008 |
| rs3764261 | 16 | 56993074 | US | *CETP* | 4 | HDL-C | T | 3.47 | | Willer et al 2008 |
| rs1566439 | 16 | 57024412 | DS | *CETP* | 2 | HDL-C | A | -0.93 | | Willer et al 2008 |
| rs2271293 | 16 | 67901820 | DS | *LCAT* | 4 | HDL-C | A | 1.05 | | Kathiresan et al 2009 |
| rs2156552 | 18 | 47181418 | DS | *LIPG* | 4 | HDL-C | T | -1.2 | | Willer et al 2008 |
| rs2967605 | 19 | 8469488 | DS | *ANGPTL4* | 3 | HDL-C | A | -1.8 | | Kathiresan et al 2009 |
| rs6511720 | 19 | 11202056 | Intron | *LDLR* | 4 | LDL-C | T | -6.99 | | Teslovich et al 2010 |
| rs2228671 | 19 | 11210662 | Exon  (Synon) | *LDLR* | 2 | LDL-C | T | -0.14^‡^ | | Aulchenko et al 2009 [85] |
| rs16996148 | 19 | 19658222 | DS/DS/DS | *CILP2*/*PBX4*/  *NCAN* | 4 | LDL-C | T | -3.32 | | Willer et al 2008 |
|  |  |  |  |  |  | TG | T | -6.1 | | Willer et al 2008 |
| rs4803750 | 19 | 45247627 | US | *BCL3* | 2 | LDL-C | A | 10.9† | | Sandhu et al 2008 [86] |
| rs10402271 | 19 | 45329214 | US/US/US | *APOE/C1/C4*  gene cluster | 2 | LDL-C | T | -2.62 | | Willer et al 2008 |
| rs4420638 | 19 | 45422696 | DS/DS/US | *APOE/C1/C4*  gene cluster | 4 | HDL-C | A | 1.06 | | Teslovich et al 2010 |
|  |  |  |  |  |  | LDL-C | A | -7.14 | | Teslovich et al 2010 |
| rs2075650 | 19 | 50087459 | Intron | *TOMM40* | 2 | LDL-C | A | -0.16^‡^ | | Aulchenko et al 2009 |
| rs429358 | 19 | 50103781 | Exon (Missense) | *APOE* | 2 | LDL-C | SNP failed genotyping at all PAGE sites that attempted genotyping | | | |
| rs7412 | 19 | 50103919 | Exon (Missense) | *APOE* | 2 | LDL-C | SNP failed genotyping at all PAGE sites that attempted genotyping | | | |
| rs6102059 | 20 | 39228784 | DS | *MAFB* | 3 | LDL-C | T | -2.04 | | Kathiresan et al 2009 |
| rs1800961 | 20 | 43042114 | Exon | *HNF4A* | 3 | HDL-C | T | -1.88 | | Teslovich et al 2010 |
| rs7679 | 20 | 44576252 | US | *PLTP* | 3 | HDL-C | T | 1.05 | | Kathiresan et al 2009 |
|  |  |  |  |  |  | TG | T | -6.37 | | Kathiresan et al 2009 |

Abbreviations: Base-pair (bp), Upstream (US), Downstream (DS), Synonymous (Synon)

^*^Number of PAGE studies that genotyped the SNP. PAGE studies include CALiCo, EAGLE, MEC, and WHI.

^§^SNP failed genotyping on the BeadXpress at one PAGE site (WHI); therefore, only data from one PAGE site was available for analysis (EAGLE using Sequenom).

^‡^Effect size (beta) is reported in s.d. (standard deviation) units

†Effect size originally expressed in mmol/L

#Ariza et al collapsed heterozygotes and homozygotes for the minor allele into the same bin and compared them to the referent (homozygotes for the major allele) to estimate the genetic effect size
