# Supplementary material for: Genetic Determinants of Lipid Traits in Diverse Populations from the Population Architecture using Genomics and Epidemiology (PAGE) Study
Source: PLoS Genet. 2011 Jun 30;7(6):e1002138. doi: 10.1371/journal.pgen.1002138 (PMC3128106; doi:10.1371/journal.pgen.1002138)
Supplement: Text S1 — Study descriptions. (DOCX) [file pgen.1002138.s016.docx]

**Text S1**

**Descriptions of Participants, Data Collection, and Genotyping by Study:**

1. **Causal Variants Across the Life Course (CALiCo).**  CALiCo is a consortium of six demographically diverse population based studies and a central laboratory. This network contributes a maximum of approximately 58,000 men and women ranging in age from childhood to older adulthood. Four CALiCo studies are involved in this analysis:
   1. **Atherosclerosis Risk in Communities (ARIC) Study.** The ARIC study is a multi-center prospective investigation of atherosclerotic disease in a predominantly bi-racial population. White and African American men and women aged 45-64 years at baseline were recruited from four communities: Forsyth County, North Carolina; Jackson, Mississippi; suburban areas of Minneapolis, Minnesota; and Washington County, Maryland [1]. A total of 15,792 individuals participated in the baseline examination in 1987-1989, with follow-up examinations in approximate 3-year intervals, during 1990-1992, 1993-1995, and 1996-1998. After the institutional review board at every participating university approved the ARIC Study protocol, written informed consent was obtained from each participant.

***Data Collection:*** Body mass index was calculated as the ratio of weight in kilograms to height in meters squared. Current smoking was defined by “Do you now smoke cigarettes?” Diabetes status at baseline (yes/no) was based on either fasting plasma glucose levels ≥126 mg/dL, non-fasting plasma glucose ≥200 mg/dL, anti-diabetic medication use within two weeks of the baseline interview, or self-report of a physician diagnosis of diabetes. Women reported their age at menopause, if applicable, and hormone therapy replacement use at baseline. Women were either classified as pre-menopausal, peri-menopausal, natural post-menopausal, and surgical post-menopausal [2]. Race and lipid-lowering medication use were self-reported. Previous myocardial infarction (MI) was indicated by self-report of physician-diagnosed MI or silent MI identified by electrocardiography. Fasting blood was drawn while the participant was seated from an antecubital vein into tubes containing EDTA, and plasma was obtained by centrifugation at 4°C and stored at −70°C until analysis. All samples were sent to the ARIC Central Lipid Laboratory for processing. Triglycerides were determined by enzymatic methods [3] using the Cobas Bioanalyzer (Roche). Plasma HDL-C levels were measured using an enzymatic cholesterol assay using dextran-magnesium precipitation [4]. The Friedewald equation was used to calculate LDL-C in those with triglyceride levels under 400 mg/dl [5]. Blood chemistries were performed at the Central Chemistry Laboratory of the University of Minnesota, and blood lipid analyses were performed at the University of Texas, Houston.

***Genotyping:*** ARIC Study samples were genotyped using two approaches: *de novo* genotyping with TaqMan 6.0 (Applied Biosystems) and accessing previous genome-wide association study data from the Affymetrix Genome-Wide Human SNP Array 6.0 (Santa Clara, California). For the *de novo* genotyping data, the genotyping calls were made using the Applied Biosystem Autocaller 3.1 software. Internal QC's were included on every plate and across the full genotyping sample sets. QC genotypes were examined for consistency within the SNP genotyped. Other criteria include: 1) Internal genomic DNA, which is examined for replication across the study set; 2) Fingerprint blanks, which are used to identify the plate and verify cross-contamination and or sample error; 3) Genomic DNA pools to examine consistency of genotypes and plate validation; 4) No template controls (NTC), which serve as a background detector and review of any reagent problems; and 5) Autocaller confidence score, which determines percent genotyping call reliability. All SNPs are tested for departure from Hardy-Weinberg Equilibrium (HWE), and SNPs with HWE χ2>3.84 in unrelated cohorts (excluding duplicates) were excluded. SNP genotype data was also obtained from previous GWAS data. Genotyping was conducted using the Affymetrix Genome-Wide Human SNP Array 6.0 (Santa Clara, California). Sample exclusion criteria included discordant with previous genotype data, genotypic and phenotypic sex mismatch, suspected first-degree relative of an included individual based on genotype data (n=297), genetic outlier as assessed by Identity by State (IBS) using PLINK [5,6], and >8 SD along any of the first 10 principal components in EIGENSTRAT [7] with 5 iterations. Autosomal SNPs were used for imputation after exclusion of SNPs with HWE deviation p<5 x 10‾^5^, call rate <95%, or MAF<1%.

- 1. **The Coronary Artery Risk in Young Adults (CARDIA) Study.** CARDIA is a multicenter longitudinal study of the development and determinants of cardiovascular disease in 5,115 young adults initially aged 18 to 30 years from 1985 to 1986. Black and white adults were recruited from four U.S. cities (Birmingham, Alabama; Chicago, Illinois; Minneapolis, Minnesota; and Oakland, California) with population-based samples approximately balanced within center by sex, age (18 to 24 or 25 to 30 years), race (white or black), and education (high school graduate or less or greater than high school graduate). Participants have been reexamined 2, 5, 7, 10, 15, and 20 years after baseline; and retention rates across examinations were 91%, 86%, 81%, 79%, 74%, and 72%, respectively. Further details of study recruitment and design are available [8]. All participants provided written informed consent at each examination, and institutional review boards from each field center and the coordinating center approved the study annually.

***Data Collection:*** Each participant’s age, race, and sex were self-reported during the recruitment phase and verified during the baseline clinic visit. Structured interviews or self-administered questionnaires were used to collect information on demographic characteristics, lifestyle habits, physical activity, and medical history. The detailed methods, instruments and quality control procedures have been previously described [8]. Body weight was measured to the nearest 0.1 kg, using a calibrated scale, with the participant in light clothing without shoes. Height was measured to the nearest 0.5 cm with a vertical ruler. Body mass index (BMI) was computed as body weight / height^2^ (kg/m^2^). Blood samples were drawn after an overnight fast. Total plasma cholesterol, triglycerides, HDL-, and LDL-cholesterol were measured according to standardized methods. Triglycerides were measured enzymatically within 6 weeks of collection [9]. HDL-C was determined after precipitation with dextran sulfate/magnesium chloride of lipoproteins containing low-density lipoprotein cholesterol [4].

***Genotyping:*** Genotypes were obtained from the Central Texas laboratory using TaqMan (as described above for ARIC).

- 1. **The Cardiovascular Heart Study (CHS).** The CHS is a population-based longitudinal study of risk factors for cardiovascular disease in adults 65 years of age or older, recruited at four field centers (Forsyth County, North Carolina; Sacramento County, California; Washington County, Maryland; Pittsburgh, Pennsylvania) [10]. Overall, 5,201 predominantly white individuals were recruited in 1989-1990 from random samples of Medicare eligibility lists, followed by an additional 687 African Americans recruited in 1992-1993 (total n=5,888).

***Data Collection:*** CHS participants completed standardized clinical examinations and questionnaires at study enrollment and at nine annual follow-up visits. Height and weight were measured at the baseline examination. Current smoking status was self-reported at baseline. LDL cholesterol, HDL cholesterol, and triglycerides were measured under fasting conditions by enzymatic methods at a central laboratory [11]. Diabetes was defined as history of diabetes, use of hypoglycemic agent or insulin, or fasting glucose 126 mg/dL. All women in CHS are postmenopausal. Race/ethnicity was based on self-report. MI was defined as evolving Q-wave MI or cardiac pain plus abnormal enzymes and either an evolving ST-T pattern or new left bundle branch block.

***Genotyping:*** DNA was extracted from blood samples drawn on all participants at their baseline examination. Like ARIC, the CHS SNP genotypes were also obtained from two sources. First, SNP genotyping data was conducted in the Houston central lab using TaqMan (please see details above provided for the ARIC study). The second source of genotyping data from CHS was performed at the General Clinical Research Center's Phenotyping/Genotyping Laboratory at Cedars-Sinai using the Illumina 370CNV BeadChip system. Genotypes were called using the Illumina BeadStudio software as above. The following exclusions were applied to identify a final set of 306,655 autosomal SNPs: call rate < 97%, HWE p < 1x10^-5^, > 1 duplicate error or Mendelian inconsistency (for reference CEPH trios), heterozygote frequency = 0, SNP not found in dbSNP.  A total of 1,908 persons were excluded from the GWAS study sample due to the presence at study baseline of coronary heart disease, congestive heart failure, peripheral vascular disease, valvular heart disease, stroke, or transient ischemic attack [12].

- 1. **The Strong Heart Study (SHS).** SHS is composed of both a community-based and family-based study [13]. The Strong Heart Community Study (SHCS) is a community-based study of CVD and its risk factors. The family component of the study, the SHFS, began in 1998 (phase III) with a pilot study that recruited and examined at least 300 members [14]. In phase IV of the SHFS, an additional ~900 family members were recruited from each center yielding sample sizes of more than 1,200 participants at each site. In all centers, some individuals are descended from more than one tribe and/or from non-Indian ancestors. Informed consent was obtained from all participants.

***Data Collection:*** During the clinic visit for SHFS participants, a personal interview and physical exam of family members were performed. Tobacco exposure was quantified using standardized questionnaires. Anthropometric measures of height and weight were recorded and used to estimate BMI.

***Genotyping:*** Genotypes were obtained from the Central Texas laboratory using TaqMan (as described above for ARIC).

1. **Epidemiologic Architecture for Genes Linked to Environment (EAGLE).** The EAGLE study accesses DNA samples and data collected for the National Health and Nutrition Examination Surveys (NHANES) by the National Center on Health Statistics (NCHS) at the Centers for Disease Control and Prevention (CDC). NHANES is a collection of diverse, population-based cross-sectional surveys of non-institutionalized Americans regardless of health status at the time of ascertainment. NHANES is considered a complex survey given that specific age groups (such as the elderly) and racial/ethnic groups (non-Hispanic blacks and Mexican-Americans) are oversampled. The NHANES data accessed for this work includes phase 2 of NHANES III (collected between 1991 and 1994), NHANES 1999-2000, and NHANES 2000-2001. Collectively, these surveys contain 14,998 DNA samples linked to demographic, health, and lifestyle data. Participants were consented by the CDC at the time of the survey and sample collection, and consent included the storage of data and biological specimens such as blood for future research [15]. The present study was approved by the CDC Ethics Review Board. Because the study investigators did not have access to personal identifiers, this study was considered non-human subjects research by the Vanderbilt University Internal Review Board.

***Data Collection:*** In EAGLE/NHANES, race/ethnicity is self-described and was categorized as non-Hispanic white, non-Hispanic black, Mexican-American, and others. Serum HDL-C, triglycerides, and total cholesterol were measured using standard enzymatic methods. LDL-C was calculated using the Friedewald equation, with missing values assigned for samples with triglyceride levels greater than 400 mg/dl. Body mass index was calculated from height and weight measured in the Mobile Examination Center by CDC medical personnel. Current smoking was defined by “do you smoke cigarettes now?” or cotinine levels > 15ng/ml. Post-menopausal status was defined as a woman >60 years of age answering “no” to “have you had a period or regular periods in the past 12 months” or as a woman with bilateral oophorectomy. Current hormone use in NHANES III was defined as “yes” to “have you ever taken or used estrogen or female hormones in any form? Include pills, vaginal cream, suppositories, injections, or skin patches” and “still taking” to “how many months ago did you stop taking or using the estrogen or female hormones?”. In NHANES 1999-2002, hormone use is defined as “yes” to “ever used female hormones?” and “yes” to “ever take estrogen/progestin?” Participants were considered to have type 2 diabetes if they answered “yes” to “Ever been told you have sugar/diabetes?” and “Are you now taking insulin?” or if they had fasting blood glucose levels >126 mg/dL. Previous myocardial infarction was defined by “Doctor ever told you had a heart attack?” (NHANES III) or “Ever told you had heart attack” (NHANES 1999-2002). Lipid lowering medication use was defined by “take prescribed med to lower cholesterol?” (NHANES III) and “now taking prescribed medicine to lower blood pressure” (NHANES 1999-2002).

***Genotyping:*** In EAGLE, rs693 (*APOB*), rs673548 (*APOB*), rs2228671 (*LDLR*), and rs6511720 (*LDLR*) genotyping in NHANES III was performed using the Illumina GoldenGate assay (as part of a custom 384 OPA) by the Center for Inherited Disease Research (CIDR) through the National Heart Lung and Blood Institute’s Resequencing and Genotyping Service. For rs3890182 (*ABCA1*), rs3135506 (*APOA5*), rs1800775 (*CETP*), rs1260326 (*GCKR*), rs1323432 (*GRIN3A*), rs12654264 (*HMGCR*), rs1800588 (*LIPC*), rs1529729 (*LDLR*), rs328 (*LPL*), and rs11591147 (*PCSK9*) in NHANES III, we accessed existing data [16] in the Genetic NHANES database. Genotyping was performed for the remaining EAGLE SNPs in NHANES III (n=7,159) and NHANES 1999-2002 (n=7,839) using Sequenom or Illumina BeadXpress. Genotyping was performed in Vanderbilt DNA Resources Core. In addition to genotyping experimental NHANES samples, we genotyped blind duplicates provided by CDC and HapMap controls (n=360). All EAGLE SNPs reported here passed CDC quality control metrics and are available for secondary analyses through NCHS/CDC. All statistical analyses were conducted remotely in SAS v9.2 (SAS Institute, Cary, NC) using the Analytic Data Research by Email (ANDRE) portal of the CDC Research Data Center in Hyattsville, MD.

1. **Multiethnic Cohort (MEC).** The MEC is a population-based prospective cohort study consisting of 215,251 men and women, and comprises mainly five self-reported racial/ethnic populations: African Americans, Japanese Americans, Latinos, Native Hawaiians and European Americans [17]. The MEC was designed to provide prospective data on exposures and biomarkers potentially involved in cancer initiation and progression across groups with distinct cultural and dietary patterns. Between 1993 and 1996, adults between 45 and 75 years old were enrolled by completing a 26-page, self-administered questionnaire asking detailed information about dietary habits, demographic factors, level of education, personal behaviors, and history of prior medical conditions (e.g. diabetes). Between 1995 and 2004, blood specimens were collected from ~67,000 MEC participants at which time a short questionnaire was administered to update certain exposures, and collect current information about medication use. Study protocols and consent forms were approved by the institutional review boards at all participating institutions.

***Data Collection:*** MEC baseline questionnaires queried ever smokers (defined as >20 packs in lifetime) about the average number of cigarettes smoked per day, duration in years, and years since quitting if not currently smoking. **S**elf-reported height and weight were used to calculate baseline BMI. Medication use and fasting information was collected at the time of blood collections. LDL, HDL, and triglycerides concentrations were measured using standard clinical assays.

***Genotyping:*** Genotyping of 43 SNPs was conducted by the OpenArray platform (Life Technologies, Carlsbad, CA) at the Cancer Research Center in Hawaii following the recommended protocol. For all SNPs genotype call rates were >90% and individual call rates were >90%. Concordance rates between duplicate samples were 100%. HWE was examined and p-values were >0.01 in at least 4 of the 5 ethnic groups.

1. **Women’s Health Initiative (WHI).** WHI is a long-term national health study that focuses on strategies for preventing heart disease, breast and colorectal cancer and fracture in postmenopausal women. A total of 161, 838 women aged 50–79 yrs old were recruited from 40 clinical centers in the US between 1993 and 1998 [18]. WHI consists of an observational study, two clinical trials of postmenopausal hormone therapy (estrogen alone or estrogen plus progestin), a calcium and vitamin D supplement trial, and a dietary modification trial. Trial exclusion criteria have been described previously [19]. Study protocols and consent forms were approved by the institutional review boards at all participating institutions. A subset of 21,000 WHI women were selected for genotyping and inclusion in these PAGE analyses, of those, approximately 8,000 have at least one baseline lipid measurement available. Women were selected based on self-reported history of disease, incident event outcomes, DNA availability and consent, and racial/ethnic diversity.

***Data Collection:*** Self-reported demographic, lifestyle and general health characteristics (current smoking, history of myocardial infarction, type 2 diabetes) were collected at baseline as described previously [19]. BMI was calculated from measured weight and height at time of enrollment. Baseline medication use (lipid-lowering and hormone replacement medications) was ascertained using a computer-driven medication inventory system at the first screening visit. Race/ethnicity was self reported as one of white, black, Hispanic, Asian/Pacific Islander, Native American, or other (this last category was not genotyped for PAGE). Additional self-reported race/ethnicity data were available for a subset of participants and were used to subset Asian/Pacific Islanders into East Asians and Pacific Islanders. Fasting LDL, HDL, and triglycerides concentrations were measured using standard clinical assays in a variety of core and ancillary WHI studies; measurements were normalized to correct for laboratory and study effect.

***Genotyping:*** In WHI, 20 lipids SNPs were genotyped at the Translational Genomics Research Institute (TGen) (Phoenix, AZ) on Illumina’s BeadXpress Reader using Illumina’s Veracode GoldenGate genotyping assay, following the manufacturer’s recommended protocol ([www.illumina.com](http://www.illumina.com)). Study protocol included calculation of concordance rates among duplicates, genotyping of HapMap samples, re-genotyping of failing samples, and other extensive QA procedures. One HDL SNP, rs1883025, did not pass quality control and was excluded from analyses; all other genotyping data reported here passed QA with individual and SNP call rates exceeding 95% and 97%, respectively.

**References**

1. The ARIC Investigators (1989) The Atherosclerosis Risk in Communities (ARIC) Study: design and objectives. Am J Epidemiol 129: 687-702.

2. Luoto R, Sharrett AR, Schreiner P, Sorlie PD, Arnett D, et al. (2000) Blood pressure and menopausal transition: the Atherosclerosis Risk In Communities study (1987-95). Journal of Hypertension 18.

3. Nagele U, Hagele EO, Sauer G, Wiedemann E, Lehmann P, et al. (1984) Reagent for the enzymatic determination of serum total triglycerides with improved lipolytic efficiency. J Clin Chem Clin Biochem 22: 165-174.

4. Warnick GR, Benderson J, Albers JJ (1982) Dextran sulfate-Mg2+ precipitation procedure for quantitation of high- density-lipoprotein cholesterol. Clin Chem 28: 1379-1388.

5. Friedewald WT, Levy RI, Fredrickson DS (1972) Estimation of the concentration of low-density lipoprotein cholesterol in plasma, without use of the preparative ultracentrifuge. Clin Chem 18: 499-501.

6. Purcell S, Neale B, Todd-Brown K, Thomas L, Ferreira MAR, et al. (2007) PLINK: A Tool Set for Whole-Genome Association and Population-Based Linkage Analyses. Am J Hum Genet 81: 559-575.

7. Price AL, Patterson NJ, Plenge RM, Weinblatt ME, Shadick NA, et al. (2006) Principal components analysis corrects for stratification in genome-wide association studies. Nat Genet 38: 904-909.

8. Friedman GD, Cutter GR, Donahue RP, Hughes GH, Hulley SB, et al. (1988) CARDIA: Study design, recruitment and some characteristics of the examined subjects. J Clin Epidemiol 41: 1105-1116.

9. Warnick G (1986) Enzymatic methods for quantification of lipoprotein lipids. Methods Enzymol 129: 101-123.

10. Fried LP, Borhani NO, Enright P, Furberg CD, Gardin JM, et al. (1991) The Cardiovascular Health Study: design and rationale. Ann Epidemiol 3: 263-276.

11. Cushman M, Cornell ES, Howard PR, Bovill EG, Tracy RP (1995) Laboratory methods and quality assurance in the Cardiovascular Health Study. Clin Chem 41: 264-270.

12. Psaty BM, O'Donnell CJ, Gudnason V, Lunetta KL, Folsom AR, et al. (2009) Cohorts for Heart and Aging Research in Genomic Epidemiology (CHARGE) Consortium: Design of Prospective Meta-Analyses of Genome-Wide Association Studies From 5 Cohorts. Circ Cardiovasc Genet 2: 73-80.

13. Lee ET, Welty TK, Fabsitz R, Cowan LD, Le NA, et al. (1990) The Strong Heart Study. A study of cardiovascular disease in American Indians: design and methods. Am J Epidemiol 132: 1141-1155.

14. North KE, Howard BV, Welty TK, Best LG, Lee ET, et al. (2003) Genetic and Environmental Contributions to Cardiovascular Disease Risk in American Indians. Am. J. Epidemiol. 157: 303-314.

15. Centers for Disease Control and Prevention (2002) National Health and Nutrition Examination Survey III (NHANES) DNA Specimens: Guidelines for Proposals to Use Samples and Proposed Cost Schedule. Federal Register 67: 51585-51589.

16. Keebler ME, Sanders CL, Surti A, Guiducci C, Burtt NP, et al. (2009) Association of Blood Lipids With Common DNA Sequence Variants at 19 Genetic Loci in the Multiethnic United States National Health and Nutrition Examination Survey III. Circ Cardiovasc Genet 2: 238-243.

17. Kolonel LN, Altshuler D, Henderson BE (2004) The multiethnic cohort study: exploring genes, lifestyle and cancer risk. Nat. Rev. Cancer 4: 519-527.

18. Anderson GL, Manson J, Wallace R, Lund B, Hall D, et al. (2003) Implementation of the women's health initiative study design. Annals of Epidemiology 13: S5-S17.

19. (1998) Design of the Women's Health Initiative Clinical Trial and Observational Study. Controlled Clinical Trials 19: 61-109.
