# Supplementary material for: Genetic Determinants of Lipid Traits in Diverse Populations from the Population Architecture using Genomics and Epidemiology (PAGE) Study
Source: PLoS Genet. 2011 Jun 30;7(6):e1002138. doi: 10.1371/journal.pgen.1002138 (PMC3128106; doi:10.1371/journal.pgen.1002138)
Supplement: Supplementary file 16 [file pgen.1002138.s003.docx]

**Figure S3. Venn diagrams representing the overlap of significant associations (p<0.05) across the four major PAGE populations (European Americans, African Americans, Native Americans, and Mexican Americans/Hispanics, for the three lipid traits (HDL-C, LDL-C, and TG).**

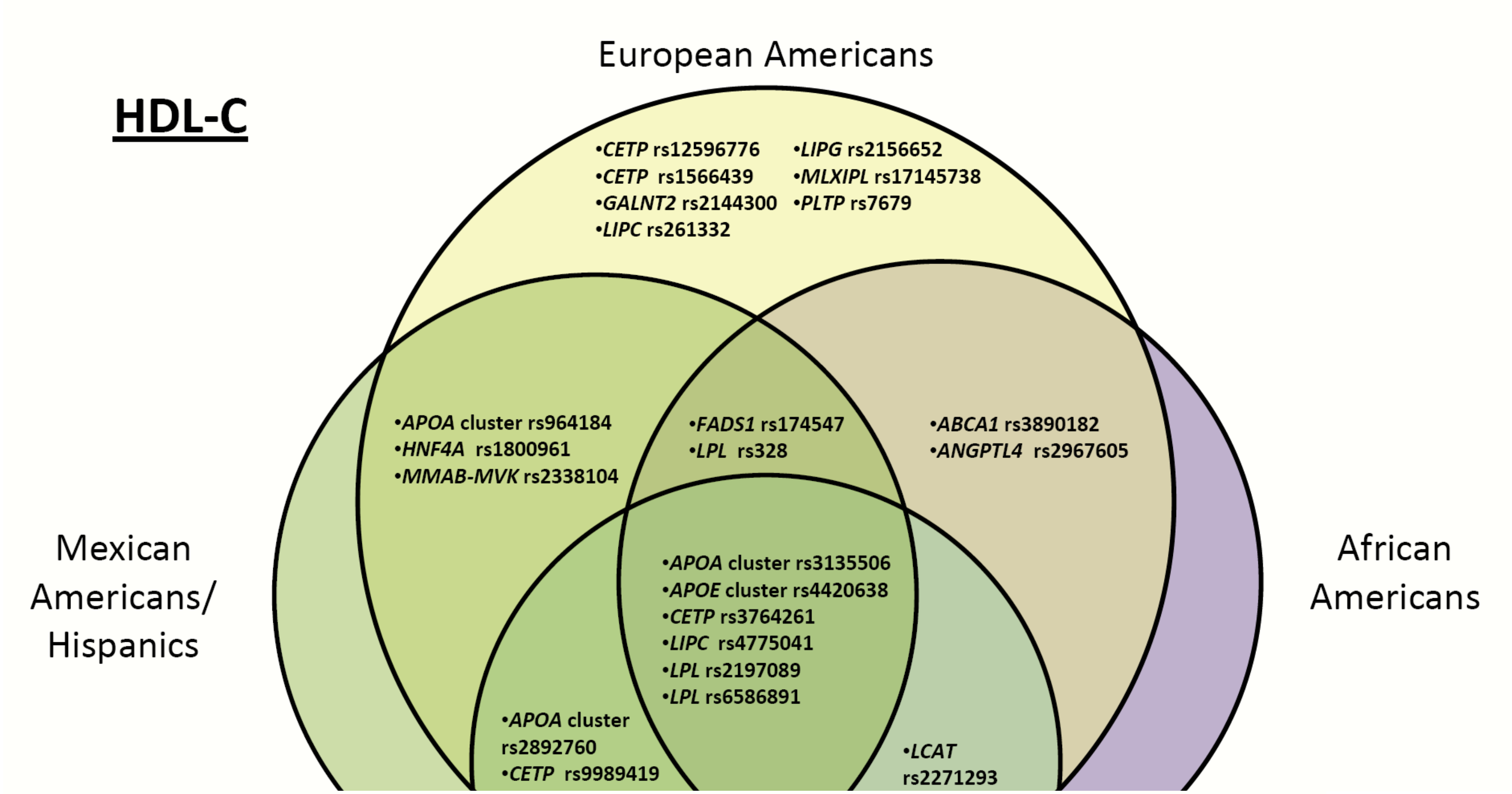


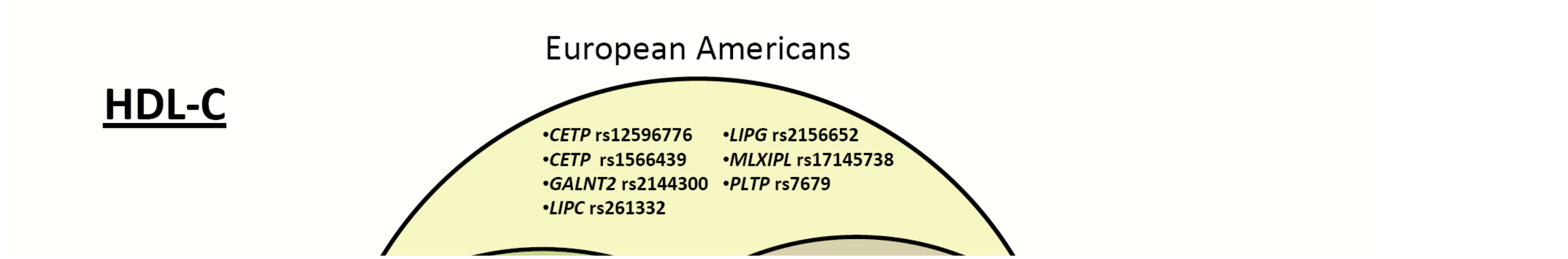

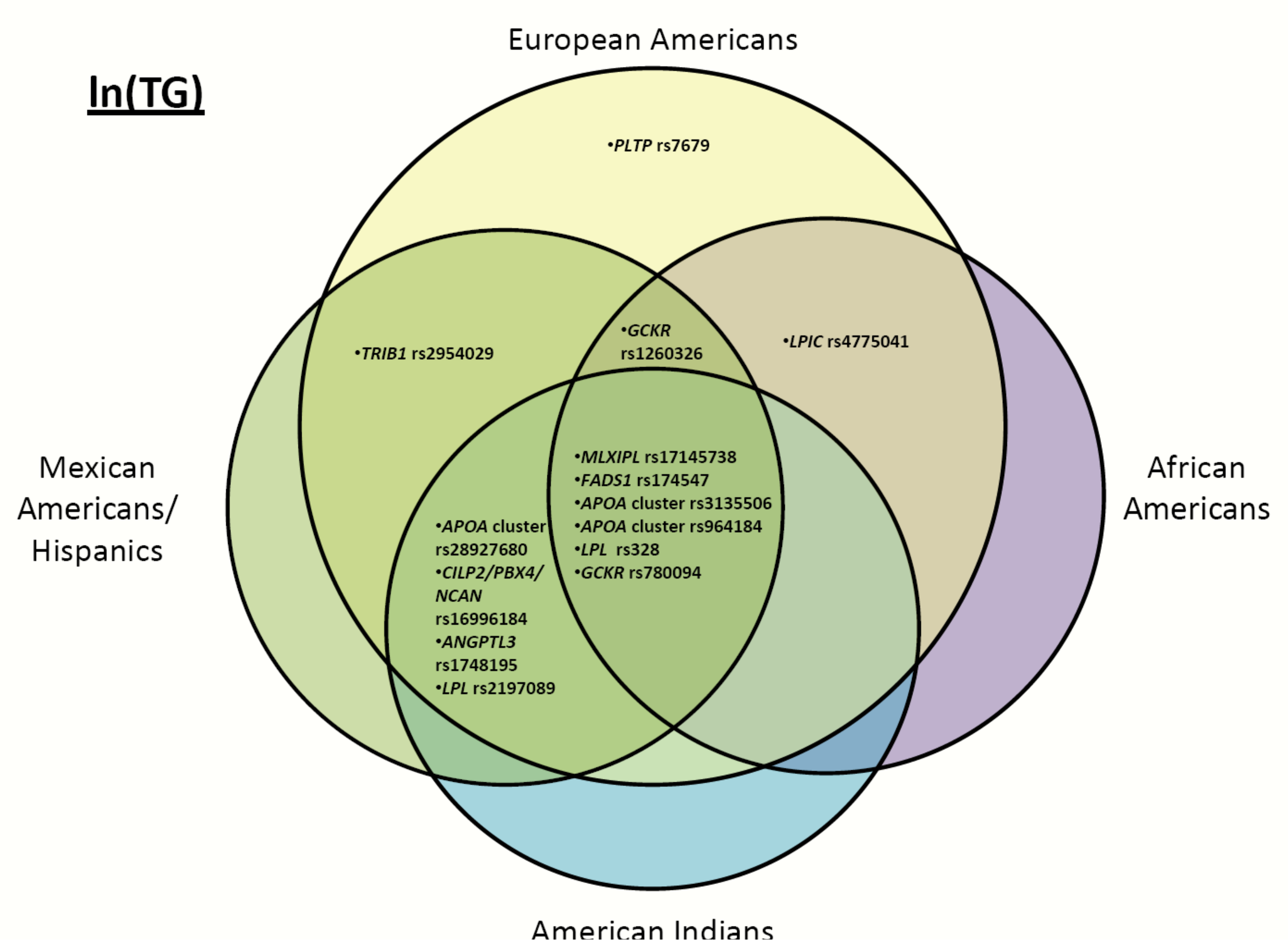
ln(TG)
European Americans
•PLTP rs7679
•TRIB1 rs2954029
•GCKR rs1260326
•LPIC rs4775041
Mexican Americans/ Hispanics
African Americans
•MLXIPL rs17145738
•FADS1 rs174547
•APOA cluster rs3135506
•APOA cluster rs964184
•LPL rs328
•GCKR rs780094
•APOA cluster rs28927680
•CILP2/PBX4/ NCAN rs16996184
•ANGPTL3 rs1748195
•LPL rs2197089
American Indians
